# Chromatin Accessibility and Transcriptomic Alterations in Murine Ovarian Granulosa Cells upon Deoxynivalenol Exposure

**DOI:** 10.3390/cells10112818

**Published:** 2021-10-20

**Authors:** Hairui Fan, Zhanshi Ren, Chao Xu, Haifei Wang, Zhengchang Wu, Zia ur Rehman, Shenglong Wu, Ming-an Sun, Wenbin Bao

**Affiliations:** 1 Key Laboratory for Animal Genetics, Breeding, Reproduction and Molecular Design, College of Animal Science and Technology, Yangzhou University, Yangzhou 225009, China; DX120180095@yzu.edu.cn (H.F.); Renzhanshi0422@163.com (Z.R.); zhongchaoxu@163.com (C.X.); wanghaiffei@126.com (H.W.); zcwu@yzu.edu.cn (Z.W.); slwu@yzu.edu.cn (S.W.); 2 Joint International Research Laboratory of Agriculture & Agri-Product Safety, The Ministry of Education of China, Yangzhou University, Yangzhou 225009, China; 3 Faculty of Animal Husbandry and Veterinary Sciences, College of Veterinary Sciences, The University of Agriculture Peshawar, Peshawar 25000, Pakistan; drzia80@aup.edu.pk; 4 Institute of Comparative Medicine, College of Veterinary Medicine, Yangzhou University, Yangzhou 225009, China

**Keywords:** ovarian granulosa cells, mycotoxin, chromatin accessibility, gene expression, cytotoxicity

## Abstract

Deoxynivalenol (DON) is a common environmental toxin that is secreted by fusarium fungi that frequently contaminates feedstuff and food. While the detrimental effects of DON on human and animal reproductive systems have been well recognized, the underlying mechanism remains poorly understood. Ovarian granulosa cells (GCs), which surround oocytes, are crucial for regulating oocyte development, mainly through the secretion of hormones such as estrogen and progesterone. Using an in vitro model of murine GCs, we characterized the cytotoxic effects of DON and profiled genome-wide chromatin accessibility and transcriptomic alterations after DON exposure. Our results suggest that DON can induce decreased viability and growth, increased apoptosis rate, and disrupted hormone secretion. In total, 2533 differentially accessible loci and 2675 differentially expressed genes were identified that were associated with Hippo, Wnt, steroid biosynthesis, sulfur metabolism, and inflammation-related pathways. DON-induced genes usually have a concurrently increased occupancy of active histone modifications H3K4me3 and H3K27ac in their promoters. Integrative analyses identified 35 putative directly affected genes including Adrb2 and Fshr, which are key regulators of follicular growth, and revealed that regions with increased chromatin accessibility are enriched with the binding motifs for NR5A1 and NR5A2, which are important for GCs. Moreover, DON-induced inflammatory response is due to the activation of the NF-κB and MAPK signaling pathways. Overall, our results provide novel insights into the regulatory elements, genes, and key pathways underlying the response of ovarian GCs to DON cytotoxicity.

## 1. Introduction

During the development of follicles in the mammalian ovary, layers of granulosa cells (GCs) surround the oocyte, which maintain the status of cumulus–oocyte complexes (COCs) for a period. Ovarian GCs play a key role in regulating the development of oocytes through the special structure of paracrine and junctional interactions between GCs and oocytes [1]. For example, GCs regulate follicular selection and atresia [2], provide substrate to the growing oocytes [3], and affect follicular selection and atresia [2]. In particular, GCs are the major source for several types of steroid hormones, such as estrogen and progesterone, which are essential for follicle development [4,5]. Given the central role of GCs in ovaries, in vitro cultured GCs serve as a widely used model to study female reproduction.

Deoxynivalenol (DON), also known as Vomitoxin, is the main mycotoxin secreted by the fusarium fungus, which frequently contaminates cereal crops that are the main food for human and livestock [6,7]. DON is resistant to high temperatures and mild acids and can be rapidly absorbed by the gastrointestinal tract and can reach various organs through blood circulation [8,9]. Therefore, DON contaminations threaten human health and animal husbandry, including the development of oocytes and GCs [8]. DON can cause nausea [10], diarrhea, vomiting, leukocytosis, and hemorrhage that sometimes ultimately result in death [11]. DON can also increase proinflammatory gene expression [12,13] and disrupt mitochondrial function [14,15], oxidative stress [16], and cell apoptosis [17]. Meanwhile, DON is also a potential modulator of human steroidogenesis [6]. Importantly, increasing evidence suggests that DON impairs reproductive function: it inhibits the proliferation of ovarian GCs [18] and endometrial cells [19] and alters the synthesis of testosterone, progesterone, and estradiol in ovarian GCs [20,21], which adversely affect oocyte maturation and embryo development [22,23,24]. However, the molecular mechanisms underlying the cytotoxic effects of DON on the reproductive system remain to be clarified.

The chromatin structure can define the mechanistic phenomena by which the interactions between transcription factors (TFs) and their cognate regulatory regions occur [25,26]. Nucleosome-depleted regions or open chromatin regions allow TFs to bind cis-regulatory elements and activate gene transcription, while closed chromatin regions hinder the access of TFs [27]. The assay for transposase accessible chromatin with high-throughput sequencing (ATAC-seq) is a powerful technique to map chromatin accessibility at a genome-wide scale [28], which has been widely used to capture open chromatin regions (e.g., active promoters and enhancers) across the genome [27,29]. Moreover, integrative analyses of ATAC-Seq, RNA-Seq, and TF binding motif data enable the understanding of the link between chromatin accessibility and gene transcription and allow the inference of possible core TFs underlying the gene regulatory network [30]. To date, the toxicity of DON exposure on ovarian GCs has not been directly explored regarding the genome-wide remodeling of the chromatin accessibility landscape. 

In this study, we characterize the chromatin accessibility and transcriptional landscapes in murine ovarian GCs upon DON exposure. Integrative analysis allowed us to determine the landscape of regulatory elements, binding events, and putative TFs that might be underlying the cellular response of GCs to the DON toxin. Taken together, our findings contribute to future research attempting to discover biomarkers and drug targets for mammalian reproductive diseases caused by DON contamination.

## 2. Materials and Methods

### 2.1. Chemicals and Reagents

DON (D0156; 5 mg) were purchased from Sigma-Aldrich (St. Louis, MO, USA). FBS and DMEM-F12 were obtained from Gibco BRL (Carlsbad, CA, USA). Anti-Bax (ET1603-34), anti-Bcl-2 (ET1603-11), anti-caspase 3 (ER30804), anti-cleaved caspase 3 (ET1608-64), anti-rabbit immunoglobulin G (IgG)-horseradish peroxidase (HRP; HA1031), and Alexa Fluor 555–conjugated antibody (HA1118) antibodies were purchased form HuaAn Biotechnology (Hangzhou, China). Anti-ERK1 (ab109282), anti-phospho-ERK (ab201015), anti-p65 (ab32536), anti phospho-p65 (ab76302), anti-P38 (ab170099), anti-JNK (ab179461), anti-phospho-JNK, anti-phospho-P38 (ab178867), anti-IκB (ab32518), anti-phospho-IκB (ab133462), anti-H3K4me3 (ab8580) and anti-H4K27ac (ab4729), and anti-α-tubulin (ab7291) antibodies were obtained from Abcam Ltd. (Cambridge, UK). Anti-HSP90 (60318) and anti-GAPDH (10494-1-AP) antibodies were obtained from Proteintech Ltd. (Rosemont, IL, USA).

### 2.2. Isolation and Culture of Ovarian GCs

**Figure 1:**
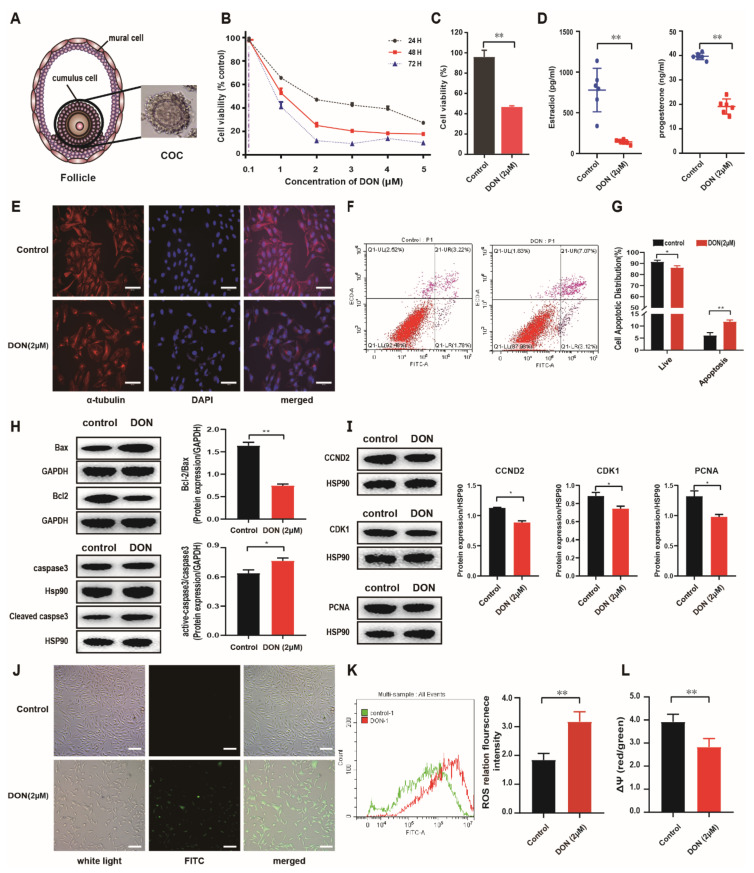
The toxicity effects of DON on murine ovarian granulosa cells. (**A**) The mode pattern of mammalian follicle and cumulus–oocyte complex (COC). (**B**) Viability of cells treated with the different concentrations of DON (0.1, 1, 2, 3, 4, and 5 μM) and cultured for different periods of time (24, 48, and 72 h) (n = 3). (**C**) Viability of cells at 2 μM of DON for 24 h. (**D**) Estradiol and progesterone were measured by radioimmunoassay in the media after treatment with DON at 2 μM for 24 h (n = 6), Each bar represents the mean ± SEM. (**E**) Morphological differences between control and DON treated group (n = 6). DON-treated and nontreated GCs were stained with anti-α-tubulin antibody (red), and DNA was stained with DAPI (blue). Scale bar: 200 μm. (**F**) Apoptosis of ovarian GCs was evaluated by measurement of Annexin-V using flow cytometry (n = 3). (**G**) The figure shows a representative staining, and the numbers in the quadrants indicate the percentage of cells within the respective subpopulations (n = 3). (**H**) Western blotting analysis of the changes in the protein levels of related apoptosis factors (Bax, Bcl-2, cleaved caspase-3, caspase-3) (n = 3). (**I**) Western blotting analysis of the changes in the protein level of functional genes linked to proliferation (PCNA, CDK1, and CCND2) (n = 3). (**J**) Representative images of ROS levels in the control and DON-exposed mouse ovarian GCs. The fluorescence intensity (green) shows the level of ROS. Scale bar: 200 μm. (**K**) ROS relation fluorescence intensity between control and DON-treated group (n = 3). (**L**) ΔΨm differences between control and DON treated group. The ratio of the red over green fluorescence intensity by flow cytometry represents the quantitative ΔΨm in each group (n = 3). * *p* < 0.05 and ** *p* < 0.01 compared with the control group (*t*-test).

Four-week-old ICR female mice were used in this study and were housed in a temperature-controlled room with a 12 h light-dark cycle (ON at 8:00 a.m., OFF at 8:00 p.m.) and were fed with a regular diet and water. In total, 120 ICR female mice were used in the experiments. Mice were injected with 6 IU pregnant mare serum gonadotrophin (Ningbo Hormone Products Co, Zhejiang, China), and then their ovaries were collected 44–48 h later. Ovarian COCs (Figure 1A) were collected from these ovaries and were washed three times in PBS. The GCs were dispersed with DMEM/F12 supplemented with 10% FBS and were seeded at a density of 5 × 10^4^ cells/mL in 96-well plates and 6-well plates. The cells were incubated in 5% CO_2_ at 37 °C for 24 h before further treatment, and the culture medium was refreshed every 24 h.

### 2.3. Treatment of GCs with DON and Assessment of Cell Viability

DON was diluted in the culture medium with final concentrations of 0.1, 1, 2, 3, 4, and 5 μM. After the GCs grew to approximately 70% confluency, they were treated with different concentrations and different co-incubation times (24, 48, and 72 h). Cell viability was checked via cell counting Kit-8, according to the protocols provided by the manufacturer (Dojindo Laboratories, Kumamoto, Tokyo, Japan). The optical density measurements were conducted at a wavelength of 450 nm on a Tecan Infinite 200 microplate reader (Sunrise, Tecan, Switzerland). The GCs cells of the from the experimental group were treated with 2 μM DON for 24 h, and the control group was cultured in the medium with an equal volume of solvent. Six and three replicates were collected for the RNA-Seq and ATAC-Seq experiments, respectively.

### 2.4. Ovarian GCs Morphology and Radioimmunoassay

After being treated with 2 μM DON for 24 h, GCs were fixed for 30 min at room temperature (RT) in 4% PFA, blocked with 3% (*wt*/*vol*) BSA in PBS, and they were then incubated overnight at 4 °C with anti-α-tubulin. Signals were detected with Alexa Fluor 555–conjugated antibody, and DNA was stained with DAPI (Invitrogen). Fluorescent images were collected using a Leica fluorescence microscope (Leica, Wetzlar, Germany). The medium was collected to measure estradiol and progesterone using radioimmunoassay (RIA) reagents (Beijing North Institute of Biological Technology, Beijing, China). The sensibility of each hormone RIA kit was 5 pg/mL for estradiol and 0.01 ng/mL for progesterone.

### 2.5. Analyses of Apoptosis, Reactive Oxygen Species and Mitochondrial Membrane Potential by Flow Cytometry

GCs were collected to analyze apoptosis (Solarbio, Beijing, China), intracellular reactive oxygen species (ROS) (Solarbio, Beijing, China), and mitochondrial membrane potential (ΔΨm) (Beyotime, Jiangsu, China) using flow cytometry (Beckman Coulter, Brea, CA, USA); each measurement was performed three times with at least 20,000 cells. Fluorescent images were taken using a Leica fluorescence microscope (Leica, Wetzlar, Germany).

### 2.6. ATAC-Seq Library Preparation and Data Analysis

ATAC-Seq was performed following a previous study [28]. In brief, after native nuclei were purified, about 50,000 nuclei were resuspended in the Tn5 transposase reaction mix and were incubated for 30 min at 37 °C. The transposed DNA fragments were purified immediately using the MinElute PCR Purification Kit (Qiagen, Beijing, China), amplified using 1×NEBNext High-Fidelity PCR Master Mix (New England Biolabs, MA, USA), and then purified using MinElute PCR Purification Kit (Qiagen, Beijing, China). These purified samples were sequenced as 150 bp paired-end reads on the Illumina Novaseq6000 instrument by Jiayin Biotechnology Institute (Shanghai, China).

After adaptor sequence removal, the reads were aligned to the mouse reference genome (Release mm10) using BWA [31]. Peak calling was performed using MACS2 [32] with cutoff *q*-value < 0.05. The read distributions within +/− 3kb of transcription start sites (TSSs) were calculated using the computeMatrix function of DeepTools [33]. Peak files across samples were merged with the merge function of the BEDTools [34], and the read counts over each peak across samples were determined using the multicov function of the BEDTools. DESeq2 was used to identify differential accessible peaks with cut-off |log2 fold change| ≥ 1 and *p*-value < 0.05. The findMotifsGenome.pl script from HOMER [35] was used for motif analysis.

### 2.7. RNA-Seq Library Preparation and Data Analysis

Trizol reagent (Qiagen) was used for total RNA isolation. The mRNA was purified from total RNA using poly-T oligo-attached magnetic beads, and then the fragmented mRNA was used for the synthesis of cDNA via reverse transcription followed by purification. The PCR-amplified cDNA purification was conducted via an AMPure XP system (Beckman Coulter, Beverly, USA), and the obtained cDNA fragments were 250–300 bp long. The libraries were sequenced as 150 bp paired-end reads on the Illumina Novaseq6000 platform. Raw reads were preprocessed with in-house Perl scripts to the trim adapter, ploy-N, and low-quality bases, and were then aligned to the mouse reference genome (Release mm10) using STAR [36]. The read counts for each gene were calculated via HTSeq [37]. FPKM (fragments per kilobase of transcript per millions of mapped fragments) were calculated using Stringtie. DESeq2 [38] was employed to identify differentially expressing genes (DEGs) with cutoff: |log2 fold change| ≥ 1 and adjusted *p*-value < 0.05.

### 2.8. Integrative Analysis of ATAC-Seq and RNA-Seq Data

Integrative analysis of ATAC-Seq and RNA-Seq data was performed to identify the TFs that made a considerable contribution to the regulations according to the chromatin opening region and regulatory mechanism of these TFs on the related downstream genes. The overlap between the upregulated genes and chromatin regions with increased accessibility was calculated, and then Fisher’s Exact Test was applied to determine if their overlap was significant. The overlap between the downregulated genes and chromatin regions with decreased accessibility was determined and tested with the same procedure.

### 2.9. Gene Ontology, Pathway and PPI Network Analyses

Gene ontology (GO) analysis of the accessible chromatin regions was performed with GREAT [39]. Pathway analysis was performed to find the significant pathway of the annotated genes according to the Kyoto Encyclopedia of Genes and Genomes (KEGG) database. Fisher’s Exact Test was used to identify the significant pathway and GO categories with a *p*-value < 0.05 as cutoff [40]. Protein–protein interaction (PPI) enrichment analysis was performed using the following databases: BioGRID [41], OmniPath [42]. Metascape [43], and Cytoscape (v3.5.1, NRNB, Bethesda, MD, USA) [44] were used to build the PPI network, and a confidence score > 0.7 was set as the cutoff. Molecular complex detection (MCODE) [45] was used to screen the modules of the PPI network using the following parameters: degree cutoff = 2, node score cutoff = 0.2, k-core = 2, and maximum depth = 100.

### 2.10. RT-PCR, ChIP-qPCR and Western Blotting

The HiScript^®^ Reverse Transcriptase kit (Vazyme Biotech Co., Ltd., Nanjing, China) was used to produce cDNA. Then, qPCR reactions were performed using the SYBR Green master mix (Vazyme Biotech co., Ltd., Nanjing, China) in an ABI StepONEPlus Real-Time PCR System (Applied Biosystems, Foster City, CA, USA). The mouse *β-actin* was selected as an internal control. Each gene was performed in triplicate, and the relative quantitative of gene expression was calculated using the 2^-ΔΔCt^ method [46]. All of the primers used for qPCR are listed in Table S1.

For ChIP-qPCR, GCs were fixed with 1% formaldehyde for 10 min, quenched with 2.5 M of Glycine for 5 min, and sonicated to fragments of 500–700 bp in length. Subsequently, the chromatin fragments were incubated with anti-H3K4me3, anti-H3K27ac, and anti-rabbit IgG antibodies and were reverse-crosslinked. ChIP-DNA was purified for qPCR with primers for selected genes. *β-actin* was used as the internal reference. The primers are listed in Table S2.

For Western blotting, GCs were lysed with a RIPA buffer supplemented with protease and phosphatase inhibitors for 10 min on ice, scraped, and centrifuged at 14,000 rpm at 4 °C for 10 min. After being quantified using a BCA protein assay kit (Solarbio, Beijing, China), equal amounts (20 μg) of denatured proteins were loaded on 10% SDS-PAGE gel for electrophoresis, transferred to PVDF membranes (Immobilon, Darmstadt, Germany), and then blocked with 5% skim milk at room temperature for 1 h. PVDF membranes were incubated overnight at 4 °C with primary antibodies (1:1000) and were then washed with 1×TBST (Solarbio, Beijing, China) before incubation with secondary antibodies (1:5000). Protein bands were visualized using Luminol/Enhancer Reagent (New Cell & Molecular Biotech, Suzhou, China) and were then exposed with FluorChem FC3 system (Protein-Simple, CA, USA). Finally, the relative integrated density of each band was digitized with the FluorChem FC3 system. GAPDH or HSP90 was used as internal control.

### 2.11. Statistical Analysis

All of the results were expressed as the mean ± SEM with three separate experiments, and each trial was performed using triplicate samples. The comparison between the treatment groups and the control group was performed using unpaired Student’s *t*-tests. Statistical analyses were performed and visualized using GraphPad Prism 8.0.1 (GraphPad, La Jolla, CA, USA) with the significance level set as 0.05.

## 3. Results

### 3.1. Severe Toxicity Effect of DON on Murine Ovarian GCs

To evaluate the toxicity effect of DON, we treated murine ovarian GCs with six concentrations (0.1, 1, 2, 3, 4, 5 μM) of DON for 24 h, 48 h, and 72 h. The results clearly demonstrate that a higher concentration and longer treatment time result in lower cell viability (Figure 1B). When treated with 2 μM DON for 24 h, the cell viability was dramatically decreased by about 50% relative to the control (*p* < 0.01) (Figure 1C); therefore, this concentration and treatment time was used throughout this study. Given the important role of GCs in hormone secretion, we determined the effect of DON on the secretion levels of estrogen and progesterone, which are essential for follicular growth. As expected, both the levels of estrogen (*p* < 0.01) and progesterone (*p* < 0.01) were severely impaired upon DON treatment (Figure 1D). Furthermore, DON treatment reduced the cell confluency and caused abnormal morphology, as represented by the spread and relative irregular shape (Figure 1E).

**Figure 2:**
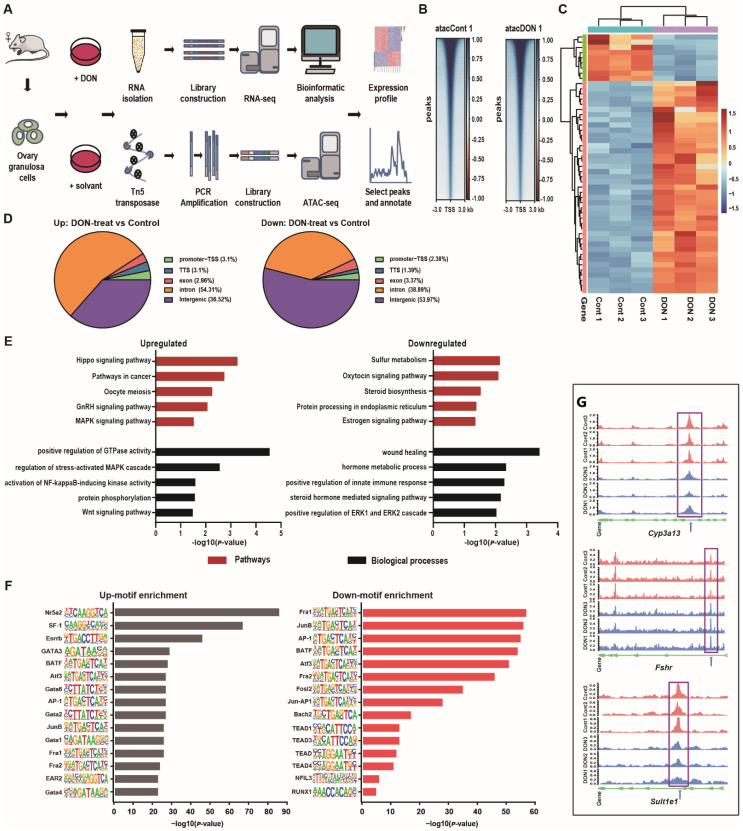
ATAC-seq data quality metrics and pathway analysis. (**A**) Overview of the experimental and data analysis workflow. (**B**) The ATAC-seq signal enrichment around the transcription start sites (TSSs) of DON-treated and control group. (**C**) Hierarchical clustering of peak enrichment patterns between DON-treated and control groups. (**D**) The genome-wide distribution of the peaks. The genome-wide functional regions were divided into promoter, downstream TTS, coding exon, intron, and distal intergenic regions. (**E**) Signaling pathway associated with chromatin accessibility in ovarian GCs upon DON exposure. (**F**) The top 15 of upregulated and downregulated motif enrichment. (**G**) Tracks for DON-treated samples at the Cyp3a13, Fshr, and Sult1e1 genes with predicted AP-1 binding sites. The predicted AP-1 binding sites are shown with a blue arrow.

We further examined whether DON could induce apoptosis in GCs. An Annexin V-FITC/PI staining assay confirmed that DON significantly increased the ratio of apoptotic cells by about 2-fold (*p* < 0.01) (Figure 1F,G). The inspection of several apoptotic factors (i.e., Bax, caspase 3, and cleaved caspase 3) and antiapoptotic factors (Bcl-2) revealed decreased Bcl-2/Bax ratio (*p* < 0.01) and an increased cleaved caspase 3/caspase 3 ratio (*p* < 0.05) (Figure 1H). We further examined the levels of PCNA, CDK1, and CCND2, which are the indicators of cell proliferation; as expected, they were all significantly reduced after DON treatment (*p* < 0.05) (Figure 2I). Together, these results suggest that DON significantly induced apoptosis in GCs.

Due to previous studies that suggest that DON induces oxidative stress to impair cellular function [23], the ROS levels and mitochondrial membrane potential (ΔΨm) in GCs after DON exposure were measured. In agreement with previous studies for other cell types, our results indicated that DON also induced oxidative stress in ovarian GCs, as indicated by increased ROS production (*p* < 0.01) (Figure 1J,K) and decreased ΔΨm (*p* < 0.01) (Figure 1L). Taken together, we demonstrated that the toxicity of DON on GCs occurs in multiple aspects regarding cell viability, hormone secretion, morphology, oxidative stress, and apoptosis.

### 3.2. Numerous Genomic Loci Have Altered Chromatin Accessibility upon DON Exposure

To further clarify the molecular mechanism underlying the toxicity of DON on GCs, we established an in vitro model of ovarian GCs and applied ATAC-Seq to profile the genome-wide chromatin accessibility landscape before and after DON treatment (Figure 2A). The generated data and called peaks are summarized in Tables S3 and S4. Initial analyses confirmed high chromatin accessibility flanking transcription start sites (TSSs) and clear periodicity of chromatin fragment size (Figure 2B, Figure S1). Principal component analysis (PCA) further revealed that the samples are well clustered by group (Figure S2), altogether suggesting that our data are of good quality.

We next determined the genomic loci with altered accessibility after DON treatment and asked how they were related to the cellular response. Thousands of genomic loci with increased (n = 2029) or decreased (n = 504) accessibility were identified (Figure 2C, Tables S5 and S6), with the majority having increased chromatin accessibility and residing in intronic and intergenic regions (Figure 2D). Interestingly, genomic loci with increased accessibility are associated with Hippo, GnRH and MAPK, NF-κB, Wnt, and cancer related signaling pathways (Figure 2E), which is not surprising given previous knowledge that the toxic metabolism of DON is related to multiple signaling pathways [9]. Notably, the activation of the Hippo pathway is known to inhibit the proliferation of normal cells and tumors [47,48]. In contrast, loci with decreased accessibility are highly associated with hormone synthesis, wound healing, positive regulation of innate immune response, and ERK1 and ERK2 cascade (Figure 2E, Tables S7–S10). These results are consistent with the aforementioned observation that DON affects the viability, proliferation, and hormone secretion in GCs (Figure 1).

To uncover putative TFs that mediate DON induced cellular response, we performed motif enrichment analysis for the chromatin regions with altered accessibility (Figure 2F), focusing on those with increased chromatin accessibility after DON treatment. Among the enriched motifs, Nr5a2 and SF1 have been previously reported to participate in regulating the maturation of ovarian follicles, ovulation, and pregnancy [49,50]. Moreover, AP-1 motif was determined in the open chromatin region in these genes, including Fshr, CYP3a13, and Sult1e1 (Figure 2G). Some of these TFs may play core roles in regulating the cellular response of GCs in response to DON exposure.

### 3.3. Thousands of Genes Have Altered Transcription in Response to DON Exposure

**Figure 3:**
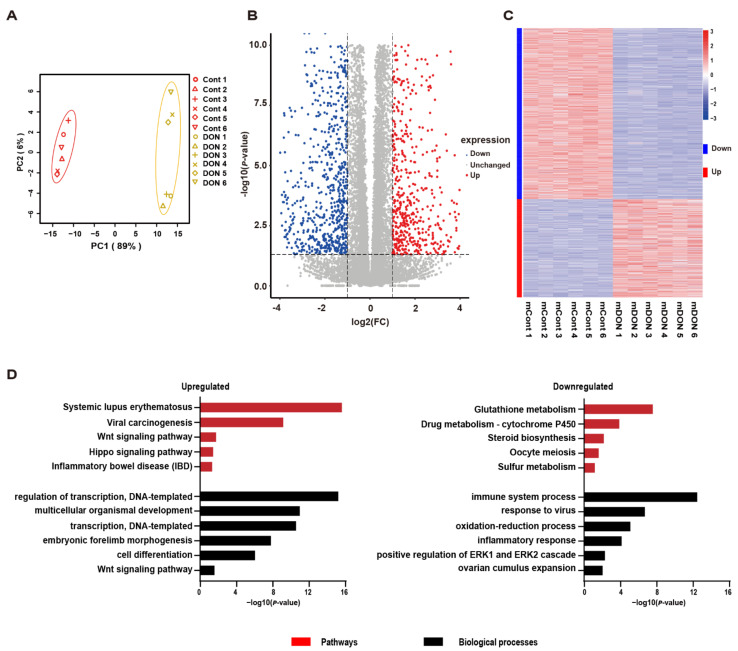
The analysis of differentially expressed genes between the DON-treated and control groups. (**A**) The principal components analysis (PCA) plot of RNA-seq samples. (**B**) Volcano plot of differential expression profiles between the DON-treated and control groups. (**C**) Heatmap of differential expression profiles between the DON-treated and control groups. (**D**) The top enriched pathway and GO terms for the differentially expressed genes. The rich factor is defined as the ratio of the number of differentially expressed genes enriched in the pathway to the number of annotated genes.

To uncover the genes involved in the cellular response of GCs to DON toxicity, we applied RNA-Seq to characterize the transcriptomic alterations upon DON exposure. The twelve samples (six per group) generated 594.3 million high-quality read pairs (Table S11), and PCA analysis revealed that these samples were well clustered by group (Figure 3A). Thousands of DEGs were identified, including 967 upregulated and 1,699 downregulated ones (Figure 3B–C, Table S12). GO and pathway enrichment analyses demonstrated that the DON-induced genes were highly associated with systemic lupus erythematosus, viral carcinogenesis, the Wnt-signaling pathway, Hippo related signaling pathway, transcription regulation, and innate immune response. In contrast, DON repressed genes that are associated with glutathione metabolism, drug metabolism-cytochrome P450, teroid biosynthesis, oocyte meiosis, sulfur metabolism, immune system processes, oxidation-reduction processes, and the positive regulation of the ERK1 and ERK2 cascade (Figure 3D and Tables S13–S16). These results are largely consistent with those for the genomic loci with altered chromatin accessibility.

### 3.4. Integrative Analysis Identifies Putative Direct Targets in Response to DON Toxicity

**Figure 4:**
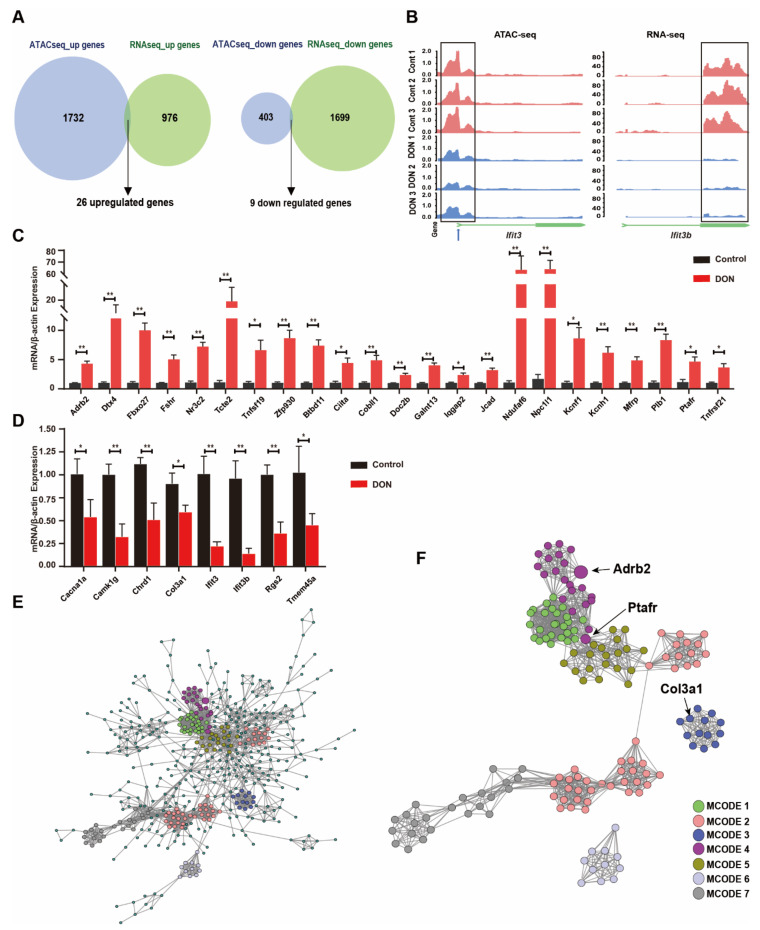
The association between the specific chromatin-accessible regions and gene expression in murine ovarian GCs upon DON exposure. (**A**) Venn diagram showing genes associated with the chromatin-accessible regions and differentially expressed genes. (**B**) Changes in chromatin accessibility downstream and expressive level of the Ifit3b gene. The predicted TFs binding sites are shown with a blue arrow. (**C**) Realtime RT-PCR analysis of the relative expression of the DGEs which collaborative filtering from RNA-seq data and ATAC-seq data. Each bar represents the mean ± SEM. * *p* < 0.05, ** *p* < 0.01 compared with the control group (*t*-test). (**D**) Protein–protein interaction (PPI) network of proteins encoded by differentially expressed genes (DEGs). (**E**,**F**) Modules selected from PPI network using MCODE. Degrees > 6 was set as the cutoff criterion. Nodes represent DEGs; lines represent interaction relationships between nodes.

To identify the genes that are putatively under direct regulation upon DON exposure, we conducted integrative analyses of the ATAC-seq and RNA-seq data. By comparing DEGs and differential accessible loci, we investigated 26 DON-induced and 9 DON-repressed genes that are associated with at least one locus with altered chromatin accessibility of the same direction (Figure 4A), among them *Adrb2*, *Dtx4*, *Fbxo27*, *Fshr*, *Nr3c2*, *Plb1*, *Tcte2*, *Tnfrsf19*, and *Zfp930,* which are DON-induced, and *Cacna1a*, *Camk1g*, *Chrdl1*, *Col3a1*, *Ifit3*, *Ifit3b*, *Rgs2*, *Slc6a12*, and *Tmem4*5a, which are DON-repressed (Table S17). Closer inspection of the Ifit3b gene confirmed concurrently decreased gene expression and chromatin accessibility (Figure 4B), suggesting that the chromatin accessibility makes considerable contributions to gene expression, at least for some of these genes, in response to DON exposure. The differential expression for 88.6% (31/35) of these genes was successfully validated by qRT-PCR (Figure 4C,D). Therefore, these genes are the most possible direct targets of DON in GCs, which may be valuable for further investigation. To further identify the hub genes, we performed PPI network analysis for the RNA-seq identified DEGs (Figure 4E). MCODE was used to identify densely connected network components. Interestingly, among the significant MCODEs, MCODE4 contained Adrb2 and Ptafr, and MCODE3 contained Col3a1, which the genes altered significantly in terms of chromatin accessibility and expression level (Figure 4F). Therefore, we speculate that Adrb2, Ptafr, and Col3a1 may play a potentially key regulatory role in DON toxicity in GCs.

### 3.5. Active Histone Marks Are Enriched in Open Chromatin Regions in Murine Ovarian GCs upon DON Exposure

**Figure 5:**
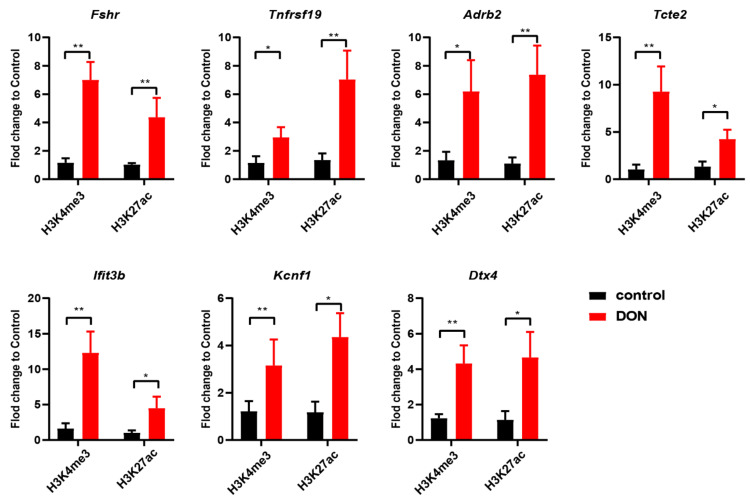
Histone modifications at the accessible chromatin regions. The fold changes of histone H3K4me3 and H3K27ac are determined by ChIP-qPCR in chromatin-accessible regions of Fshr, Tnfrsf19, Adrb2, Tcte2, Ifit3b, Kcnf1, and Dtx4. Each bar represents the mean ± SD, n = 3. * *p* < 0.05 and ** *p* < 0.01 compared with the control group (*t*-test).

Epigenetic modifications and gene transcription are tightly correlated, and several histone modifications have been widely used to annotate the activity of regulatory elements. In particular, H3K4me3 and H3K27ac are known to mark active promoters [51]. Focusing on the genomic loci that are adjacent to DON-induced genes and that have increased accessibility, we applied ChIP-qPCR to examine if they have altered occupancy of H3K4me3 and H3K27ac upon DON exposure. Our results demonstrate that all of the seven examined loci (*Adrb2*, *Fshr*, *Tnfrsf19*, *Tcte2*, *Dtx4*, *Ifit3b*, and *Kcnf1*) have increased occupancy of H3K4me3 and H3K27ac upon DON exposure (Figure 5). Together, these results indicate that the alterations of gene expression, chromatin accessibility, and histone modifications in ovarian GCs upon DON exposure are closely correlated.

### 3.6. DON-Induced Inflammatory Response via Activation of NF-κB and MAPK Signaling Pathways

**Figure 6:**
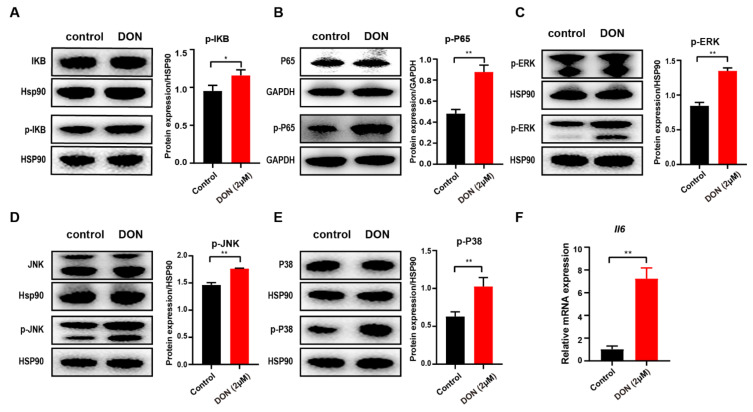
DON-induced NF-κB and MAPK activation in murine ovarian granulosa cells. Ovarian GCs were stimulated with DON for 24 h. (**A**) IkBα (1:1000 dilution) and p-IκBα (1:1000 dilution). (**B**) P65 (1:1000 dilution) and p-P65 (1:1000 dilution). (**C**) ERK (1:1000 dilution) and p-ERK (1:1000 dilution). (**D**) JNK (1:1000 dilution) and p-JNK (1:1000 dilution). (**E**) P38 (1:1000 dilution) and p-P38 (1:1000 dilution). (**F**) The relative gene expression of *Il6* in GCs with and without DON treatment. All data are shown as mean ± SD, n = 3. * *p* < 0.05 and ** *p* < 0.05 compared with the control.

Previous studies proved that NF-κB, which can be activated by various extracellular and intracellular stimuli, is involved in inflammatory and immune response and regulates the expression of proinflammatory mediators such as interleukin 6 (Il6) and TNF-α [52,53]. Interestingly, our results indicate that differentially expressed genes in GCs are also highly associated with the NF-κB and MAPK signaling pathways (Figure 2E). To further verify the inflammatory response of ovarian GCs induced by the toxicity of DON, we examined the protein abundance for the core genes from NF-κB and the MAPK signaling pathway. Regarding the NF-κB signaling pathway, DON remarkably induced phosphorylated-P65 and phosphorylated-IκB (Figure 6A,B). The protein abundances of the phosphorylation of extracellular regulated kinase (ERK), c-Jun N-terminal kinase (JNK), and p38 MAPKs were also significantly increased (Figure 6C–E). Notably, the RNA level of Il6 was also upregulated in GCs upon DON exposure, as revealed by RNA-seq and RT-PCR results (Figure 6F, Table S11). Taken together, these results indicate that DON toxicity could induce inflammation through the activation NF-κB and the phosphorylation of ERK, JNK, and p38 MAPKs in ovarian GCs.

## 4. Discussion

DON has attracted public attention due to its widespread occurrence in foodstuffs and its prominent toxicological effects in humans and animals. Importantly, the toxicity effects of DON on reproductive cells have been reported by multiple previous studies [19,23]. However, the molecular mechanism underlying DON-induced cytotoxicity and cellular response remains poorly understood. In this study, we established an in vitro model of mouse ovarian GCs to investigate the DON-induced toxicity effects and cellular response and performed ATAC-Seq and RNA-Seq to characterize the alterations of chromatin accessibility and the transcriptomic landscape upon DON exposure.

Several previous studies examined the effects of DON on different mammalian cell types. For example, DON increased the proportion of apoptotic cells and inhibited estradiol and progesterone secretion in bovine GCs [17]. DON induced apoptosis through the caspase-3 activation pathway, which resulted in a functional disorder in porcine hepatocytes [54], repressing cell growth by retarding cell cycles in the G2/M phase in human epithelial cells [55]. Our results in mouse GCs are consistent with these studies. Meanwhile, existing studies reported that DON could induce cellular oxidative stress by promoting ROS production in IPEC-J2 cells [56]. Our results indicate that DON can not only induce ROS production but can also reduce MMP. Our previous study suggested that melatonin could ameliorate DON-induced oxidative stress in murine ovarian GCs [57]. Based on these results, we speculate that the toxicity effects of DON on GCs occur in a multiple of aspects and deserves further investigation.

A recently developed ATAC-Seq technique enables the genome-wide profiling of chromatin accessibly, and its wide application has facilitated the understanding of the molecular mechanisms of numerous diseases [27,29,58,59]. Using ATAC-Seq, we identified 2533 differentially accessible loci that are associated with distinct biological pathways. Among them, the Hippo signal pathway has been proven to induce cell apoptosis [47], and inhibition of the Hippo signal transduction is promising for the treatment of ovarian disorders such as polycystic ovarian syndrome [48]. In addition, steroid hormone, estrogen, oocyte meiosis, ovarian steroidogenic, and the sulfur metabolism pathway were also significantly enriched, which are strongly associated with reproductive hormone synthesis. Moreover, inflammatory-related signaling pathways were enriched significantly. In addition, GO analysis showed that the regulation of stress-active MAPK cascade, activation of NF-κB inducing kinase activity, protein phosphorylation, and the Wnt signaling pathway were upregulated. These results showed that the alterations of chromatin accessibility in these signaling pathways might partly elucidate the mechanism of the phenotype of ovarian GCs induced by DON.

Integrative analysis of the chromatin accessibility and transcriptomic data identified 26 DON-induced genes and 9 DON-down genes. Herein, the majority different peaks of our dataset were annotated in intronic and intergenic regions, which are far from the gene promoter, and the underlying complexity and negative controls may have resulted in fewer genes after combined analysis. Perhaps other regulatory mechanisms were involved in this process, such as histone acetylation and methylation. Nevertheless, we have screened many hub genes. Among them, the *Adrb2*-mediated actions in the growth of primate follicles and the activation of Adrb2 triggers the proliferation of small follicle and induce *Fshr*, which is critical for follicular development [60]. The *Fshr* is mainly expressed in ovarian GCs in female animals and has been identified as playing a key role in the regulation of steroid synthesis and follicular proliferation. Herein, we identified significant upregulation after DON exposure, suggesting that the interaction of *Adrb2* and *Fshr* may participate in the response of ovarian GCs to DON exposure. Meanwhile, *Adrb2* could control inflammation through the rapid induction of *IL-10* [61]. Interestingly, the PPI network analysis of the identified DEGs revealed that Adrb2, Ptafr, and Col3a1 appears in the top MCODEs. Therefore, we speculate that they may play a potentially key regulatory role in protein–protein interaction under DON toxicity.

The binding motifs for several TFs are overrepresented in genomic regions with increased accessibility upon DON exposure. Among them, Nr5a2 is highly expressed in GCs of primary to preovulatory follicles [62], and the deletion of Nr5a2 caused early embryo lethality [63]. Duggavathi et al. reported that Nr5a2 is a necessary regulator of multiple mechanisms that are essential for the maturation of ovarian follicles and for ovulation [50]. Meanwhile, SF-1 (also known as Nr5a1), which also belongs to the NR5A family, is another important regulator of steroid hormones [64]. Based on these results, we speculate that these enriched TFs participated in the regulation of ovarian GCs response to DON toxicity. Enriched TF binding sites occur near the chromatin accessible regions of TSSs that are involved in the regulation of gene expression in ovarian GCs in response to DON toxicity. Meanwhile, the accessibility of regulatory regions during the chromatin remodeling processes involves nucleosome occupancy and histone modifications, and the histone modifications H3K4me3 and H3K27ac are crucial for the binding of TFs [27]. Herein, our results verified that the H3K4me3 and H3K27ac were highly enriched in the promoter regions of *Adrb2*, *Fshr*, *Tnfrsf19*, *Dtx4*, *Tcte2*, and *Ifit3b*. These results indicate that histone modifications were involved in the regulation of gene expression during ovarian GCs in response to DON toxicity.

NF-κB exists as homo-dimeric or hetero-dimeric complexes of p50 and p65 subunits bound to IκB and plays a key role in cellular responses to various stimuli such as stress, cytokines, free radicals, and bacterial or viral antigens [65]. Meanwhile, the mitogen-activated protein kinase (MAPK) signaling pathways, a family of serine/threonine protein kinases that mediate fundamental biological processes and cellular responses to external stress signals, are involved in a series of cell physiological activities such as inflammation, cell growth, development, differentiation, and apoptosis [66]. DON, as an environmental toxin or external stress signal, can induce GCs oxidative stress and mitochondria dysfunction as well as the release of oxygen free radicals. Therefore, the administration of the DON activated the NF-κB and MAPK signaling pathways, and the altered the gene regulation of the GCs caused an inflammatory response and other adverse effects.

In summary, our study revealed that DON has detrimental effects on murine ovarian GCs and systematically investigated the accessibility dynamics and transcriptional regulation of DON toxicity on ovarian GCs. We revealed the key regulatory elements involved in the toxicity of mycotoxins in reproductive cells and clarified the relationship between TF binding, chromatin-accessible regions, and gene regulation. These results give new insights into the molecular mechanisms underlying response of female reproductive cells to DON toxicity. However, future studies are needed to further elucidate the role of these alterations in the pathogenesis of reproductive diseases caused by DON toxicity and to develop biomarkers and drug targets. On the other hand, in vivo testing is needed to support our findings and the development of therapeutic regimens.

## Data Availability

All the sequenced data of the experimental samples are available in the NCBI Sequence Read Archive under the accession number PRJNA610700 (https://dataview.ncbi.nlm.nih.gov/object/PRJNA610700?reviewer=hq7orbfpspgqd17dcqj7d245nr).

## Supplementary Materials

The following are available online at https://www.mdpi.com/article/10.3390/cells10112818/s1, Figure S1: ATAC-seq data quality control metrics of fragment size distribution and sequencing read enrichment. Figure S2: The principal components analysis (PCA) plot of ATAC-seq samples, Table S1: Oligonucleotide sequences and size of primers, Table S2: List of primers used for ChIP-qPCR, Table S3: Summary statistics for ATAC-seq data, Table S4: List of differential accessible peaks, Table S5: The annotation of upregulated peaks. Table S6: The annotation of downregulated peaks, Table S7: Signaling pathway associated with downregulated chromatin accessibility, Table S8: Signaling pathway associated with upregulated chromatin accessibility, Table S9: GO analysis of biological process associated with downregulated chromatin-accessible regions, Table S10: GO analysis of biological process associated with upregulated chromatin-accessible regions, Table S11: Differentially expressed genes between DON-treated and control groups, Table S12: Summary statistics for RNA-seq data, Table S13: Signaling pathway associated with downregulated genes, Table S14: Signaling pathway associated with upregulated genes, Table S15: GO analysis of biological process associated with downregulated genes, Table S16: GO analysis of biological process associated with upregulated genes, Table S17: The genes identified by integrated analysis of ATAC-seq and RNA-seq.

## Abbreviations

*Adrb2*: adrenergic receptor, beta 2; *Dtx4*, deltex E3 ubiquitin ligase 4; *Fbxo27*: F-box protein 27; *Fshr*, follicle stimulating hormone receptor; *Nr3c2*: nuclear receptor subfamily 3 group C member 2; *Tcte2*, t-complex-associated testis expressed 2; *Tnfrsf19*, TNF receptor superfamily member 19; *Zfp930*, zinc finger protein 930; *Btbd11*, BTB domain containing 11; Ciita, class II major histocompatibility complex transactivator; *Cobll1*, Cobl-like 1; *Doc2b*, double C2, beta; *Galnt13*, polypeptide N-acetylgalactosaminyl transferase 13; *Iqgap2*, IQ motif containing GTPase activating protein 2; Jcad, junctional cadherin 5 associated; *Kcnf1*, potassium voltage-gated channel modifier subfamily F member 1; *Kcnh1*, potassium voltage-gated channel subfamily H member 1; *Mfrp*, membrane frizzled-related protein; *Ndufaf6*, NADH: ubiquinone oxidoreductase complex assembly factor 6; *Npc1l1*, NPC1-like intracellular cholesterol transporter 1; *Plb1*, phospholipase B1; *Ptafr*, platelet activating factor receptor; *Tnfrsf21*, TNF receptor superfamily member 21; *Cacna1a*, calcium voltage-gated channel subunit alpha1 a; *Camk1g*, calcium/calmodulin dependent protein kinase IG; *Chrdl1*, chordin-like 1; *Col3a1*, collagen type III alpha 1 chain; *Ifit3*, interferon induced protein with tetratricopeptide repeats 3; *Ifit3b*, interferon-induced protein with tetratricopeptide repeats 3B; *Rgs2*, regulator of G protein signaling 2; *Tmem45a*, transmembrane protein 45A; *Slc6a12*, solute carrier family 6 member 12; *1700016C15Rik*, RIKEN cDNA 1700016C15 gene; *S1pr1*, sphingosine-1-phosphate receptor 1; *Slc9b1*, solute carrier family 9 member B1; Nr5a2, Nuclear receptor subfamily 5, group A, member 2; SF-1, Steroidogenic factor 1; AP-1, activator protein-1.
