# Supplementary material for: Chromatin Accessibility and Transcriptomic Alterations in Murine Ovarian Granulosa Cells upon Deoxynivalenol Exposure
Source: Cells. 2021 Oct 20;10(11):2818. doi: 10.3390/cells10112818 (PMC8616273; doi:10.3390/cells10112818)
Supplement: Supplementary file 1 [file cells-10-02818-s001.zip › cells-1374880/cells-1374880-supplement.pdf]

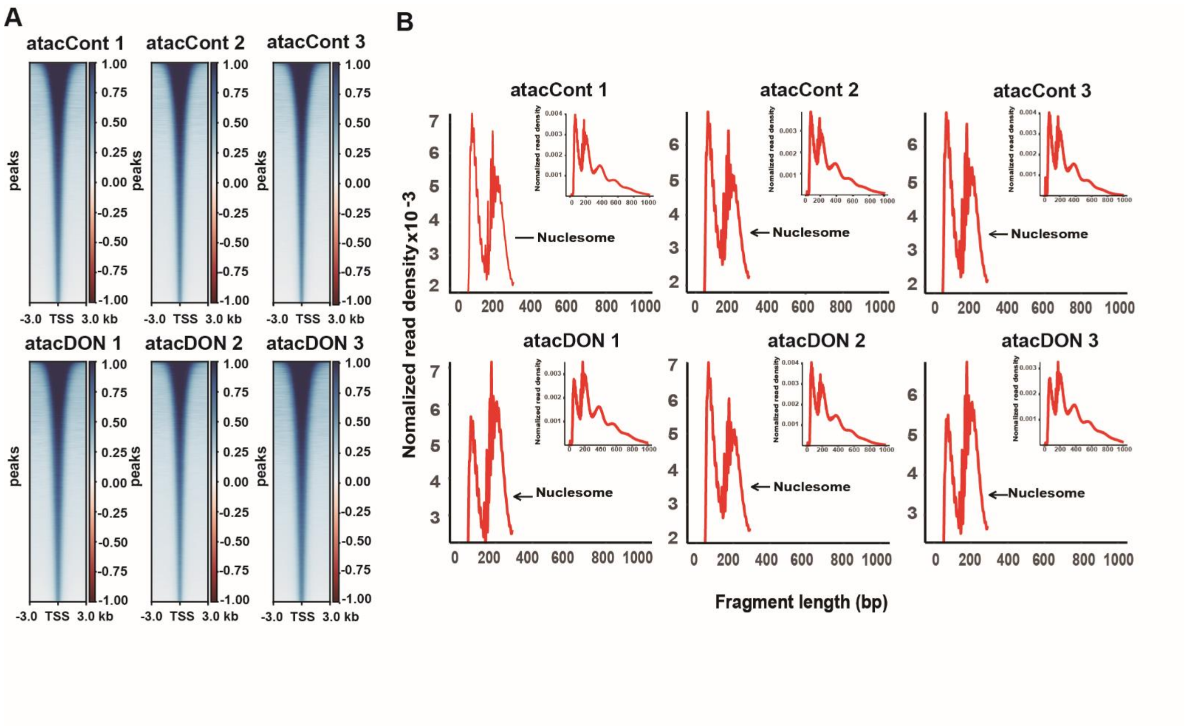

Figure S1. ATAC-seq data quality control metrics of fragment size distribution and sequencing read enrichment.

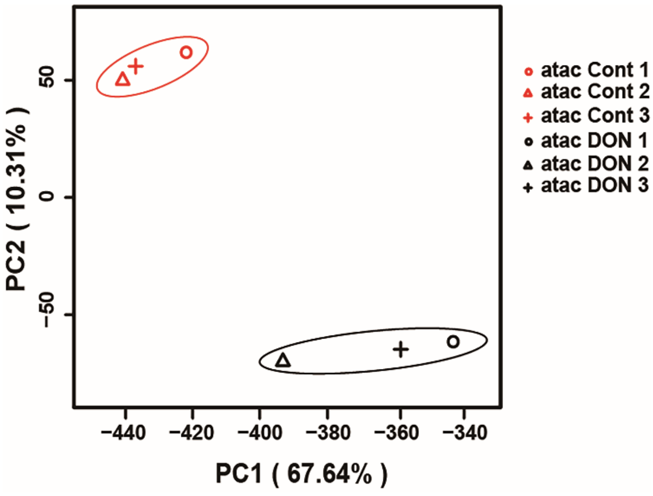

Figure S2. The principal components analysis (PCA) plot of ATAC-seq samples.
