# Supplementary figures and images for: Chromatin Accessibility and Transcriptomic Alterations in Murine Ovarian Granulosa Cells upon Deoxynivalenol Exposure (part 2 of 2)
Source: Cells. 2021 Oct 20;10(11):2818. doi: 10.3390/cells10112818 (PMC8616273; doi:10.3390/cells10112818)

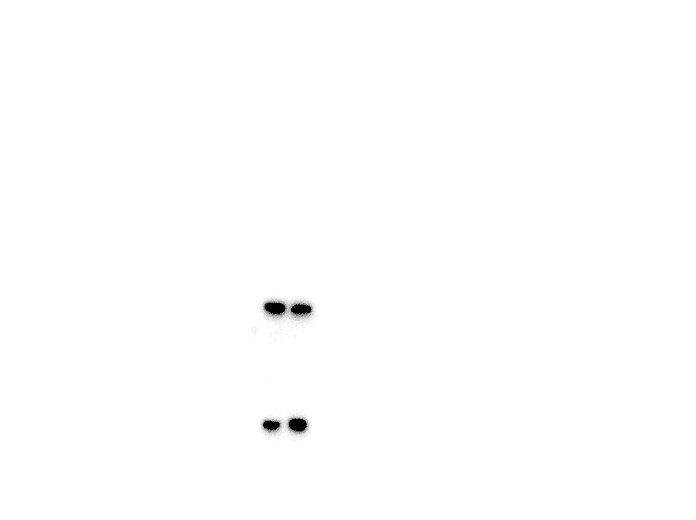

Supplement: Supplementary file 1 [file cells-10-02818-s001.zip › cells-1374880/The full bolt images for the Western Blot/p-P38/p-P38(down) and HSP90(up)-2.tif]

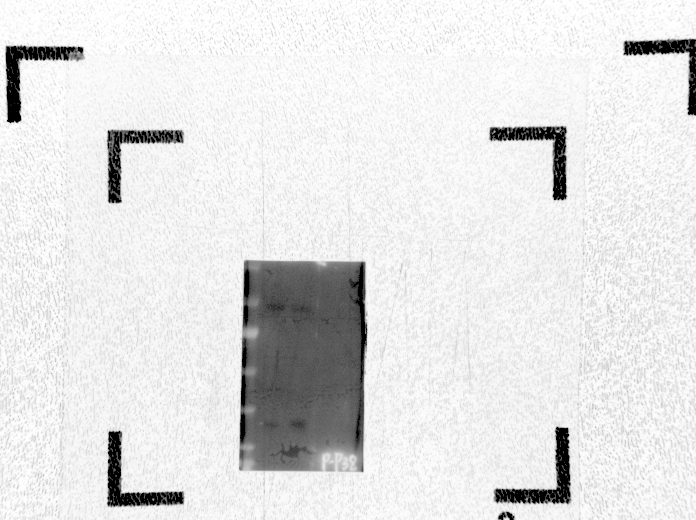

Supplement: Supplementary file 1 [file cells-10-02818-s001.zip › cells-1374880/The full bolt images for the Western Blot/p-P38/p-P38(down) and HSP90(up)-3.tif]

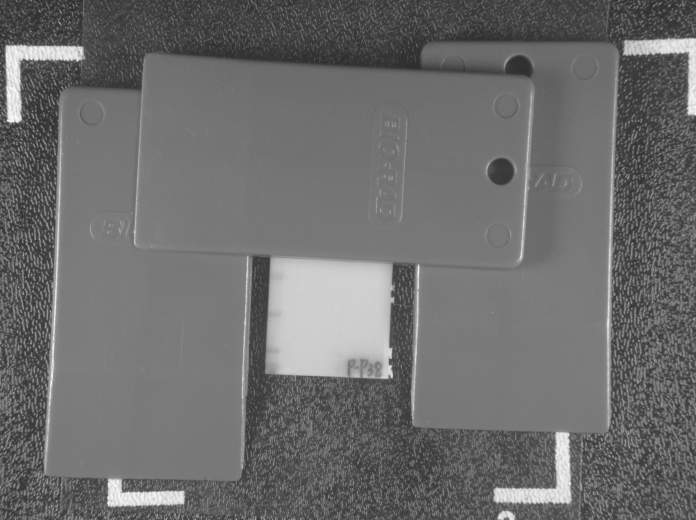

Supplement: Supplementary file 1 [file cells-10-02818-s001.zip › cells-1374880/The full bolt images for the Western Blot/p-P38/p-P38-1.tif]

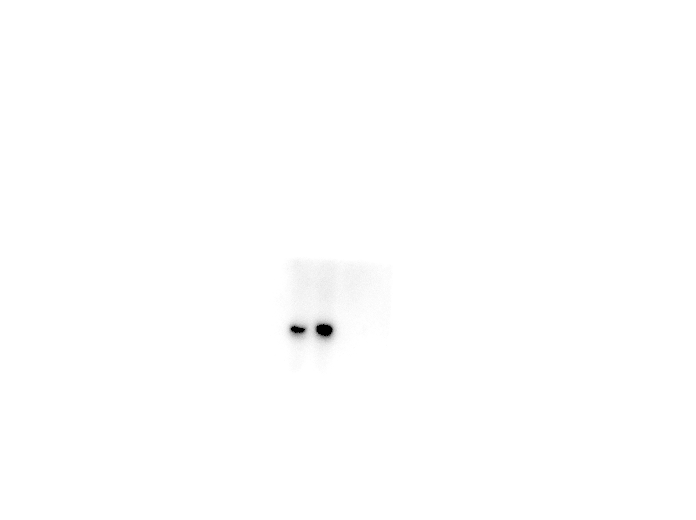

Supplement: Supplementary file 1 [file cells-10-02818-s001.zip › cells-1374880/The full bolt images for the Western Blot/p-P38/p-P38-2.tif]

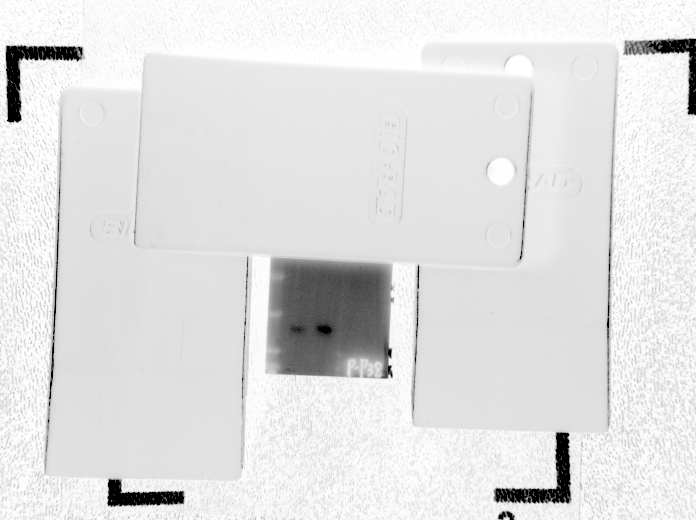

Supplement: Supplementary file 1 [file cells-10-02818-s001.zip › cells-1374880/The full bolt images for the Western Blot/p-P38/p-P38-3.tif]

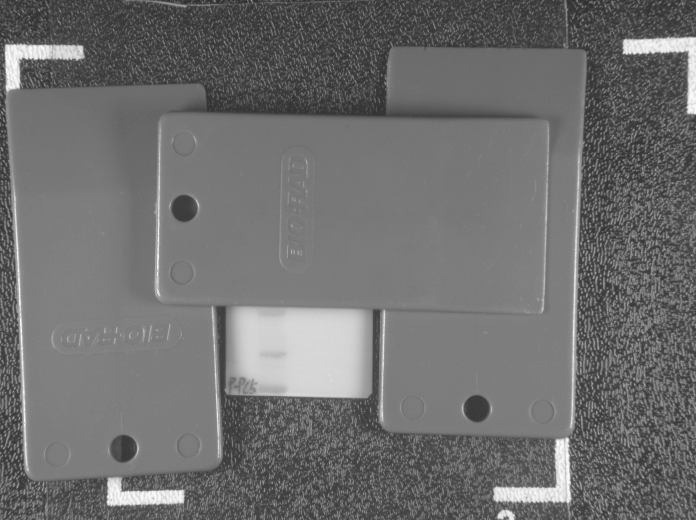

Supplement: Supplementary file 1 [file cells-10-02818-s001.zip › cells-1374880/The full bolt images for the Western Blot/p-p65/GAPDH-1.tif]

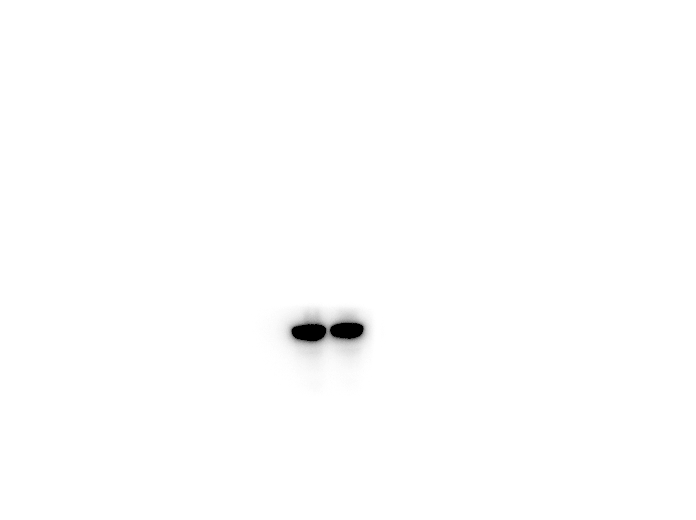

Supplement: Supplementary file 1 [file cells-10-02818-s001.zip › cells-1374880/The full bolt images for the Western Blot/p-p65/GAPDH-2.tif]

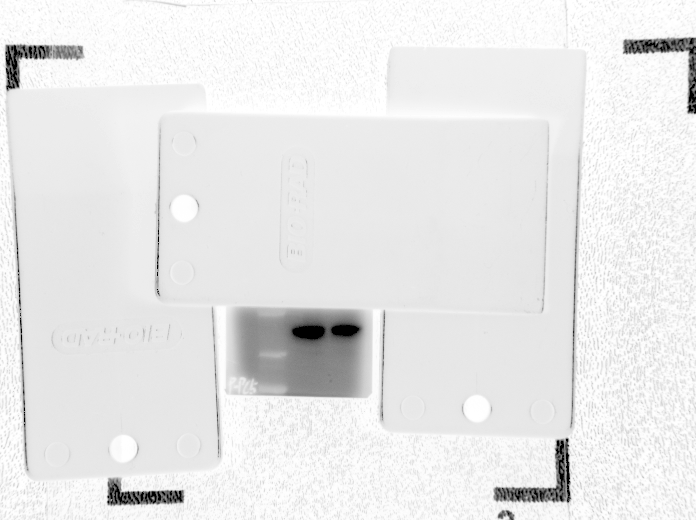

Supplement: Supplementary file 1 [file cells-10-02818-s001.zip › cells-1374880/The full bolt images for the Western Blot/p-p65/GAPDH-3.tif]

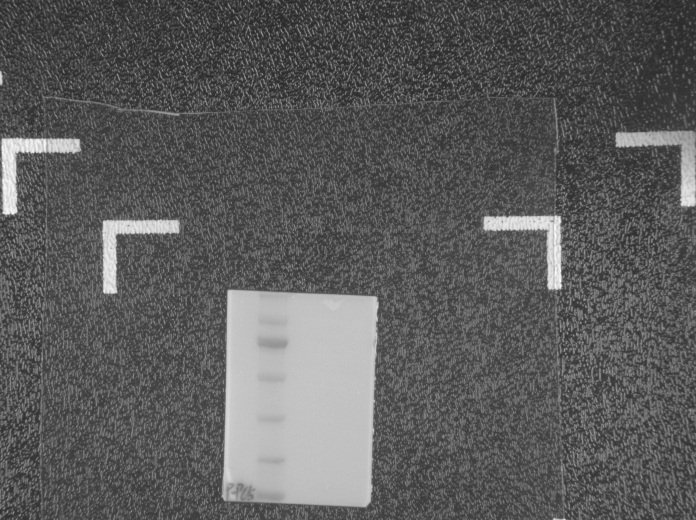

Supplement: Supplementary file 1 [file cells-10-02818-s001.zip › cells-1374880/The full bolt images for the Western Blot/p-p65/p-P65(up) and GAPDH(down)-1.tif]

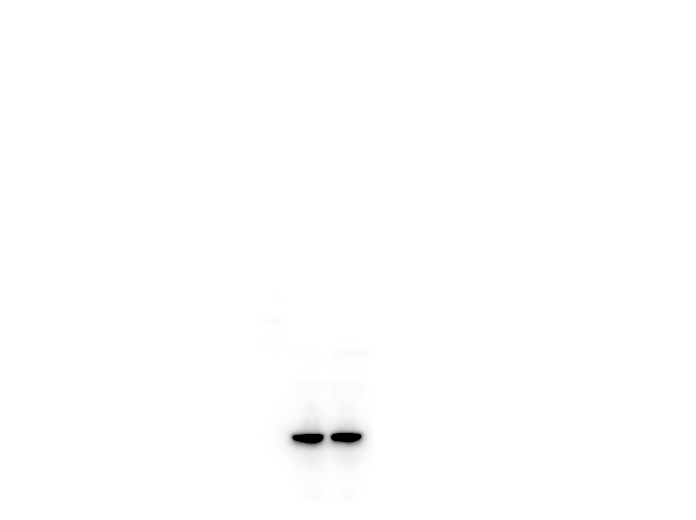

Supplement: Supplementary file 1 [file cells-10-02818-s001.zip › cells-1374880/The full bolt images for the Western Blot/p-p65/p-P65(up) and GAPDH(down)-2.tif]

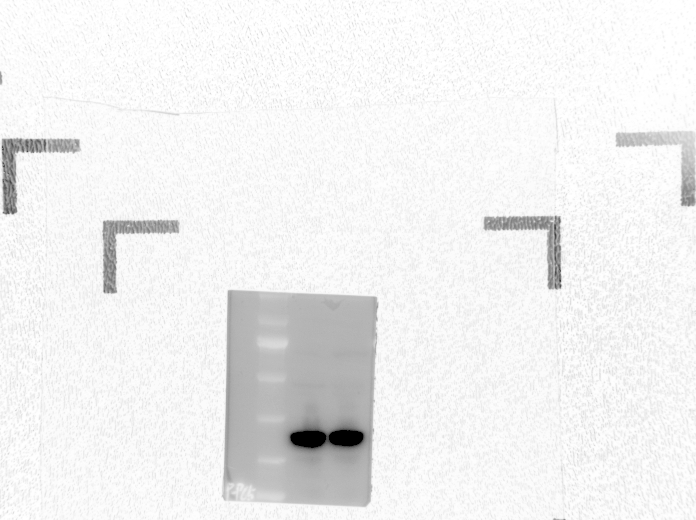

Supplement: Supplementary file 1 [file cells-10-02818-s001.zip › cells-1374880/The full bolt images for the Western Blot/p-p65/p-P65(up) and GAPDH(down)-3.tif]

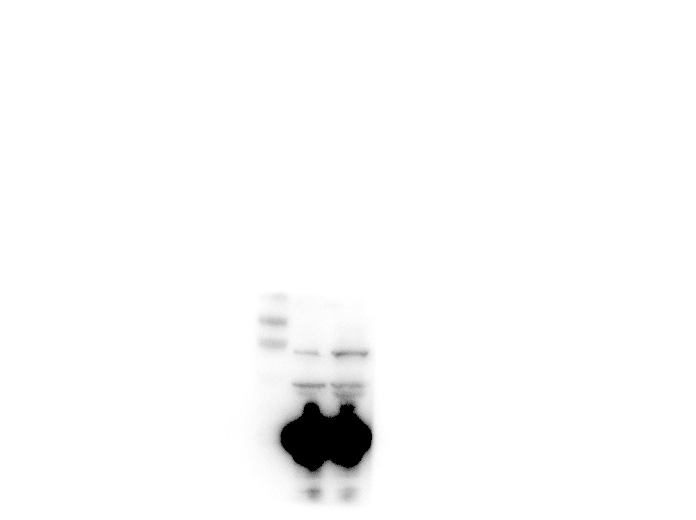

Supplement: Supplementary file 1 [file cells-10-02818-s001.zip › cells-1374880/The full bolt images for the Western Blot/p-p65/p-P65(up) and GAPDH(down)-4.tif]

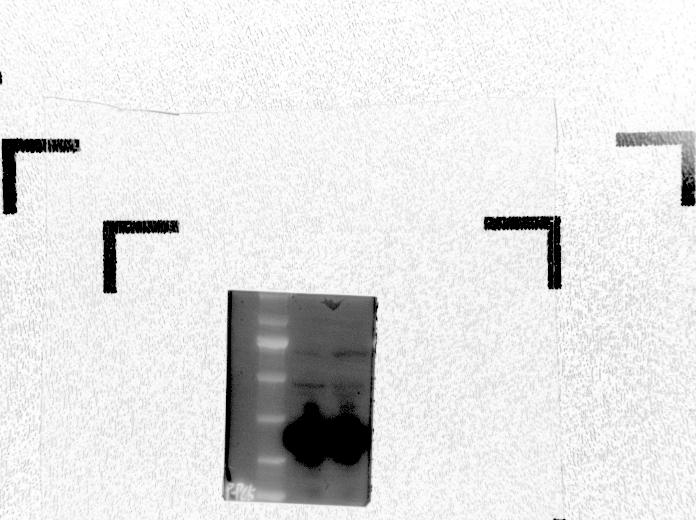

Supplement: Supplementary file 1 [file cells-10-02818-s001.zip › cells-1374880/The full bolt images for the Western Blot/p-p65/p-P65(up) and GAPDH(down)-5.tif]

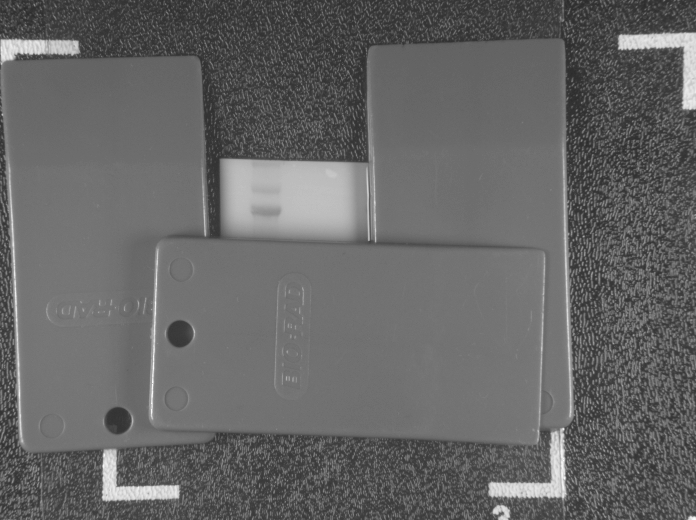

Supplement: Supplementary file 1 [file cells-10-02818-s001.zip › cells-1374880/The full bolt images for the Western Blot/p-p65/p-P65-1.tif]

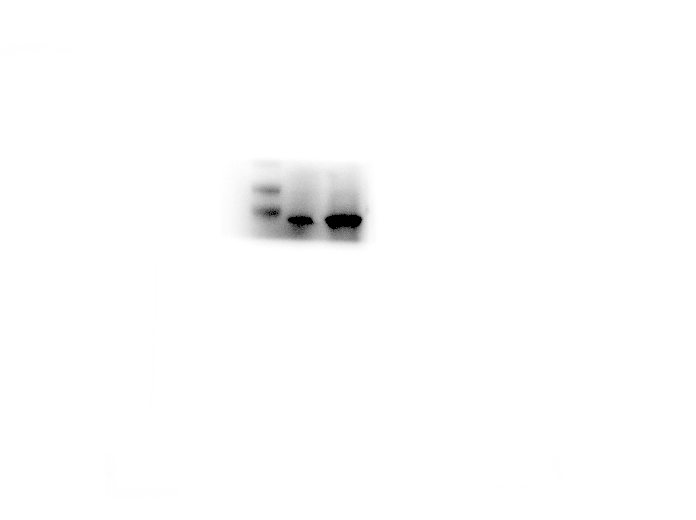

Supplement: Supplementary file 1 [file cells-10-02818-s001.zip › cells-1374880/The full bolt images for the Western Blot/p-p65/p-P65-2.tif]

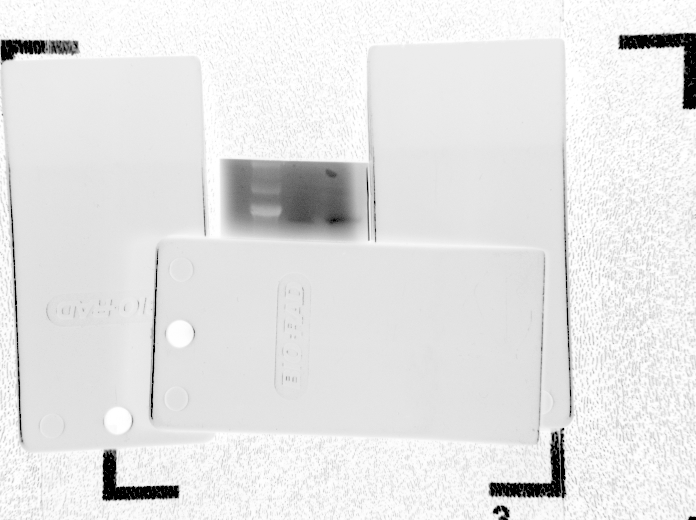

Supplement: Supplementary file 1 [file cells-10-02818-s001.zip › cells-1374880/The full bolt images for the Western Blot/p-p65/p-P65-3.tif]

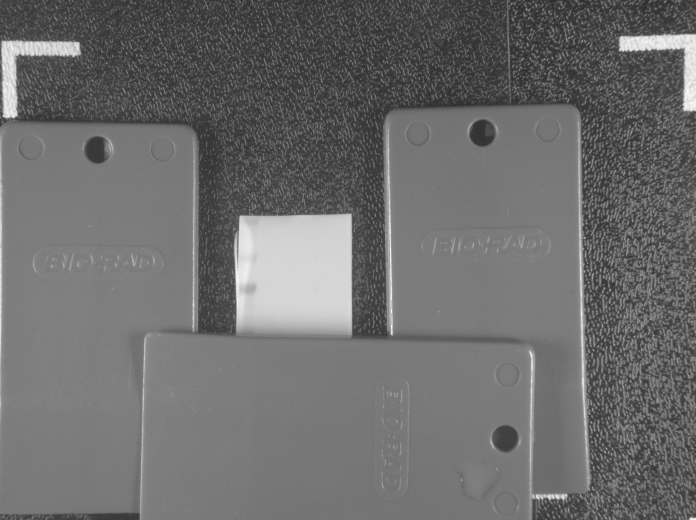

Supplement: Supplementary file 1 [file cells-10-02818-s001.zip › cells-1374880/The full bolt images for the Western Blot/P38/HSP90-1.tif]

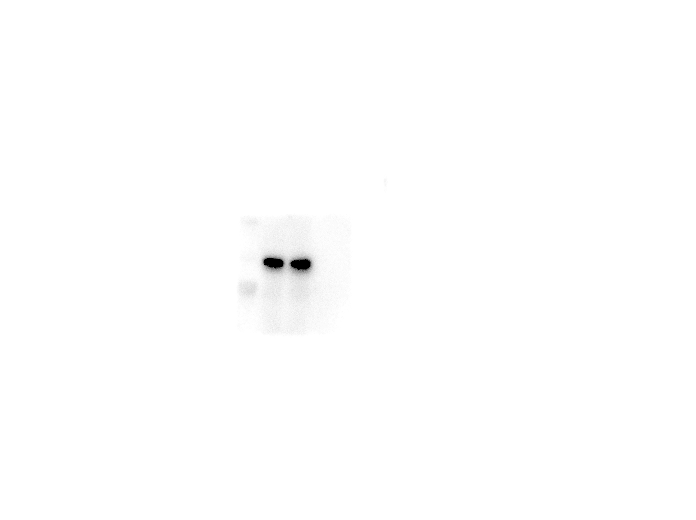

Supplement: Supplementary file 1 [file cells-10-02818-s001.zip › cells-1374880/The full bolt images for the Western Blot/P38/HSP90-2.tif]

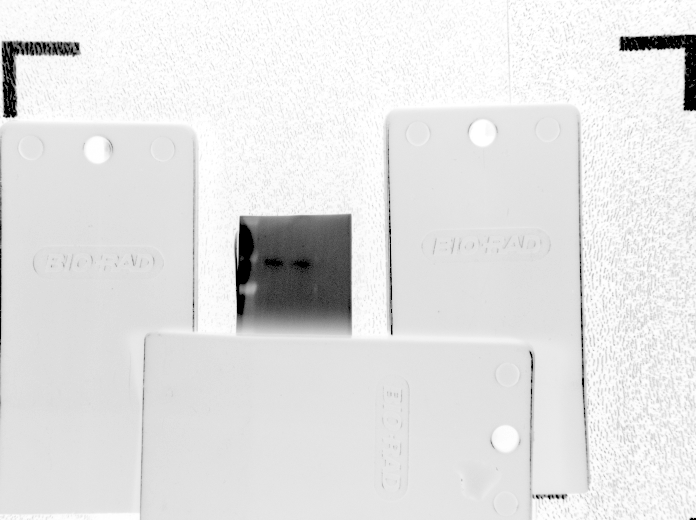

Supplement: Supplementary file 1 [file cells-10-02818-s001.zip › cells-1374880/The full bolt images for the Western Blot/P38/HSP90-3.tif]

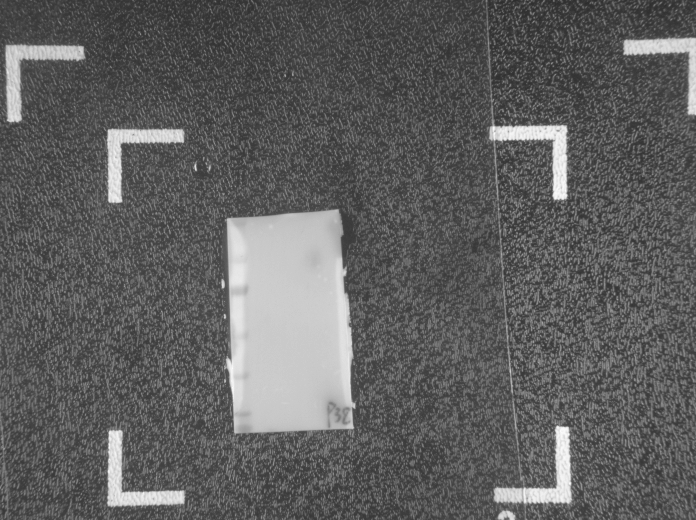

Supplement: Supplementary file 1 [file cells-10-02818-s001.zip › cells-1374880/The full bolt images for the Western Blot/P38/P38(down) and HSP90 (up)-1.tif]

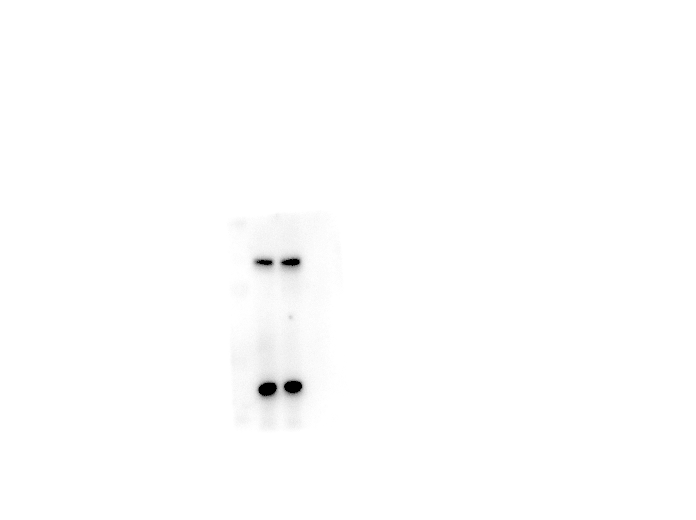

Supplement: Supplementary file 1 [file cells-10-02818-s001.zip › cells-1374880/The full bolt images for the Western Blot/P38/P38(down) and HSP90 (up)-2.tif]

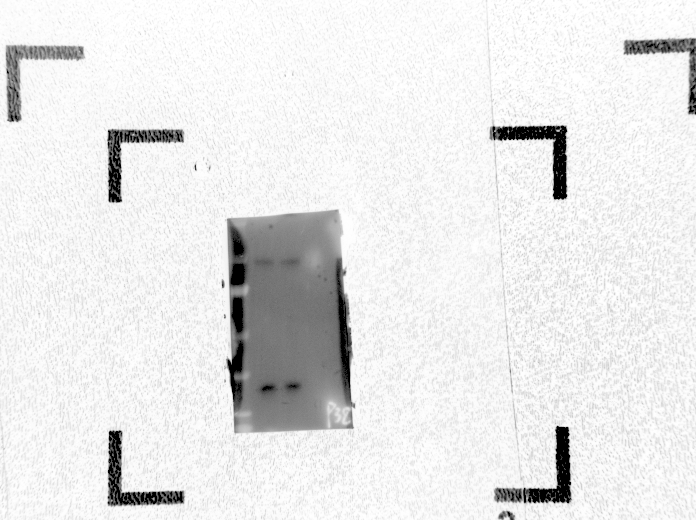

Supplement: Supplementary file 1 [file cells-10-02818-s001.zip › cells-1374880/The full bolt images for the Western Blot/P38/P38(down) and HSP90 (up)-3.tif]

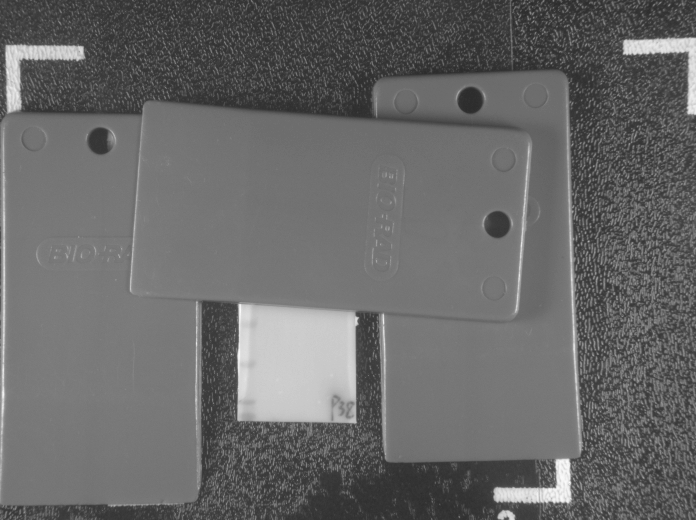

Supplement: Supplementary file 1 [file cells-10-02818-s001.zip › cells-1374880/The full bolt images for the Western Blot/P38/P38-1.tif]

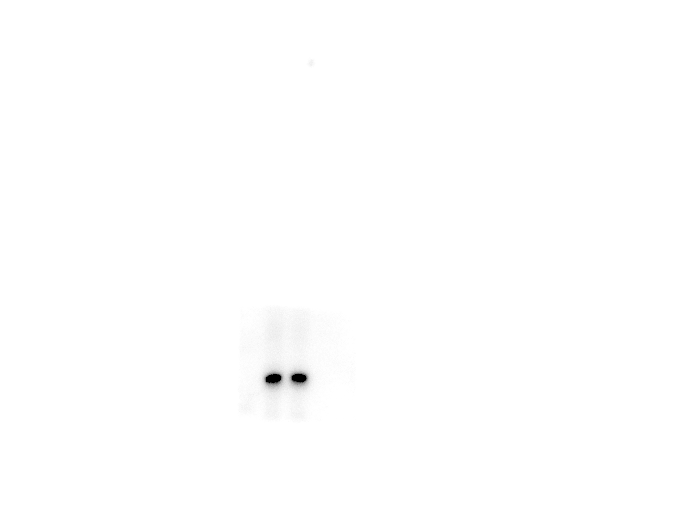

Supplement: Supplementary file 1 [file cells-10-02818-s001.zip › cells-1374880/The full bolt images for the Western Blot/P38/P38-2.tif]

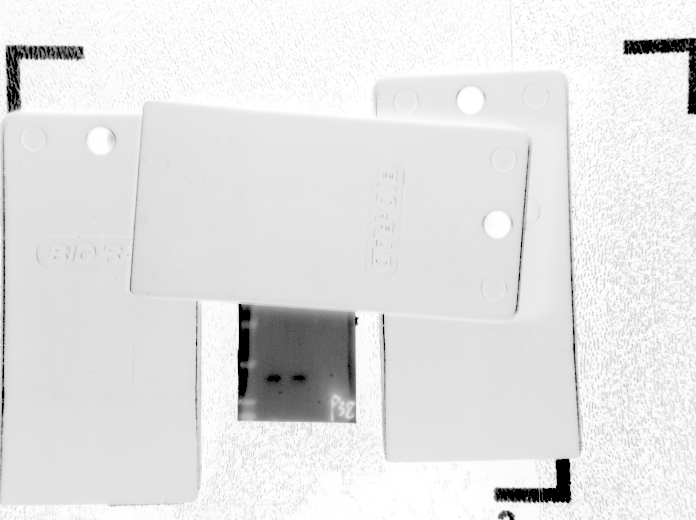

Supplement: Supplementary file 1 [file cells-10-02818-s001.zip › cells-1374880/The full bolt images for the Western Blot/P38/P38-3.tif]

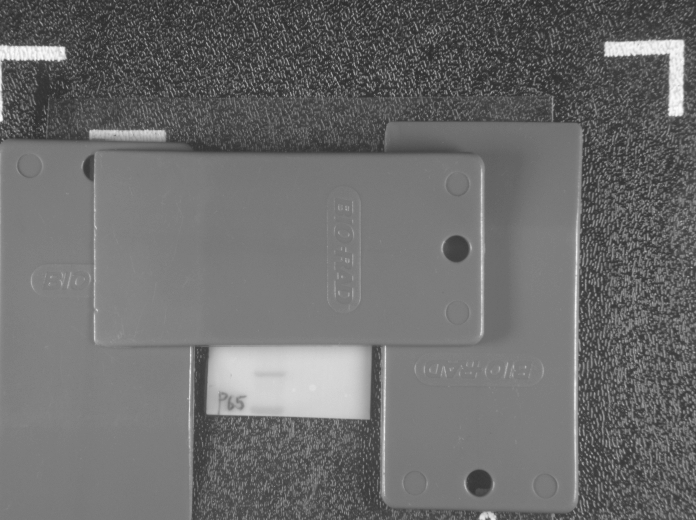

Supplement: Supplementary file 1 [file cells-10-02818-s001.zip › cells-1374880/The full bolt images for the Western Blot/P65/GAPDH-1.tif]

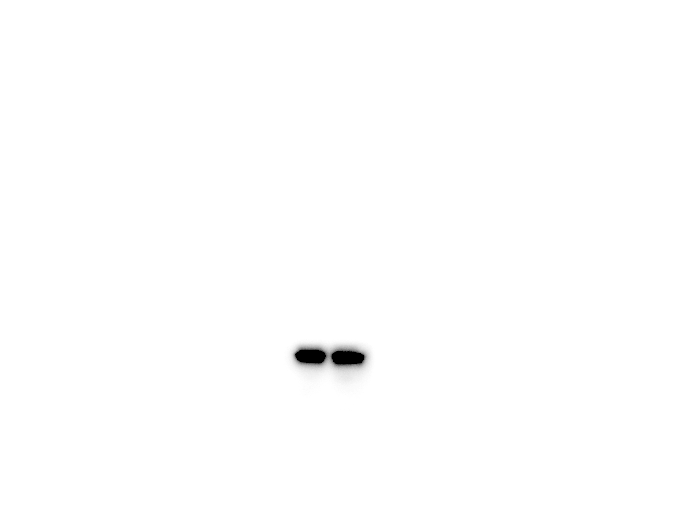

Supplement: Supplementary file 1 [file cells-10-02818-s001.zip › cells-1374880/The full bolt images for the Western Blot/P65/GAPDH-2.tif]

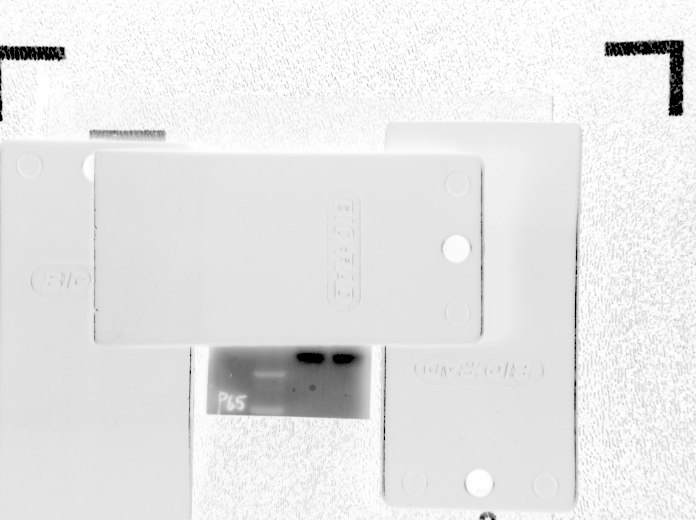

Supplement: Supplementary file 1 [file cells-10-02818-s001.zip › cells-1374880/The full bolt images for the Western Blot/P65/GAPDH-3.tif]

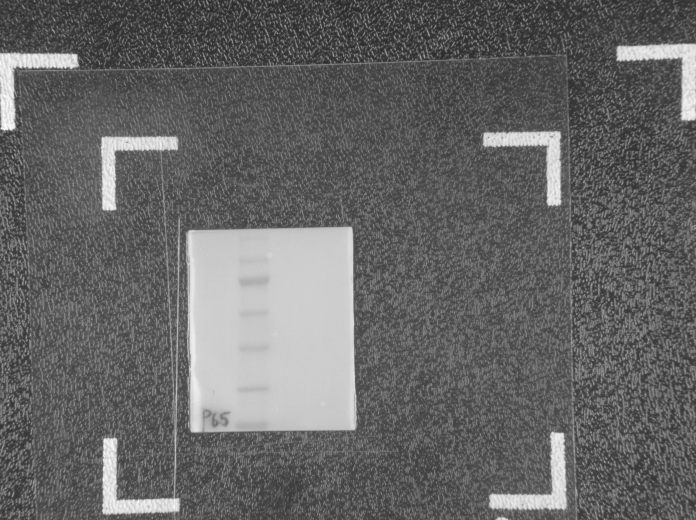

Supplement: Supplementary file 1 [file cells-10-02818-s001.zip › cells-1374880/The full bolt images for the Western Blot/P65/P65(up) and GAPDH(down)-1.tif]

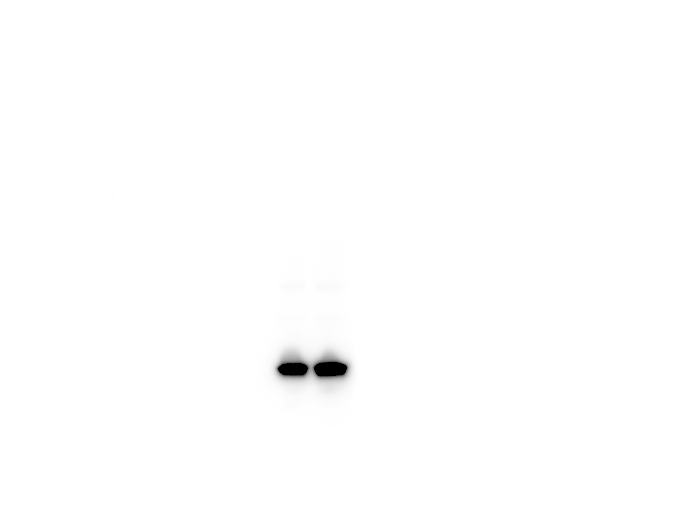

Supplement: Supplementary file 1 [file cells-10-02818-s001.zip › cells-1374880/The full bolt images for the Western Blot/P65/P65(up) and GAPDH(down)-2.tif]

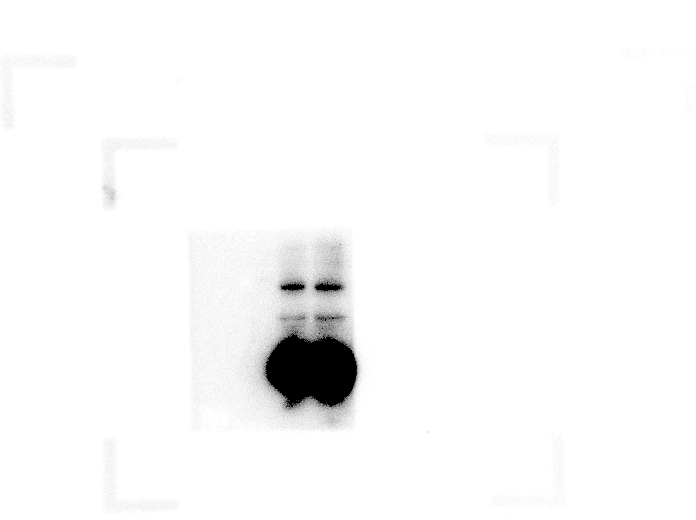

Supplement: Supplementary file 1 [file cells-10-02818-s001.zip › cells-1374880/The full bolt images for the Western Blot/P65/P65(up) and GAPDH(down)-3.tif]

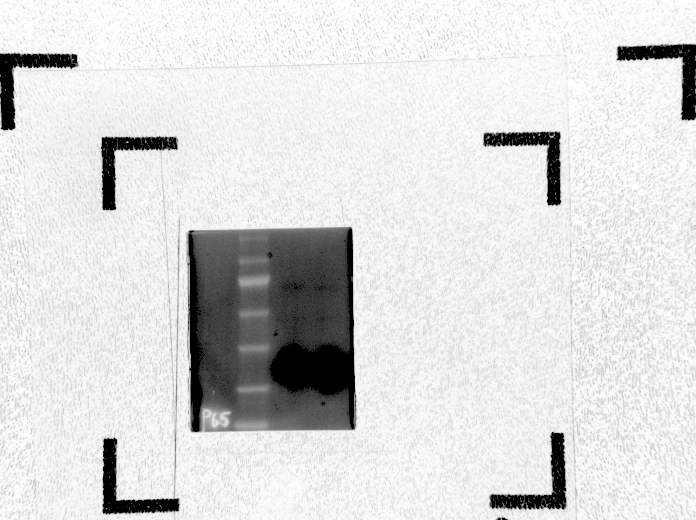

Supplement: Supplementary file 1 [file cells-10-02818-s001.zip › cells-1374880/The full bolt images for the Western Blot/P65/P65(up) and GAPDH(down)-4.tif]

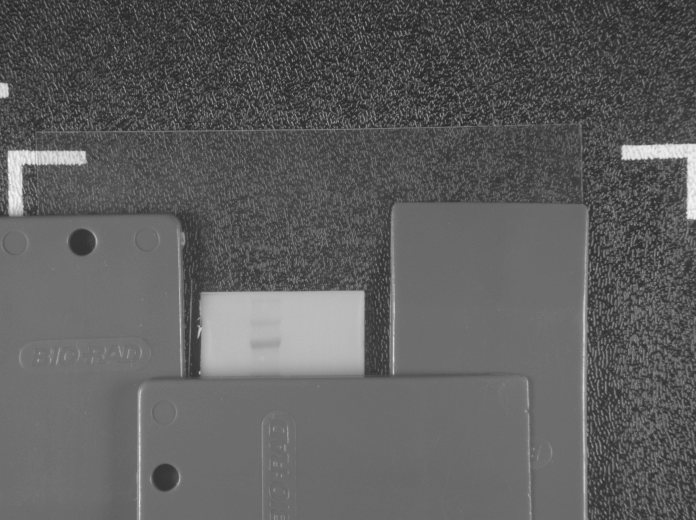

Supplement: Supplementary file 1 [file cells-10-02818-s001.zip › cells-1374880/The full bolt images for the Western Blot/P65/P65-1.tif]

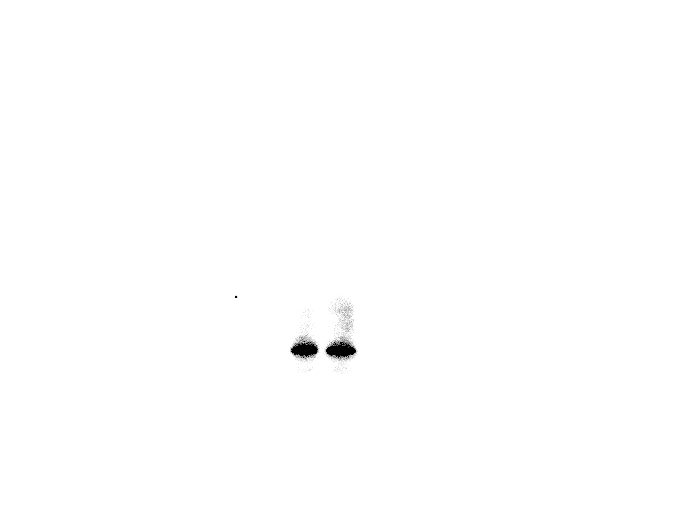

Supplement: Supplementary file 1 [file cells-10-02818-s001.zip › cells-1374880/The full bolt images for the Western Blot/P65/P65-2.tif]

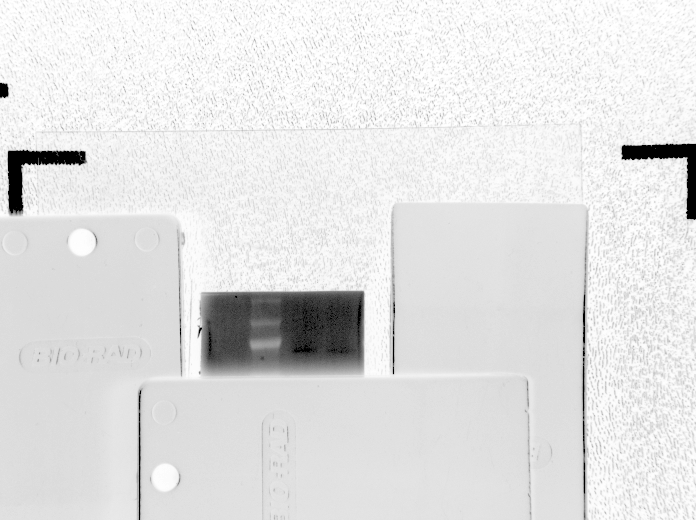

Supplement: Supplementary file 1 [file cells-10-02818-s001.zip › cells-1374880/The full bolt images for the Western Blot/P65/P65-3.tif]

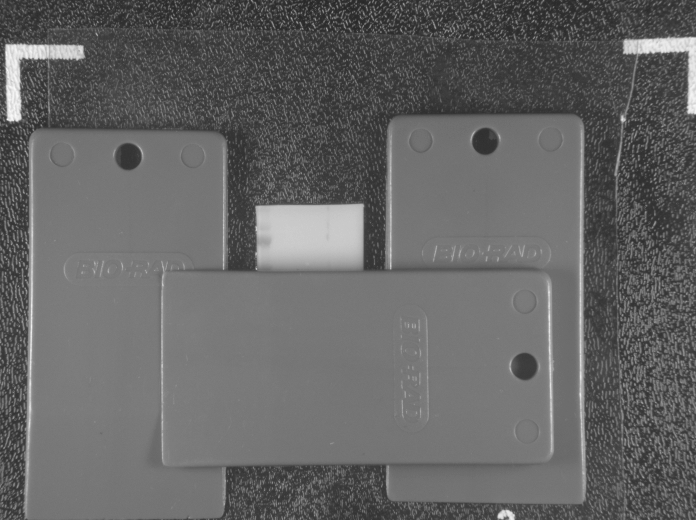

Supplement: Supplementary file 1 [file cells-10-02818-s001.zip › cells-1374880/The full bolt images for the Western Blot/PCNA/HSP90-1.tif]

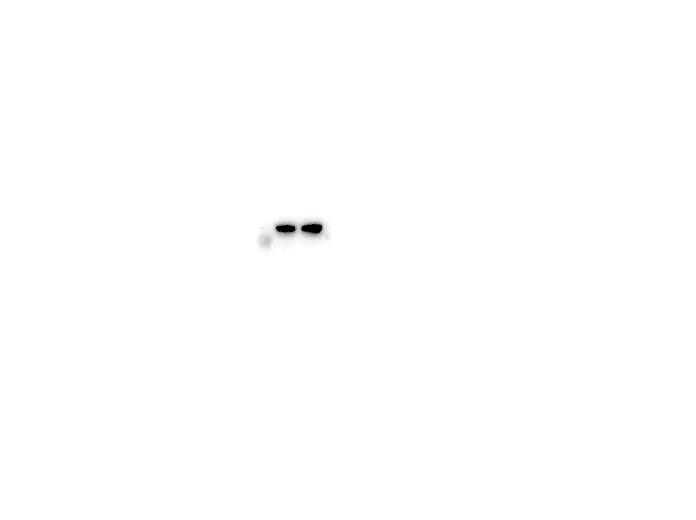

Supplement: Supplementary file 1 [file cells-10-02818-s001.zip › cells-1374880/The full bolt images for the Western Blot/PCNA/HSP90-2.tif]

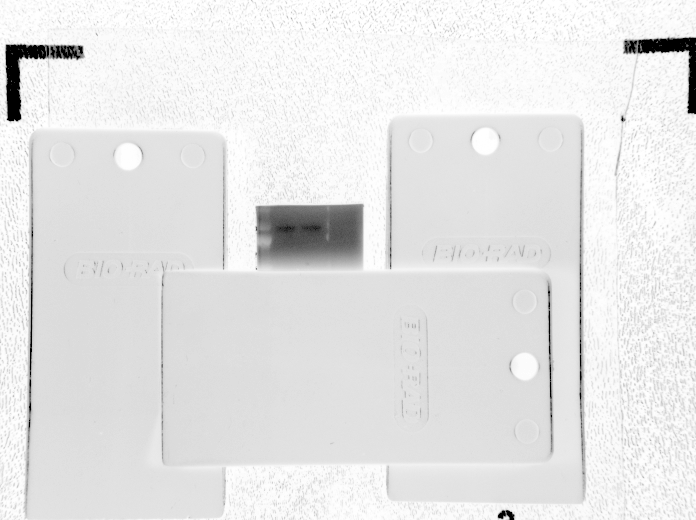

Supplement: Supplementary file 1 [file cells-10-02818-s001.zip › cells-1374880/The full bolt images for the Western Blot/PCNA/HSP90-3.tif]

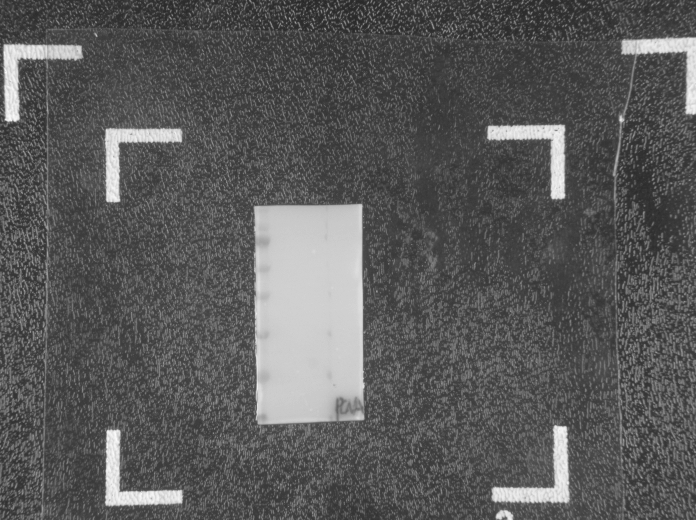

Supplement: Supplementary file 1 [file cells-10-02818-s001.zip › cells-1374880/The full bolt images for the Western Blot/PCNA/PCNA(down) and HSP90(up)-1.tif]

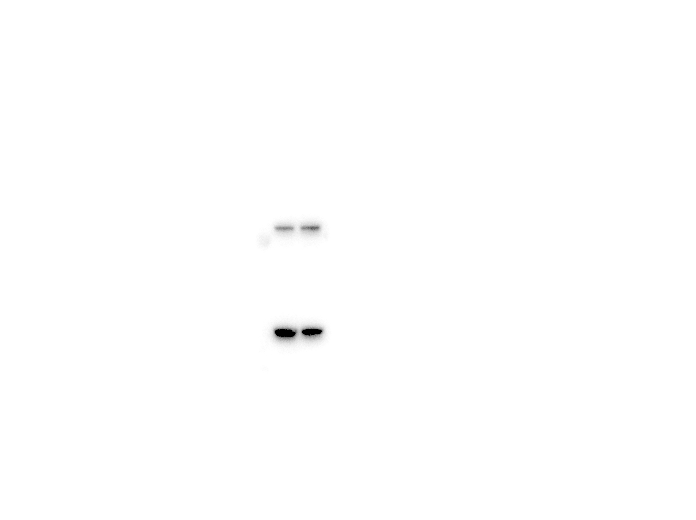

Supplement: Supplementary file 1 [file cells-10-02818-s001.zip › cells-1374880/The full bolt images for the Western Blot/PCNA/PCNA(down) and HSP90(up)-2.tif]

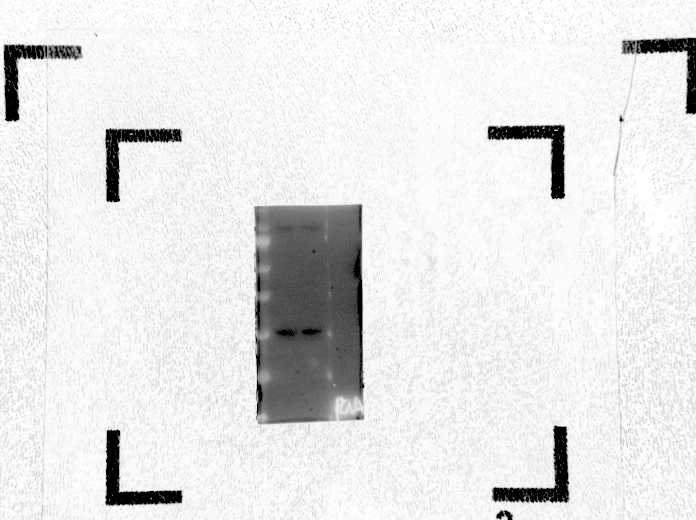

Supplement: Supplementary file 1 [file cells-10-02818-s001.zip › cells-1374880/The full bolt images for the Western Blot/PCNA/PCNA(down) and HSP90(up)-3.tif]

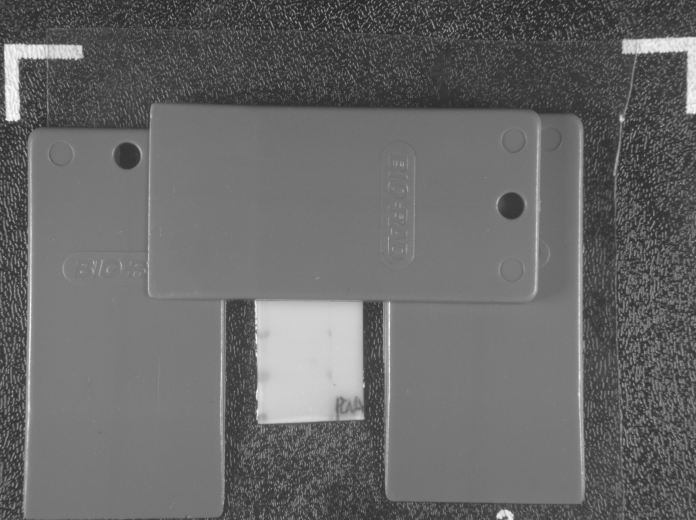

Supplement: Supplementary file 1 [file cells-10-02818-s001.zip › cells-1374880/The full bolt images for the Western Blot/PCNA/PCNA-1.tif]

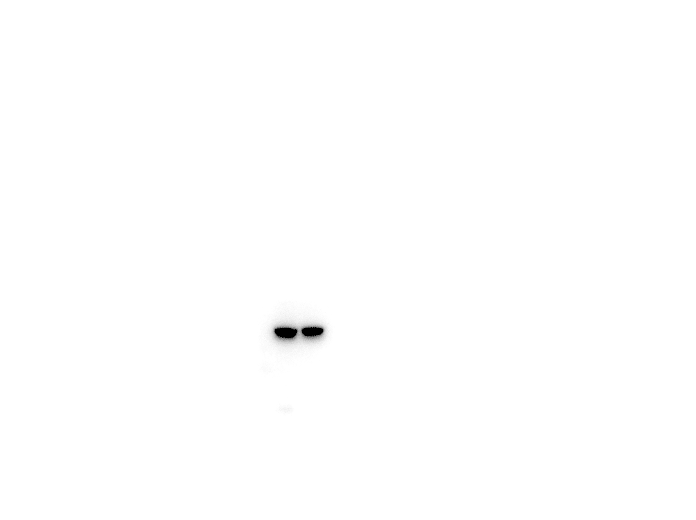

Supplement: Supplementary file 1 [file cells-10-02818-s001.zip › cells-1374880/The full bolt images for the Western Blot/PCNA/PCNA-2.tif]

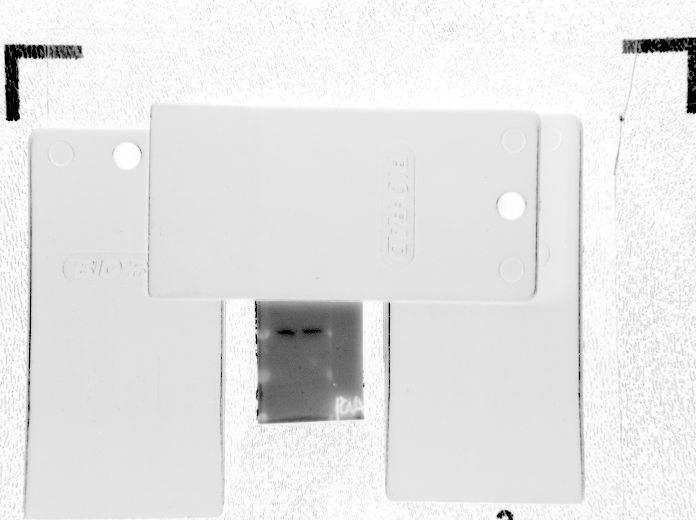

Supplement: Supplementary file 1 [file cells-10-02818-s001.zip › cells-1374880/The full bolt images for the Western Blot/PCNA/PCNA-3.tif]
