# Supplementary figures and images for: Chromatin Accessibility and Transcriptomic Alterations in Murine Ovarian Granulosa Cells upon Deoxynivalenol Exposure (part 1 of 2)
Source: Cells. 2021 Oct 20;10(11):2818. doi: 10.3390/cells10112818 (PMC8616273; doi:10.3390/cells10112818)

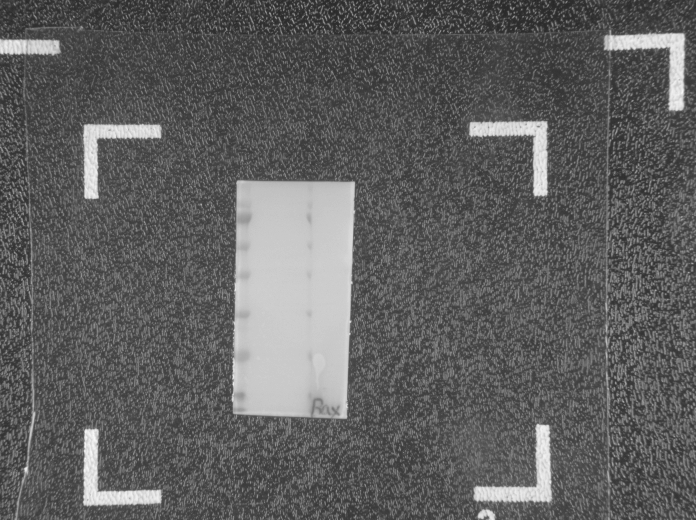

Supplement: Supplementary file 1 [file cells-10-02818-s001.zip › cells-1374880/The full bolt images for the Western Blot/Bax/Bax and GAPDH-1.tif]

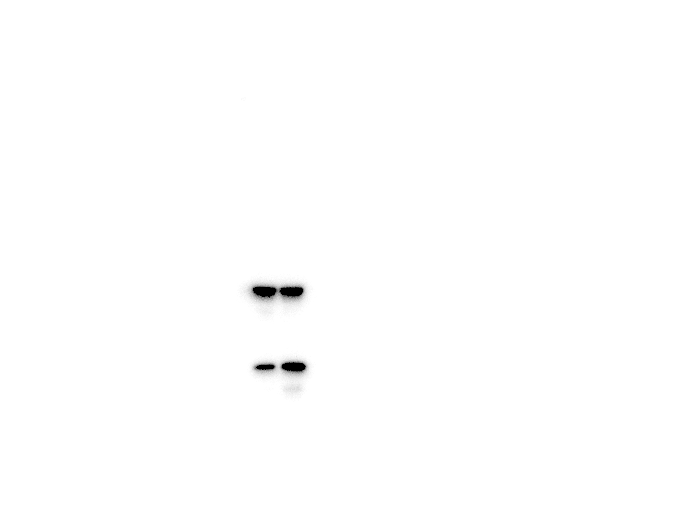

Supplement: Supplementary file 1 [file cells-10-02818-s001.zip › cells-1374880/The full bolt images for the Western Blot/Bax/Bax(down) and GAPDH(up)-2.tif]

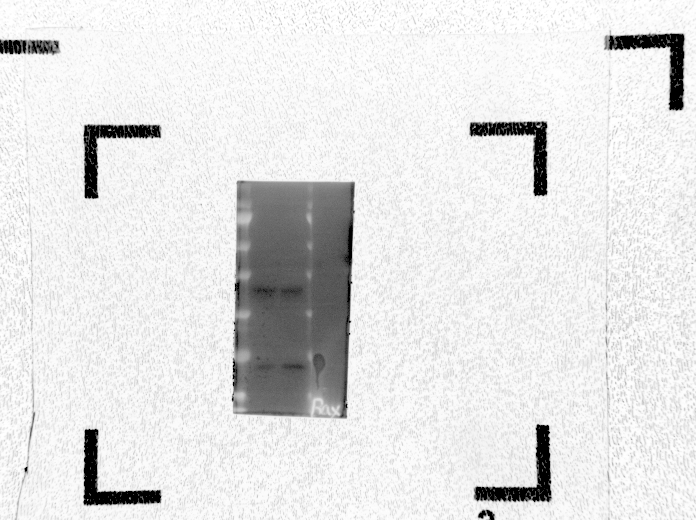

Supplement: Supplementary file 1 [file cells-10-02818-s001.zip › cells-1374880/The full bolt images for the Western Blot/Bax/Bax(down) and GAPDH(up)-3.tif]

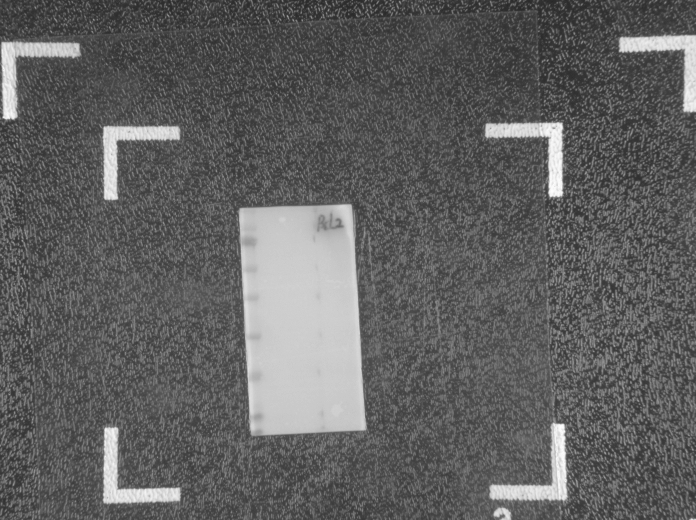

Supplement: Supplementary file 1 [file cells-10-02818-s001.zip › cells-1374880/The full bolt images for the Western Blot/Bcl-2/Bcl2(down) and GAPDH(up)-1.tif]

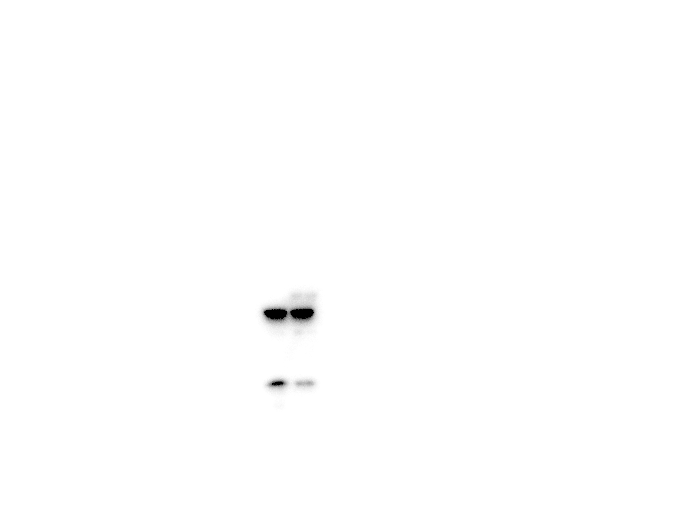

Supplement: Supplementary file 1 [file cells-10-02818-s001.zip › cells-1374880/The full bolt images for the Western Blot/Bcl-2/Bcl2(down) and GAPDH(up)-2.tif]

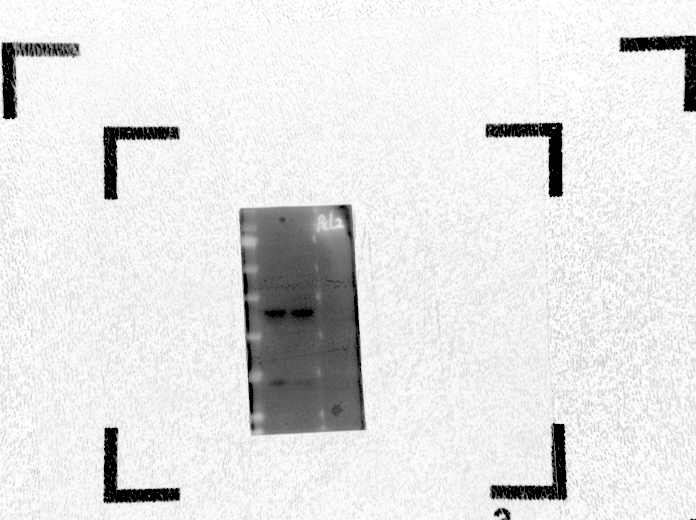

Supplement: Supplementary file 1 [file cells-10-02818-s001.zip › cells-1374880/The full bolt images for the Western Blot/Bcl-2/Bcl2(down) and GAPDH(up)-3.tif]

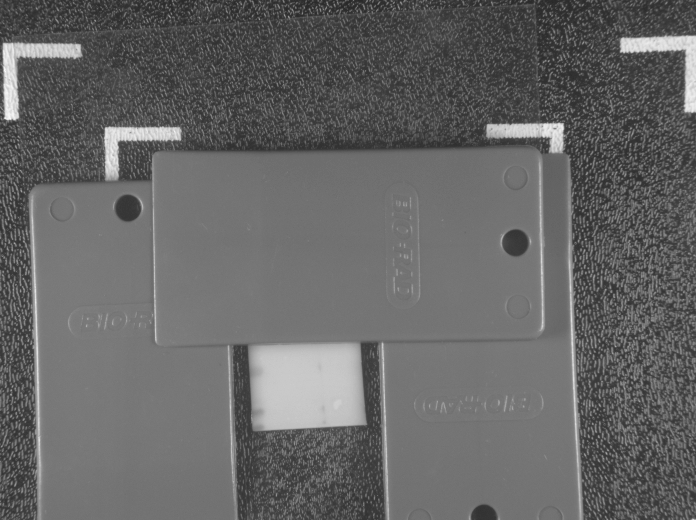

Supplement: Supplementary file 1 [file cells-10-02818-s001.zip › cells-1374880/The full bolt images for the Western Blot/Bcl-2/Bcl2-1.tif]

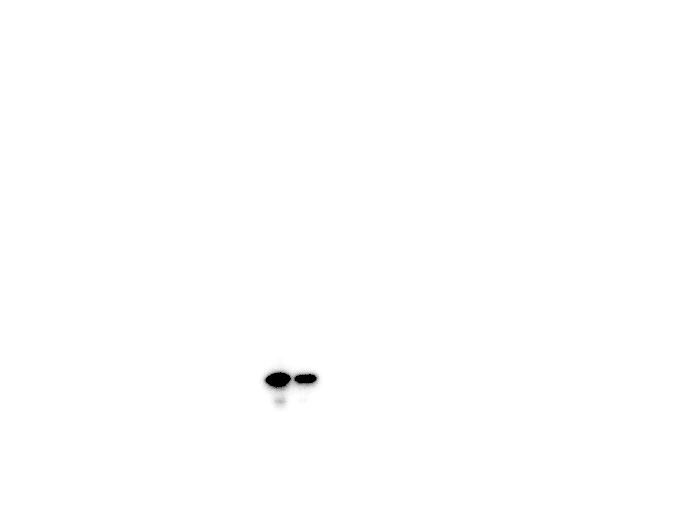

Supplement: Supplementary file 1 [file cells-10-02818-s001.zip › cells-1374880/The full bolt images for the Western Blot/Bcl-2/Bcl2-2.tif]

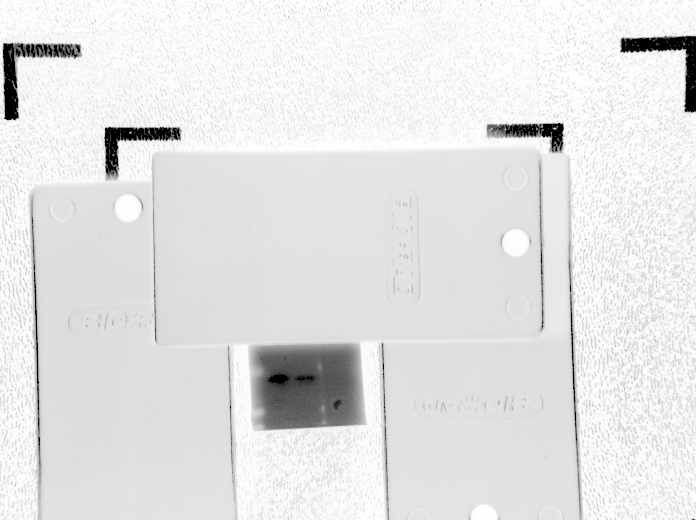

Supplement: Supplementary file 1 [file cells-10-02818-s001.zip › cells-1374880/The full bolt images for the Western Blot/Bcl-2/Bcl2-3.tif]

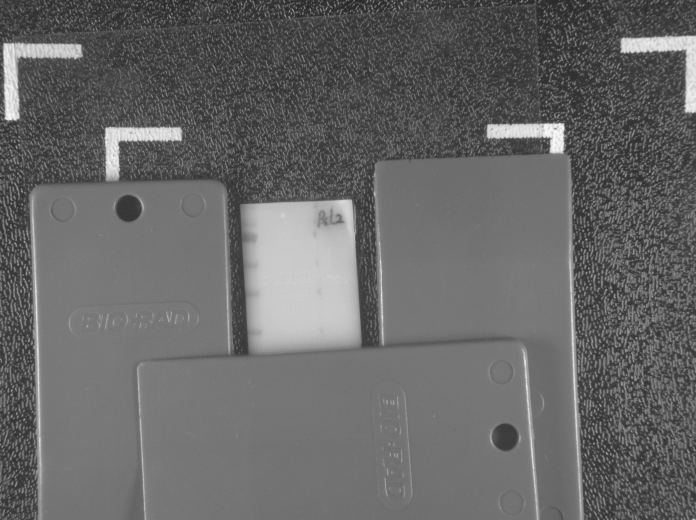

Supplement: Supplementary file 1 [file cells-10-02818-s001.zip › cells-1374880/The full bolt images for the Western Blot/Bcl-2/GAPDG-1.tif]

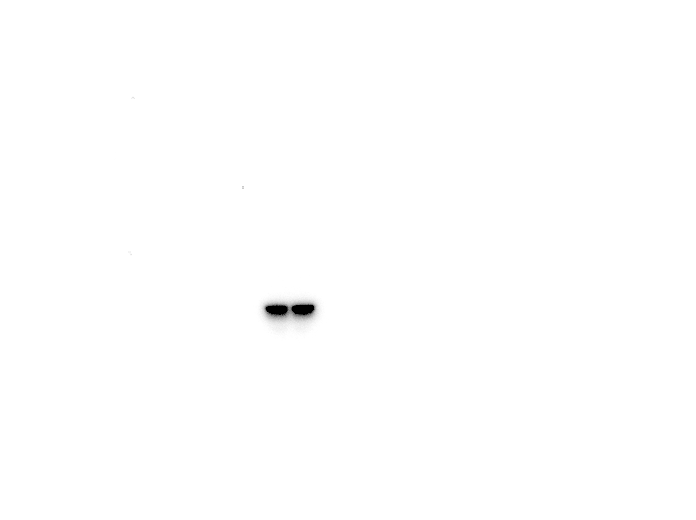

Supplement: Supplementary file 1 [file cells-10-02818-s001.zip › cells-1374880/The full bolt images for the Western Blot/Bcl-2/GAPDG-2.tif]

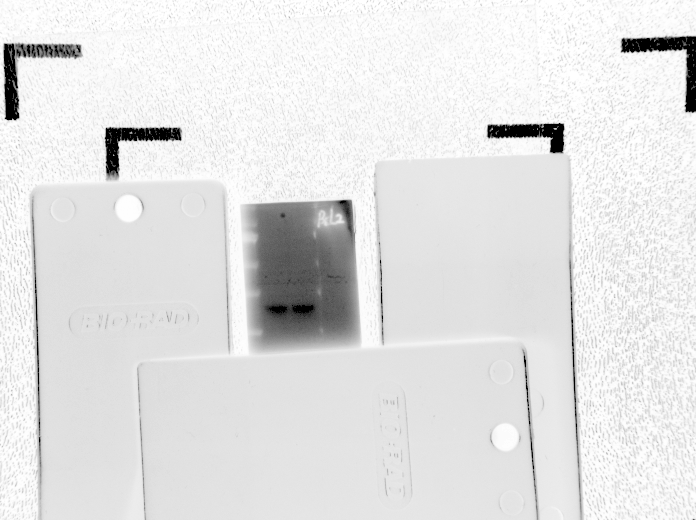

Supplement: Supplementary file 1 [file cells-10-02818-s001.zip › cells-1374880/The full bolt images for the Western Blot/Bcl-2/GAPDG-3.tif]

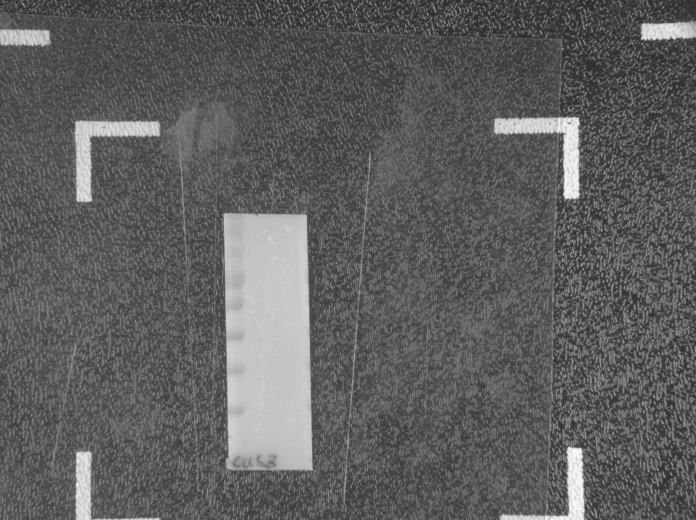

Supplement: Supplementary file 1 [file cells-10-02818-s001.zip › cells-1374880/The full bolt images for the Western Blot/caspase3/Caspse3(down) and HSP90(up)-1.tif]

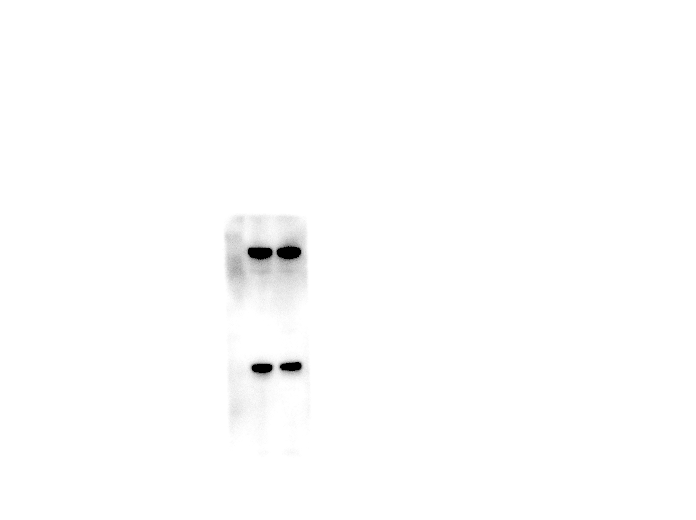

Supplement: Supplementary file 1 [file cells-10-02818-s001.zip › cells-1374880/The full bolt images for the Western Blot/caspase3/Caspse3(down) and HSP90(up)-2.tif]

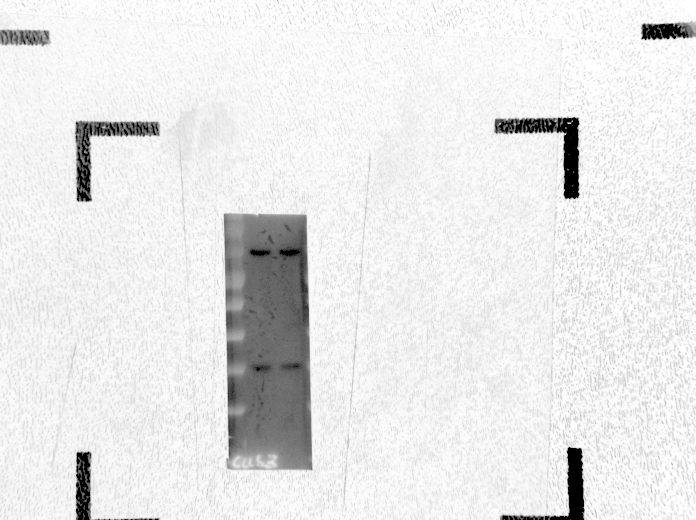

Supplement: Supplementary file 1 [file cells-10-02818-s001.zip › cells-1374880/The full bolt images for the Western Blot/caspase3/Caspse3(down) and HSP90(up)-3.tif]

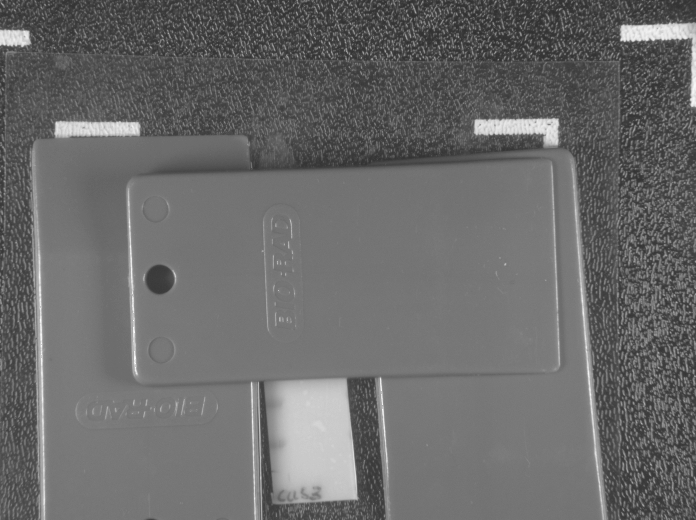

Supplement: Supplementary file 1 [file cells-10-02818-s001.zip › cells-1374880/The full bolt images for the Western Blot/caspase3/Caspse3-1.tif]

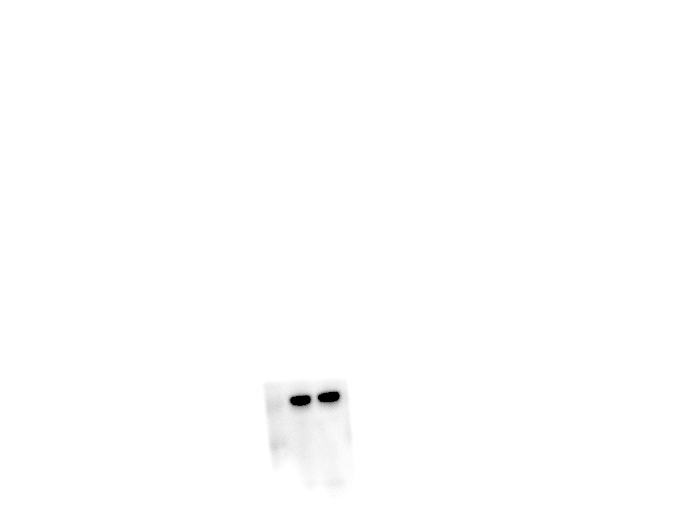

Supplement: Supplementary file 1 [file cells-10-02818-s001.zip › cells-1374880/The full bolt images for the Western Blot/caspase3/Caspse3-2.tif]

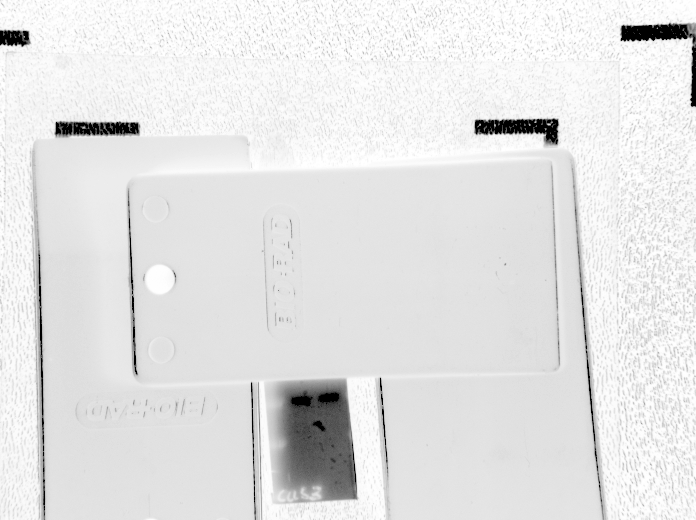

Supplement: Supplementary file 1 [file cells-10-02818-s001.zip › cells-1374880/The full bolt images for the Western Blot/caspase3/Caspse3-3.tif]

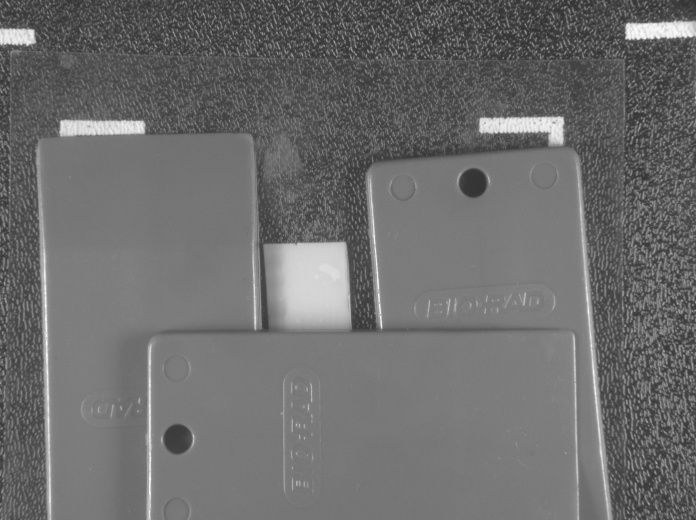

Supplement: Supplementary file 1 [file cells-10-02818-s001.zip › cells-1374880/The full bolt images for the Western Blot/caspase3/HSP90-1.tif]

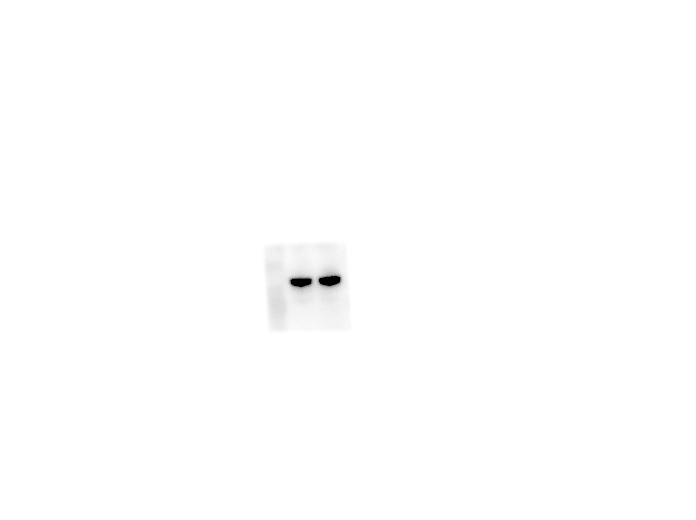

Supplement: Supplementary file 1 [file cells-10-02818-s001.zip › cells-1374880/The full bolt images for the Western Blot/caspase3/HSP90-2.tif]

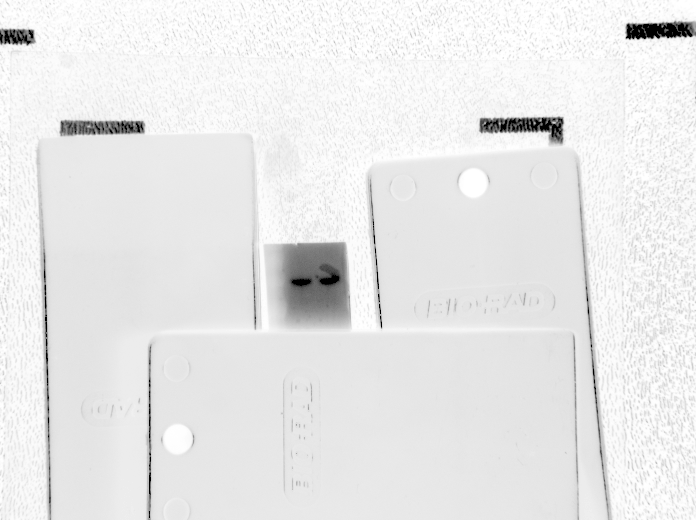

Supplement: Supplementary file 1 [file cells-10-02818-s001.zip › cells-1374880/The full bolt images for the Western Blot/caspase3/HSP90-3.tif]

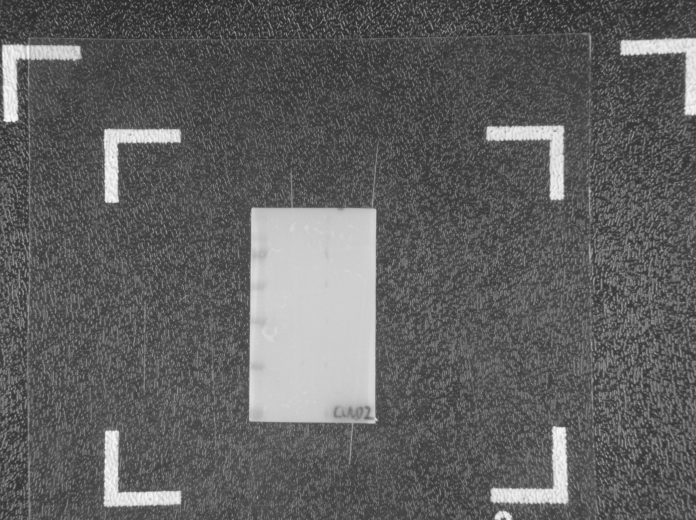

Supplement: Supplementary file 1 [file cells-10-02818-s001.zip › cells-1374880/The full bolt images for the Western Blot/CCND2/CCND2(down) and HSP90 (up)-1.tif]

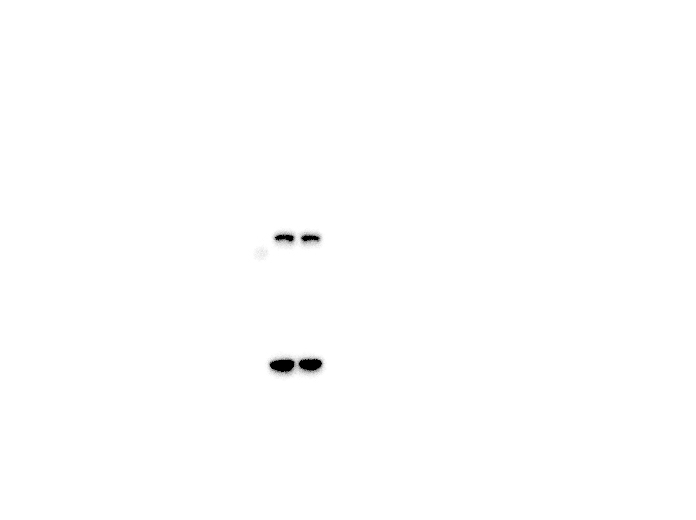

Supplement: Supplementary file 1 [file cells-10-02818-s001.zip › cells-1374880/The full bolt images for the Western Blot/CCND2/CCND2(down) and HSP90 (up)-2.tif]

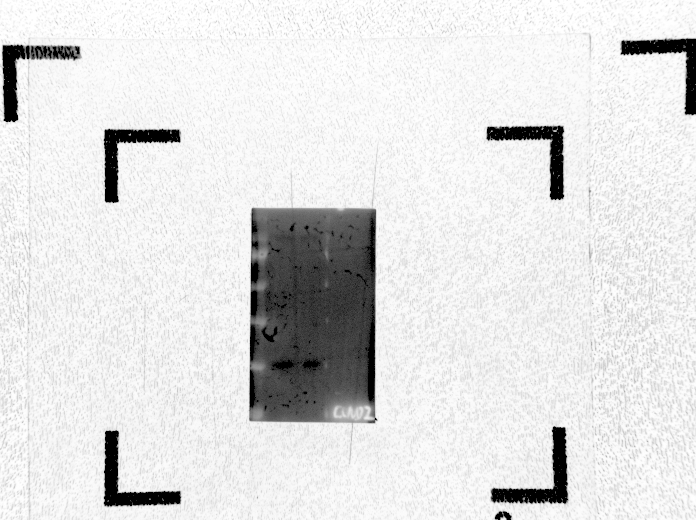

Supplement: Supplementary file 1 [file cells-10-02818-s001.zip › cells-1374880/The full bolt images for the Western Blot/CCND2/CCND2(down) and HSP90 (up)-3.tif]

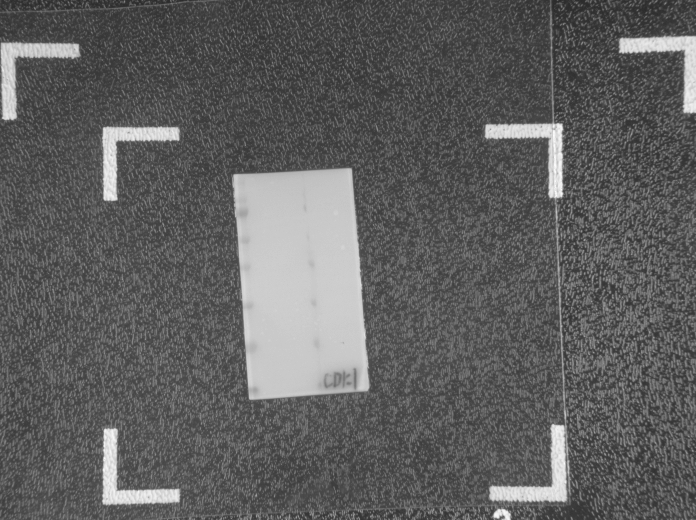

Supplement: Supplementary file 1 [file cells-10-02818-s001.zip › cells-1374880/The full bolt images for the Western Blot/CDK1/CDK1(down) and HSP90 (up)-1.tif]

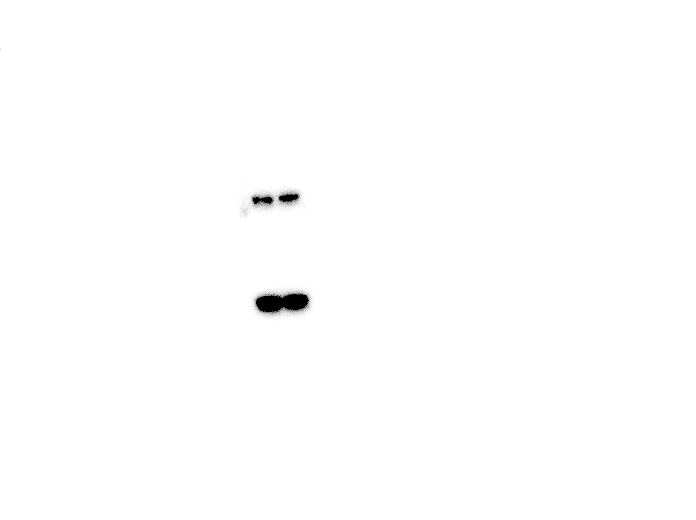

Supplement: Supplementary file 1 [file cells-10-02818-s001.zip › cells-1374880/The full bolt images for the Western Blot/CDK1/CDK1(down) and HSP90 (up)-2.tif]

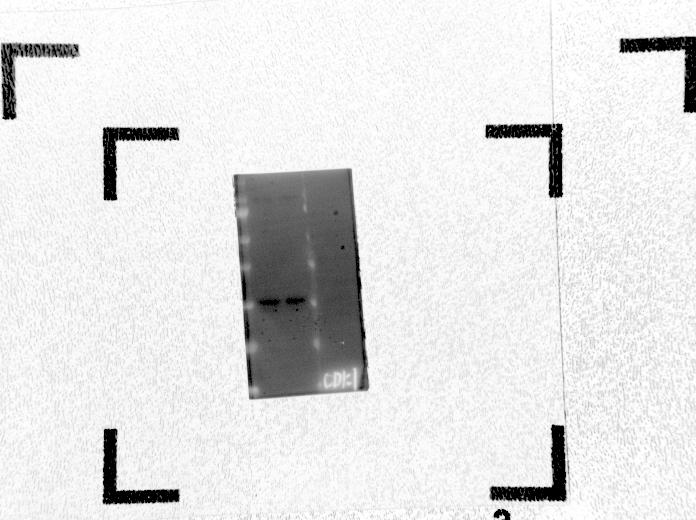

Supplement: Supplementary file 1 [file cells-10-02818-s001.zip › cells-1374880/The full bolt images for the Western Blot/CDK1/CDK1(down) and HSP90 (up)-3.tif]

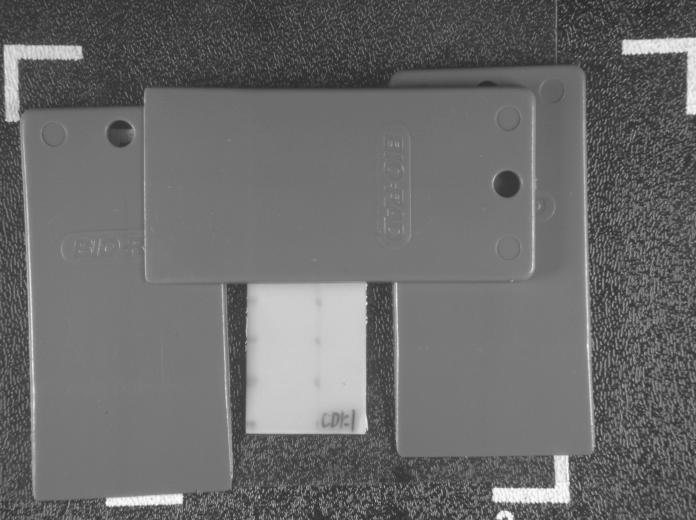

Supplement: Supplementary file 1 [file cells-10-02818-s001.zip › cells-1374880/The full bolt images for the Western Blot/CDK1/CDK1-1.tif]

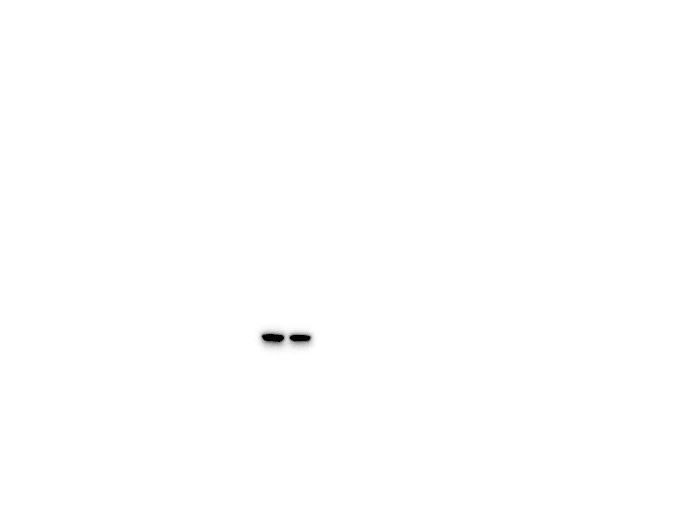

Supplement: Supplementary file 1 [file cells-10-02818-s001.zip › cells-1374880/The full bolt images for the Western Blot/CDK1/CDK1-2.tif]

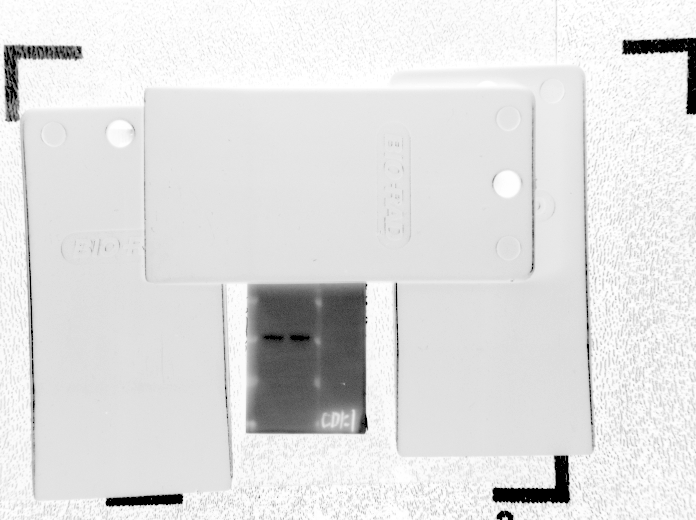

Supplement: Supplementary file 1 [file cells-10-02818-s001.zip › cells-1374880/The full bolt images for the Western Blot/CDK1/CDK1-3.tif]

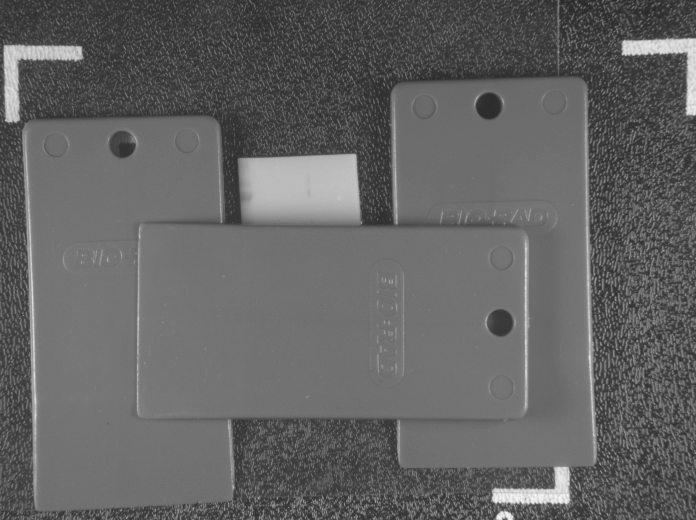

Supplement: Supplementary file 1 [file cells-10-02818-s001.zip › cells-1374880/The full bolt images for the Western Blot/CDK1/HSP90-1.tif]

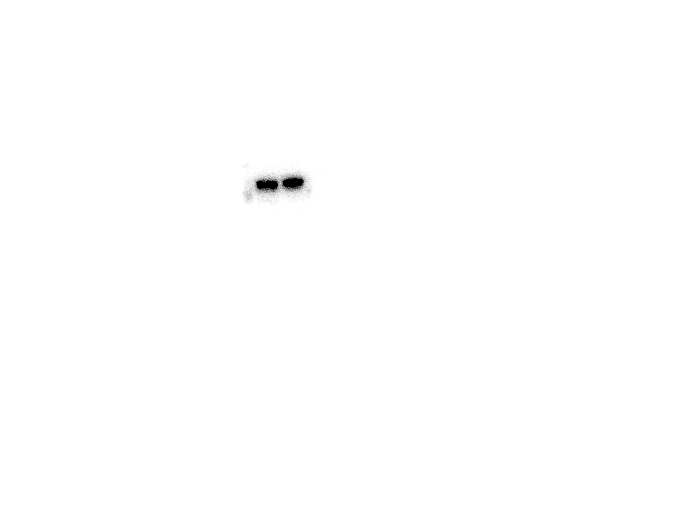

Supplement: Supplementary file 1 [file cells-10-02818-s001.zip › cells-1374880/The full bolt images for the Western Blot/CDK1/HSP90-2.tif]

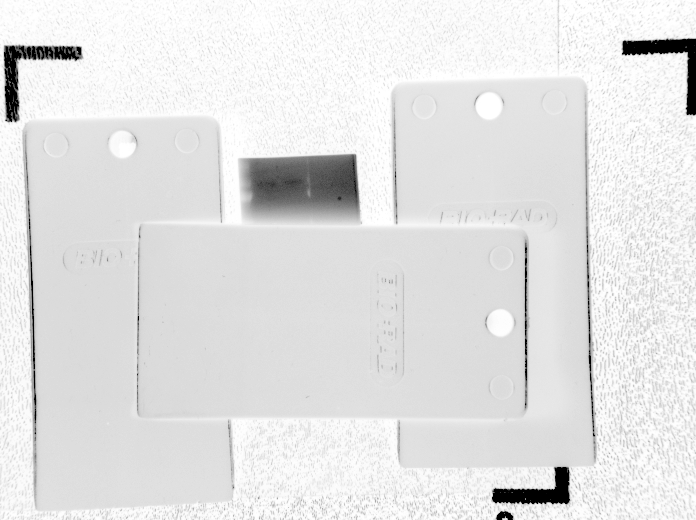

Supplement: Supplementary file 1 [file cells-10-02818-s001.zip › cells-1374880/The full bolt images for the Western Blot/CDK1/HSP90-3.tif]

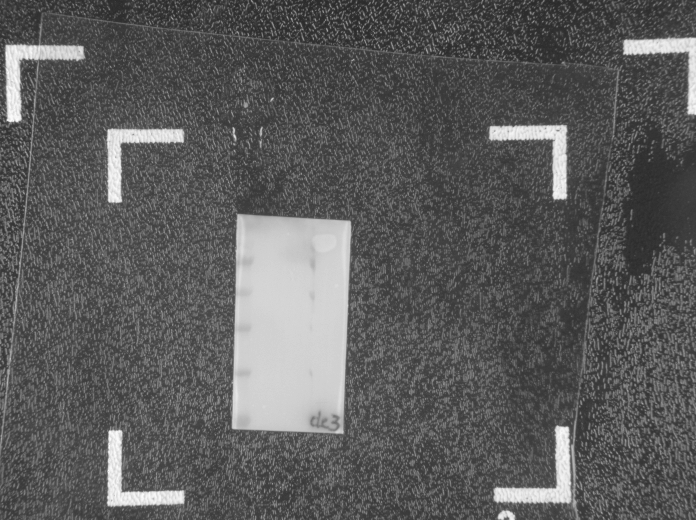

Supplement: Supplementary file 1 [file cells-10-02818-s001.zip › cells-1374880/The full bolt images for the Western Blot/Cleaved caspse3/Cleaved caspse3(down) and HSP90-1.tif]

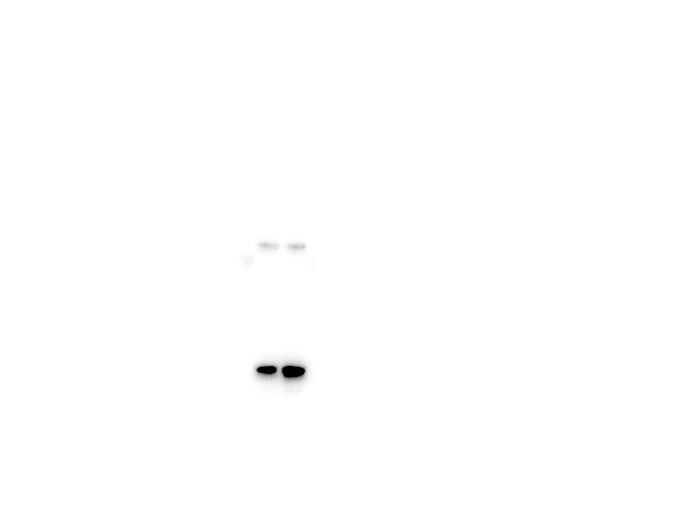

Supplement: Supplementary file 1 [file cells-10-02818-s001.zip › cells-1374880/The full bolt images for the Western Blot/Cleaved caspse3/Cleaved caspse3(down) and HSP90-2.tif]

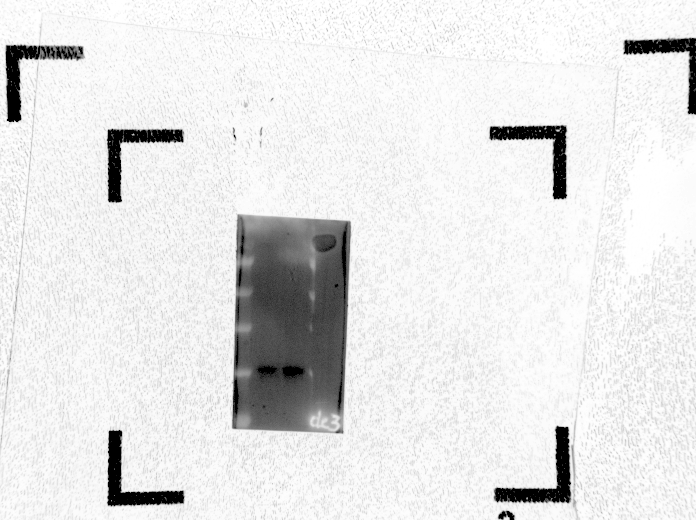

Supplement: Supplementary file 1 [file cells-10-02818-s001.zip › cells-1374880/The full bolt images for the Western Blot/Cleaved caspse3/Cleaved caspse3(down) and HSP90-3.tif]

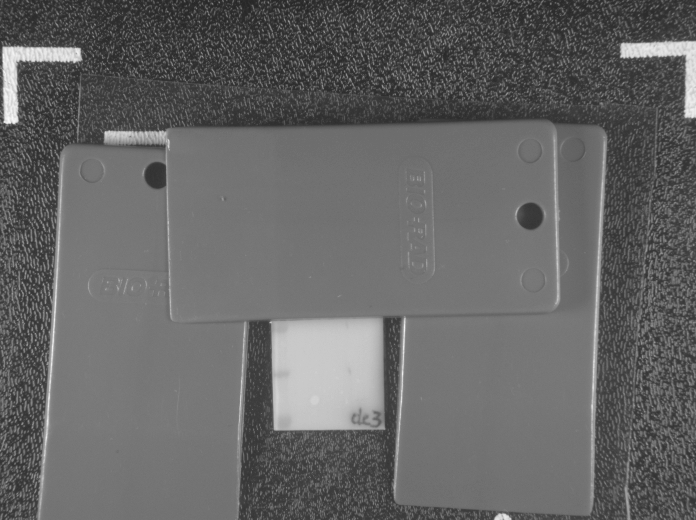

Supplement: Supplementary file 1 [file cells-10-02818-s001.zip › cells-1374880/The full bolt images for the Western Blot/Cleaved caspse3/Cleaved caspse3-1.tif]

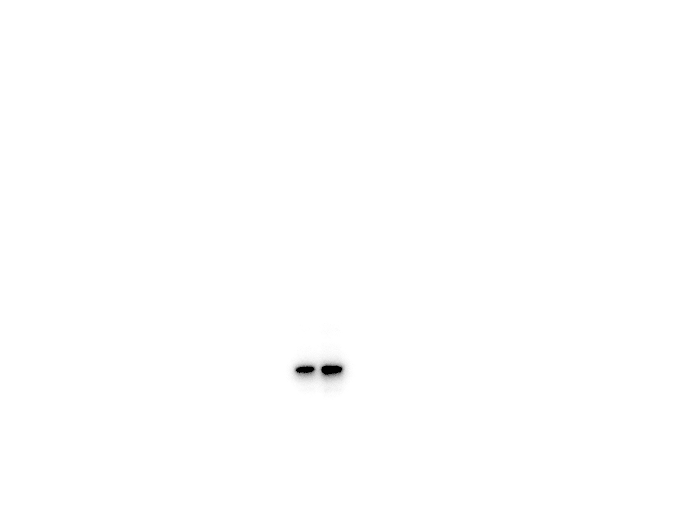

Supplement: Supplementary file 1 [file cells-10-02818-s001.zip › cells-1374880/The full bolt images for the Western Blot/Cleaved caspse3/Cleaved caspse3-2.tif]

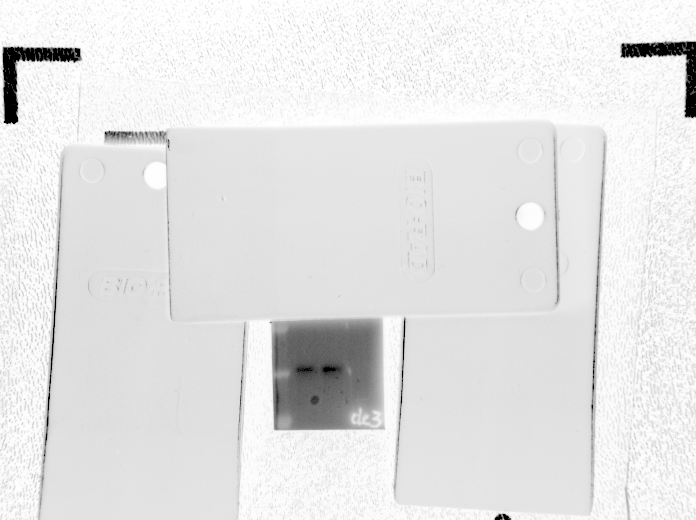

Supplement: Supplementary file 1 [file cells-10-02818-s001.zip › cells-1374880/The full bolt images for the Western Blot/Cleaved caspse3/Cleaved caspse3-3.tif]

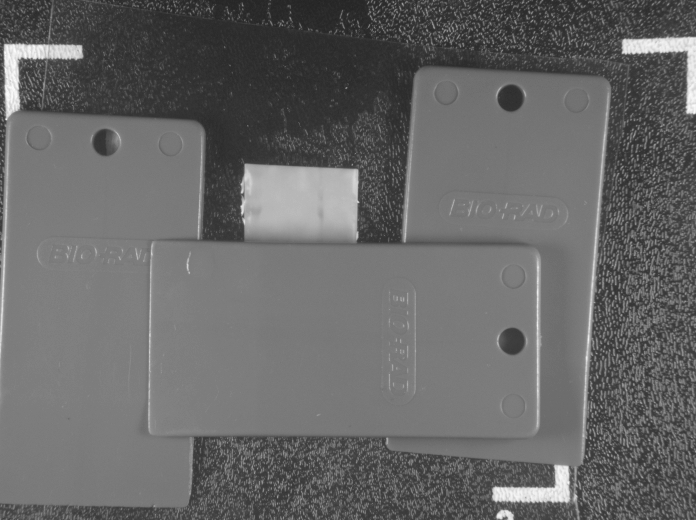

Supplement: Supplementary file 1 [file cells-10-02818-s001.zip › cells-1374880/The full bolt images for the Western Blot/Cleaved caspse3/HSP90-1.tif]

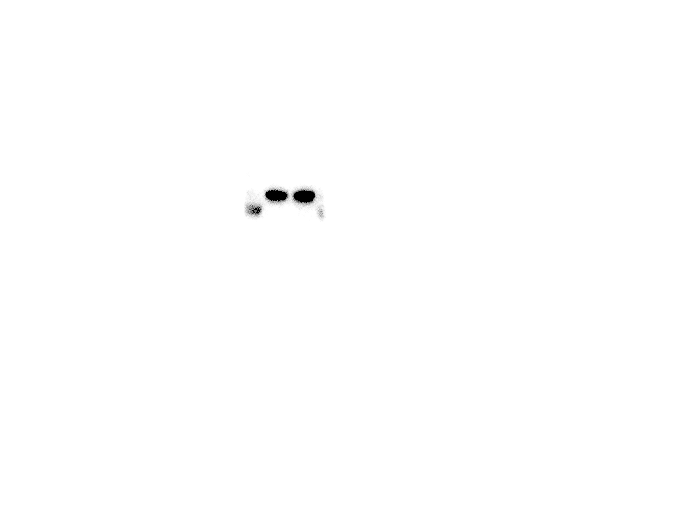

Supplement: Supplementary file 1 [file cells-10-02818-s001.zip › cells-1374880/The full bolt images for the Western Blot/Cleaved caspse3/HSP90-2.tif]

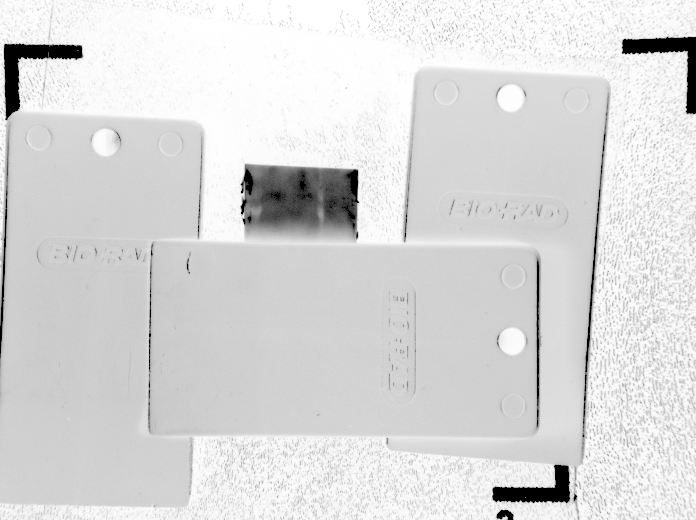

Supplement: Supplementary file 1 [file cells-10-02818-s001.zip › cells-1374880/The full bolt images for the Western Blot/Cleaved caspse3/HSP90-3.tif]

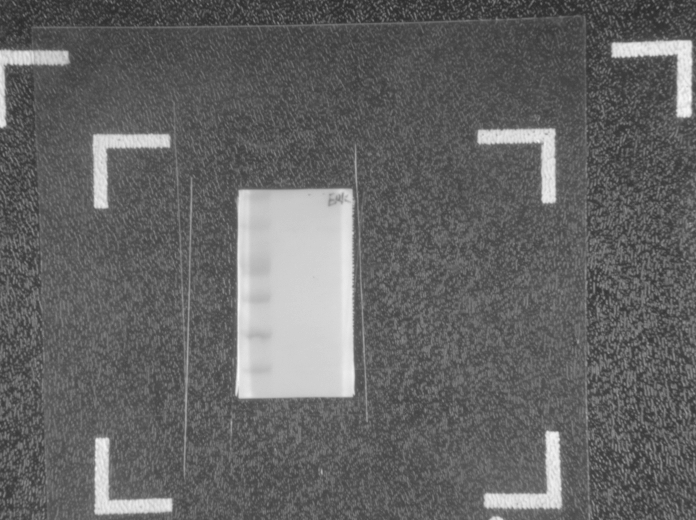

Supplement: Supplementary file 1 [file cells-10-02818-s001.zip › cells-1374880/The full bolt images for the Western Blot/ERK/ERK(down) and HSP90(up) -1.tif]

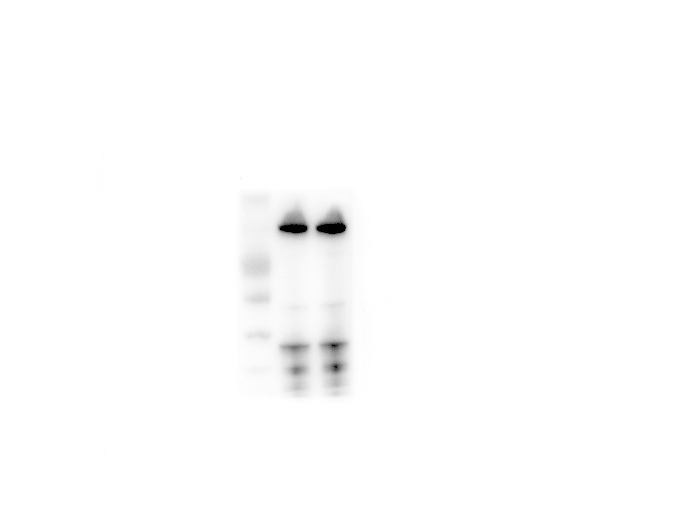

Supplement: Supplementary file 1 [file cells-10-02818-s001.zip › cells-1374880/The full bolt images for the Western Blot/ERK/ERK(down) and HSP90(up) -2.tif]

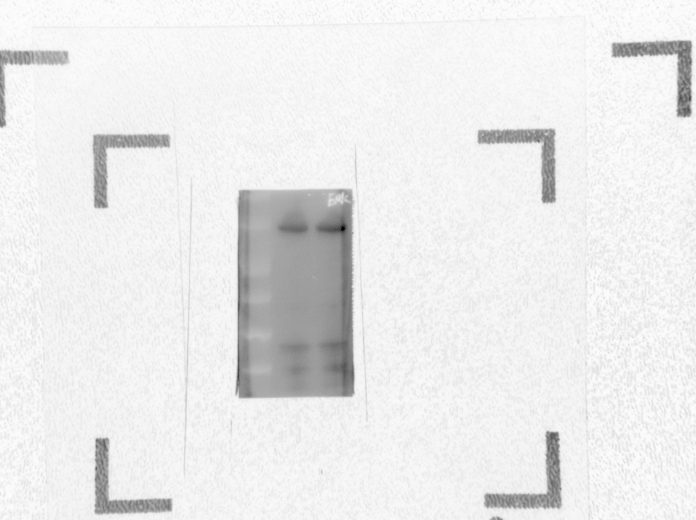

Supplement: Supplementary file 1 [file cells-10-02818-s001.zip › cells-1374880/The full bolt images for the Western Blot/ERK/ERK(down) and HSP90(up) -3.tif]

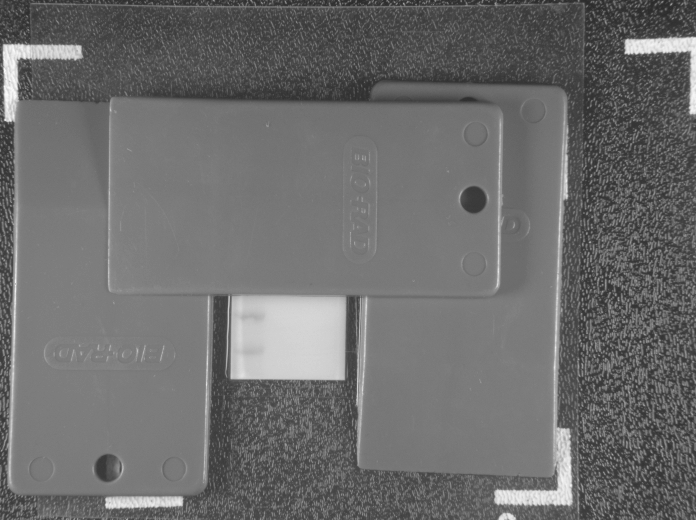

Supplement: Supplementary file 1 [file cells-10-02818-s001.zip › cells-1374880/The full bolt images for the Western Blot/ERK/ERK-1.tif]

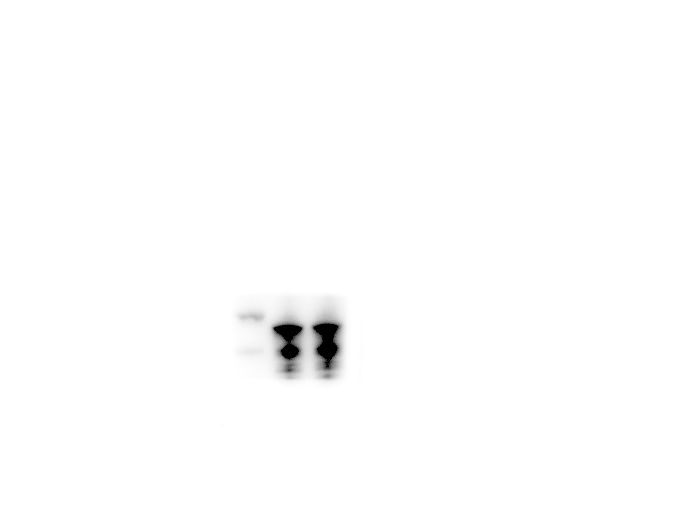

Supplement: Supplementary file 1 [file cells-10-02818-s001.zip › cells-1374880/The full bolt images for the Western Blot/ERK/ERK-2.tif]

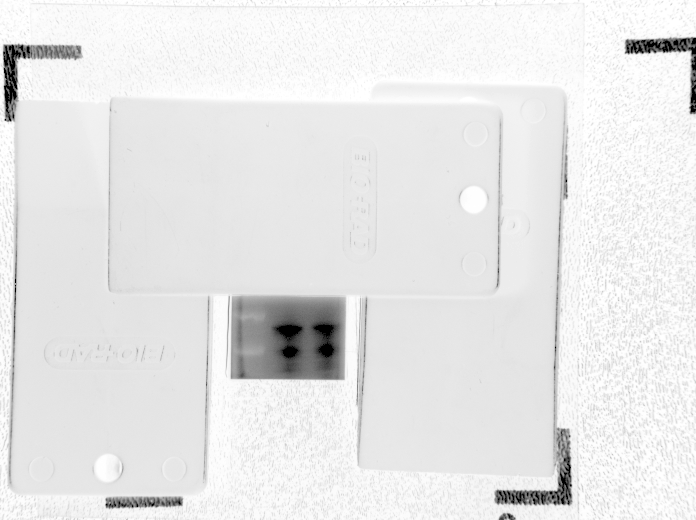

Supplement: Supplementary file 1 [file cells-10-02818-s001.zip › cells-1374880/The full bolt images for the Western Blot/ERK/ERK-3.tif]

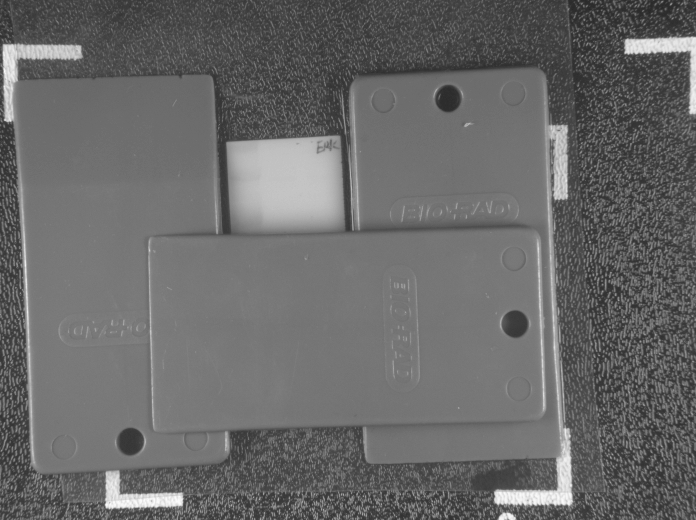

Supplement: Supplementary file 1 [file cells-10-02818-s001.zip › cells-1374880/The full bolt images for the Western Blot/ERK/HSP90-1.tif]

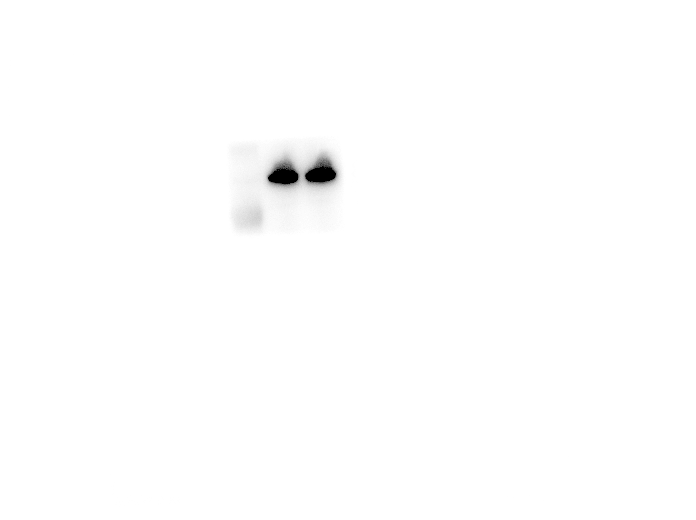

Supplement: Supplementary file 1 [file cells-10-02818-s001.zip › cells-1374880/The full bolt images for the Western Blot/ERK/HSP90-2.tif]

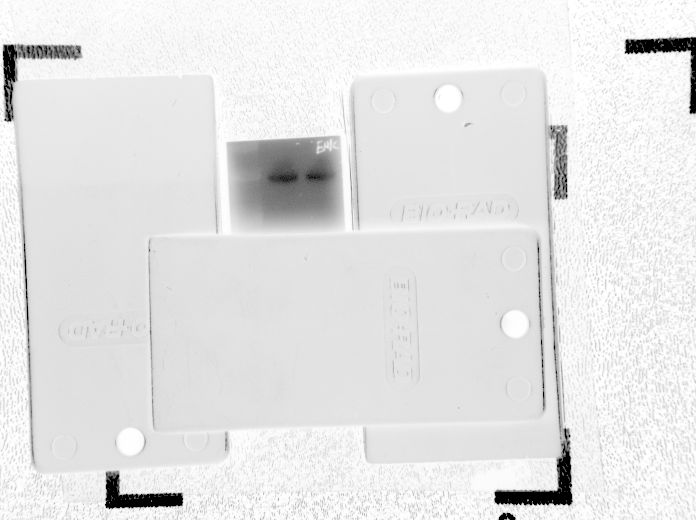

Supplement: Supplementary file 1 [file cells-10-02818-s001.zip › cells-1374880/The full bolt images for the Western Blot/ERK/HSP90-3.tif]

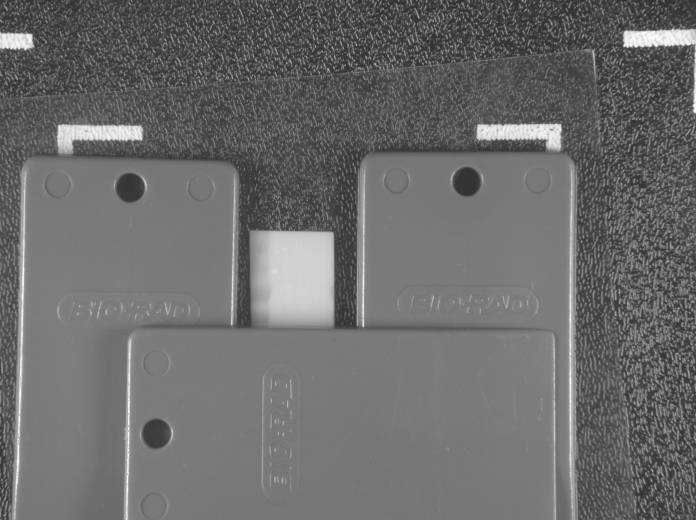

Supplement: Supplementary file 1 [file cells-10-02818-s001.zip › cells-1374880/The full bolt images for the Western Blot/IKB/HSP90-1.tif]

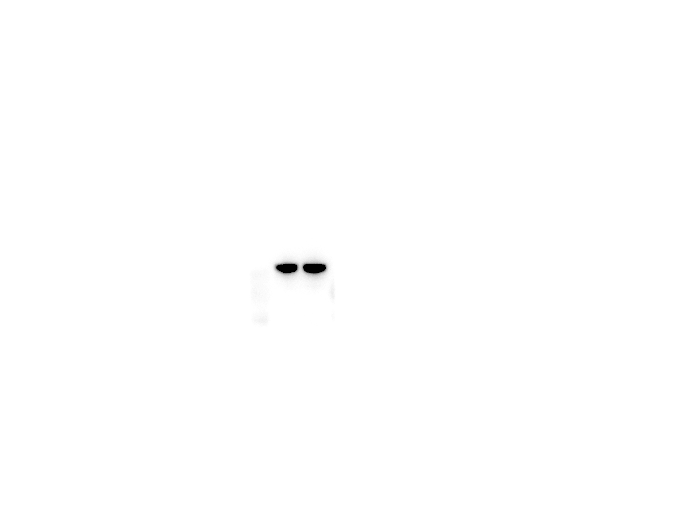

Supplement: Supplementary file 1 [file cells-10-02818-s001.zip › cells-1374880/The full bolt images for the Western Blot/IKB/HSP90-2.tif]

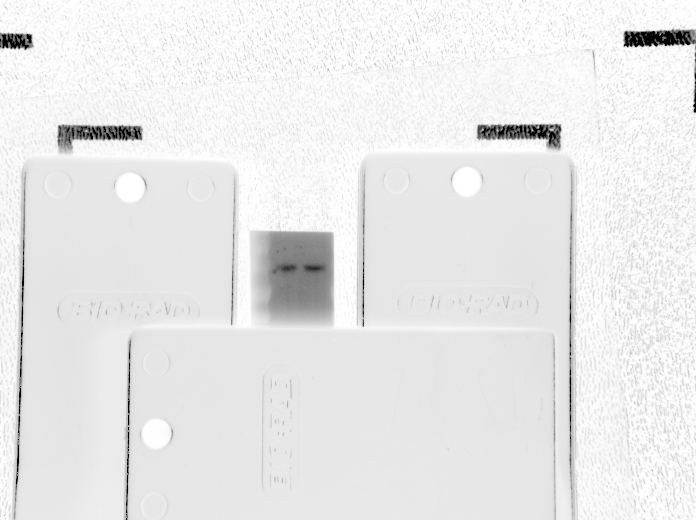

Supplement: Supplementary file 1 [file cells-10-02818-s001.zip › cells-1374880/The full bolt images for the Western Blot/IKB/HSP90-3.tif]

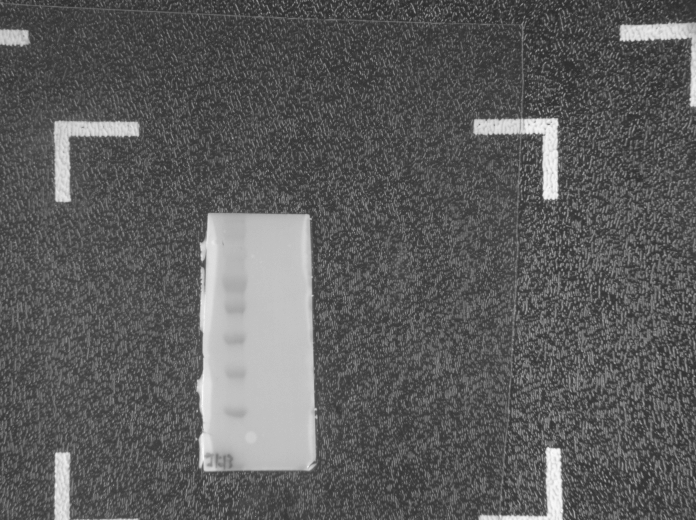

Supplement: Supplementary file 1 [file cells-10-02818-s001.zip › cells-1374880/The full bolt images for the Western Blot/IKB/IKB(down) and HSP90 (up)-1.tif]

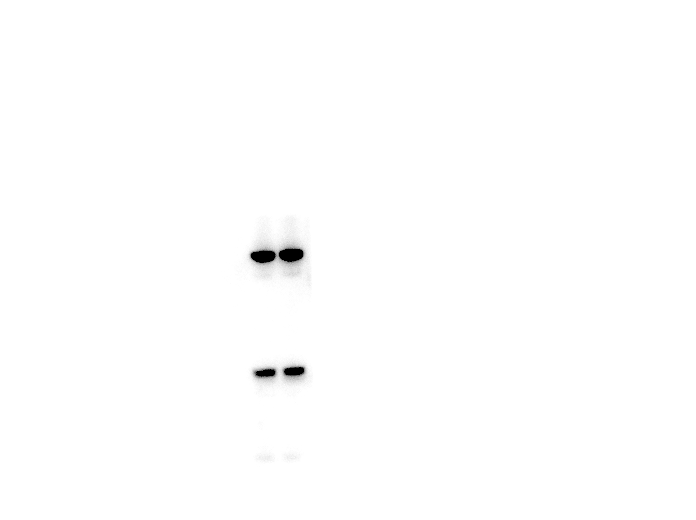

Supplement: Supplementary file 1 [file cells-10-02818-s001.zip › cells-1374880/The full bolt images for the Western Blot/IKB/IKB(down) and HSP90 (up)-2.tif]

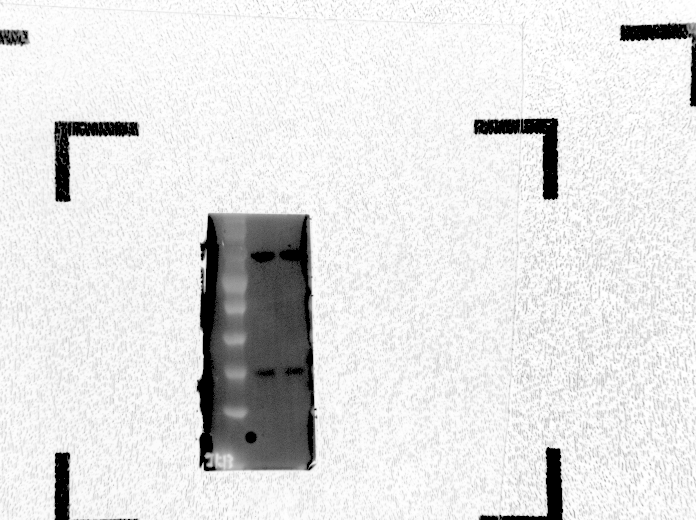

Supplement: Supplementary file 1 [file cells-10-02818-s001.zip › cells-1374880/The full bolt images for the Western Blot/IKB/IKB(down) and HSP90 (up)-3.tif]

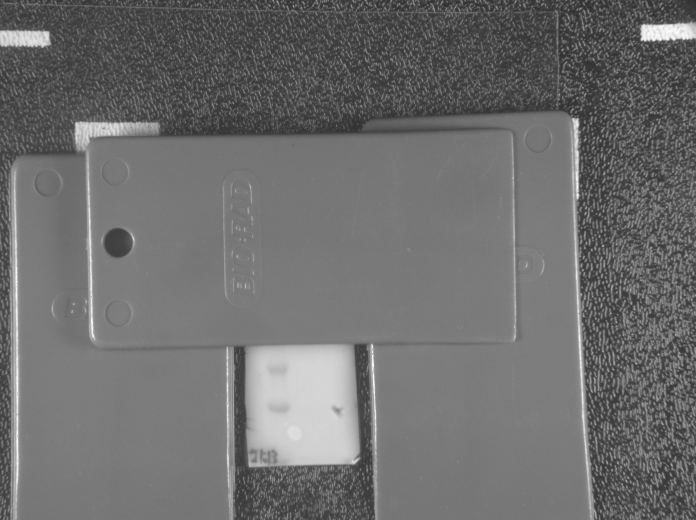

Supplement: Supplementary file 1 [file cells-10-02818-s001.zip › cells-1374880/The full bolt images for the Western Blot/IKB/IKB-1.tif]

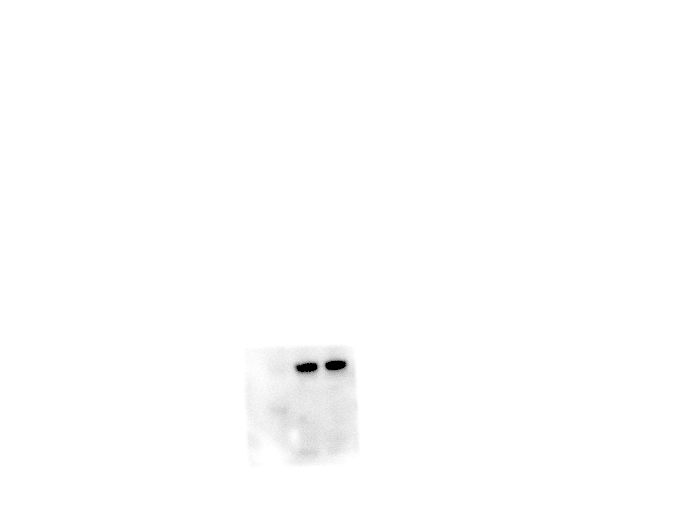

Supplement: Supplementary file 1 [file cells-10-02818-s001.zip › cells-1374880/The full bolt images for the Western Blot/IKB/IKB-2.tif]

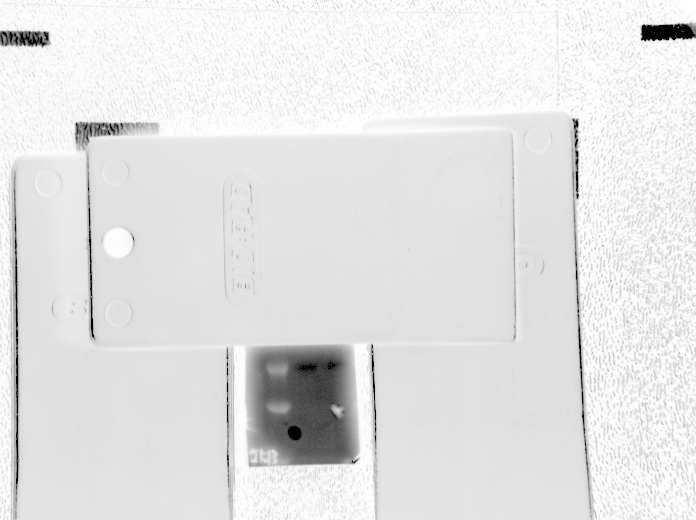

Supplement: Supplementary file 1 [file cells-10-02818-s001.zip › cells-1374880/The full bolt images for the Western Blot/IKB/IKB-3.tif]

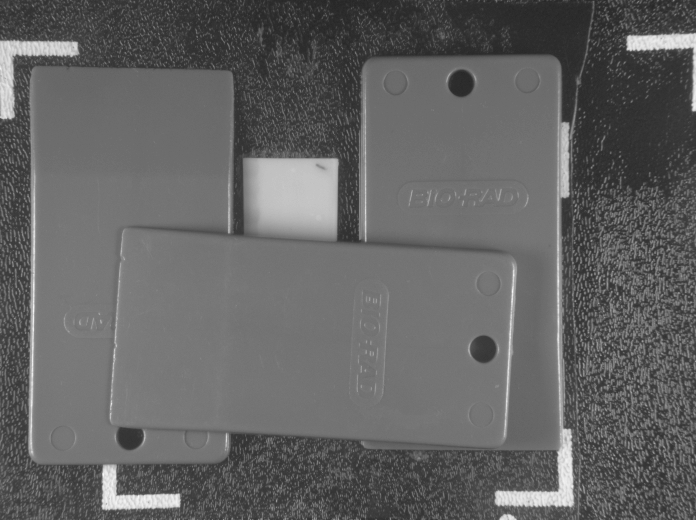

Supplement: Supplementary file 1 [file cells-10-02818-s001.zip › cells-1374880/The full bolt images for the Western Blot/JNK/HSP90-1.tif]

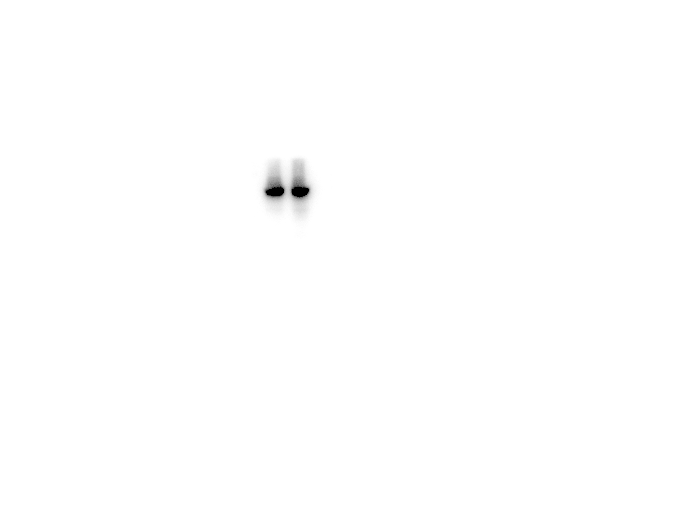

Supplement: Supplementary file 1 [file cells-10-02818-s001.zip › cells-1374880/The full bolt images for the Western Blot/JNK/HSP90-2.tif]

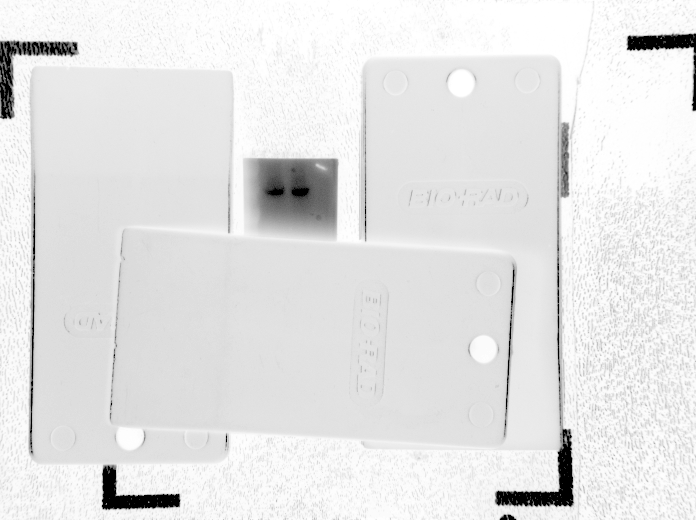

Supplement: Supplementary file 1 [file cells-10-02818-s001.zip › cells-1374880/The full bolt images for the Western Blot/JNK/HSP90-3.tif]

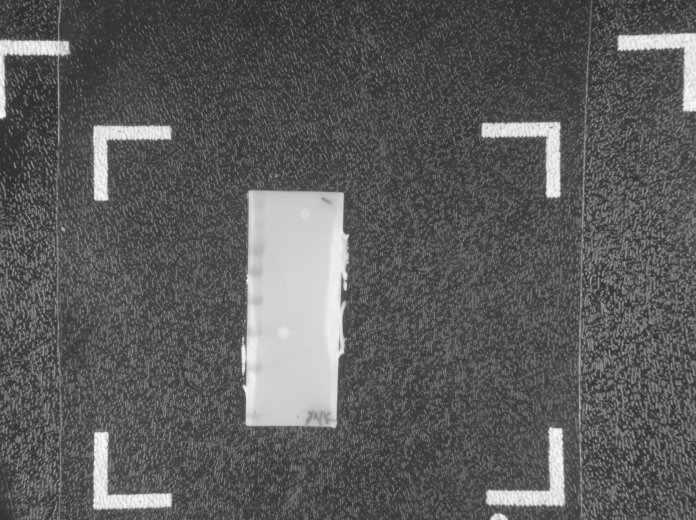

Supplement: Supplementary file 1 [file cells-10-02818-s001.zip › cells-1374880/The full bolt images for the Western Blot/JNK/JNK(down) and HSP90(up)-1.tif]

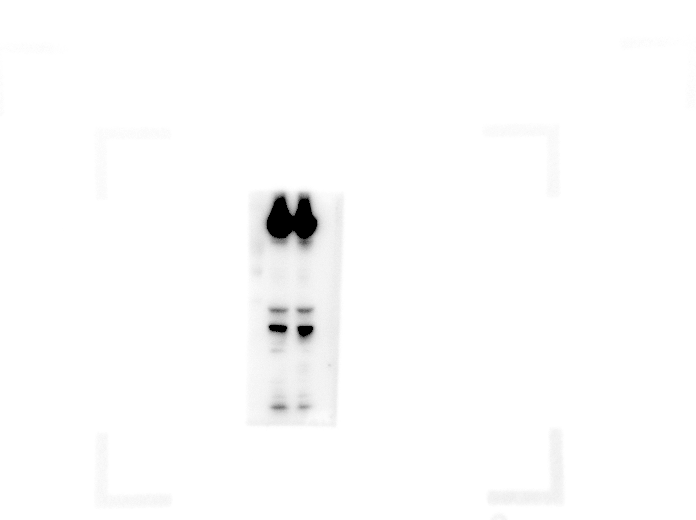

Supplement: Supplementary file 1 [file cells-10-02818-s001.zip › cells-1374880/The full bolt images for the Western Blot/JNK/JNK(down) and HSP90(up)-2.tif]

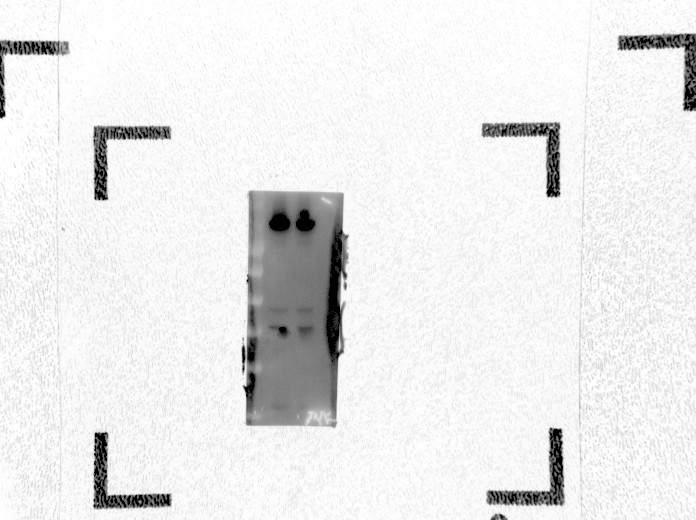

Supplement: Supplementary file 1 [file cells-10-02818-s001.zip › cells-1374880/The full bolt images for the Western Blot/JNK/JNK(down) and HSP90(up)-3.tif]

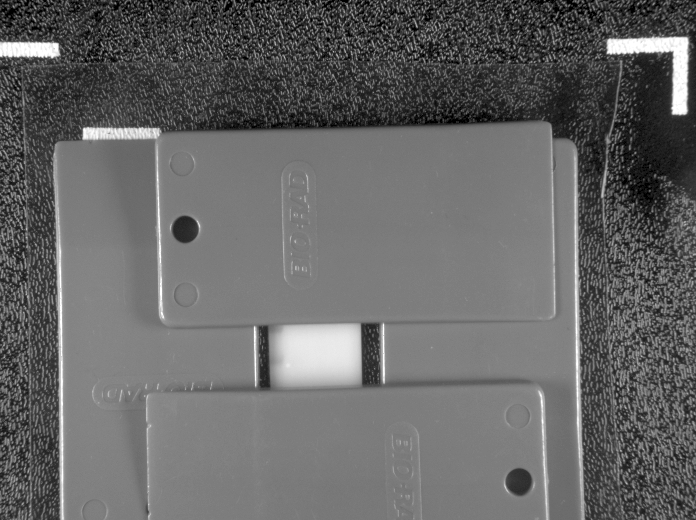

Supplement: Supplementary file 1 [file cells-10-02818-s001.zip › cells-1374880/The full bolt images for the Western Blot/JNK/JNK-1.tif]

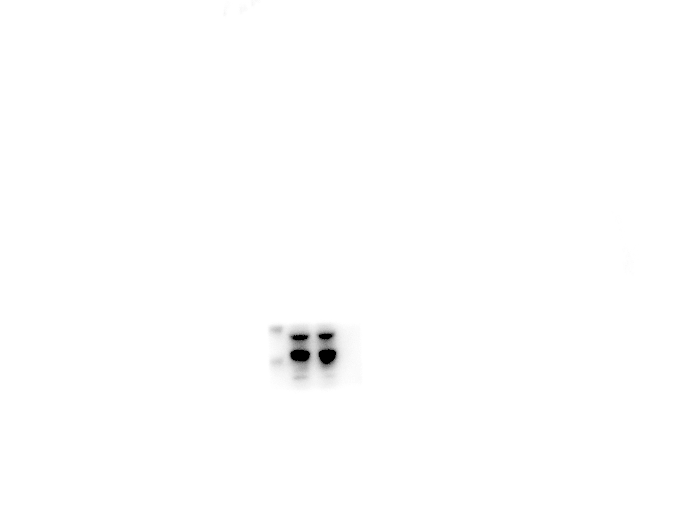

Supplement: Supplementary file 1 [file cells-10-02818-s001.zip › cells-1374880/The full bolt images for the Western Blot/JNK/JNK-2.tif]

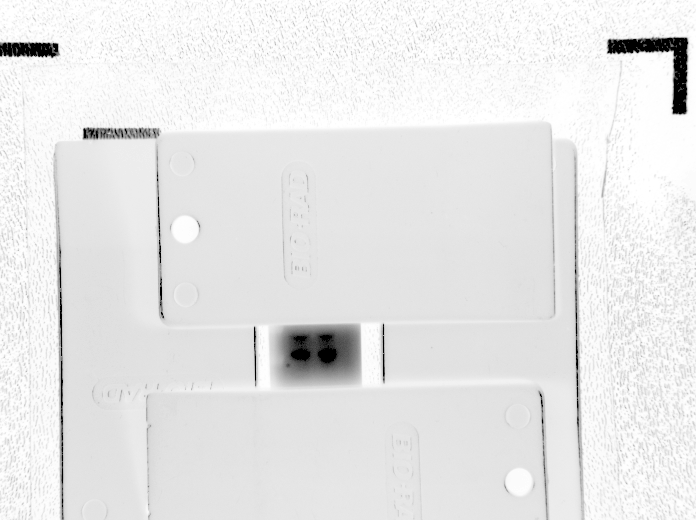

Supplement: Supplementary file 1 [file cells-10-02818-s001.zip › cells-1374880/The full bolt images for the Western Blot/JNK/JNK-3.tif]

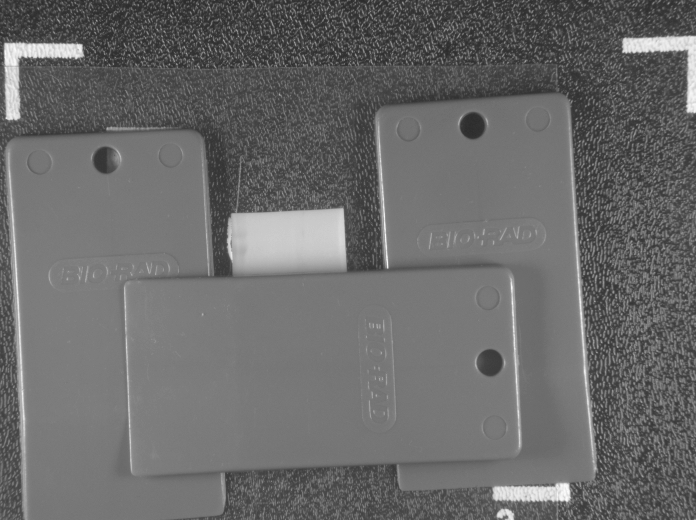

Supplement: Supplementary file 1 [file cells-10-02818-s001.zip › cells-1374880/The full bolt images for the Western Blot/p-ERK/HSP90-1.tif]

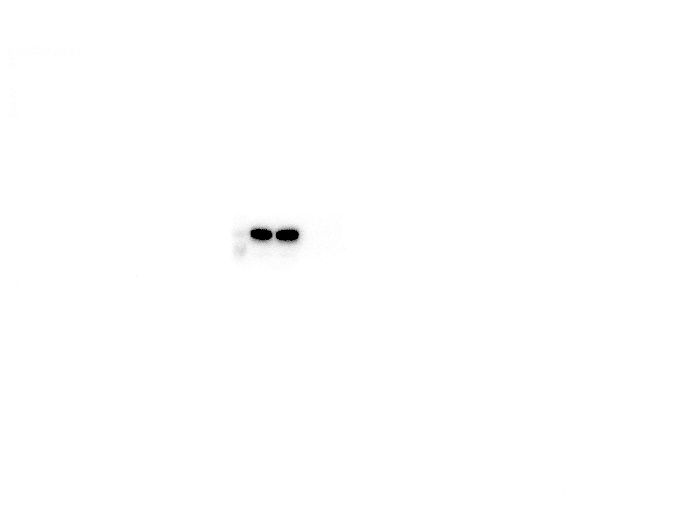

Supplement: Supplementary file 1 [file cells-10-02818-s001.zip › cells-1374880/The full bolt images for the Western Blot/p-ERK/HSP90-2.tif]

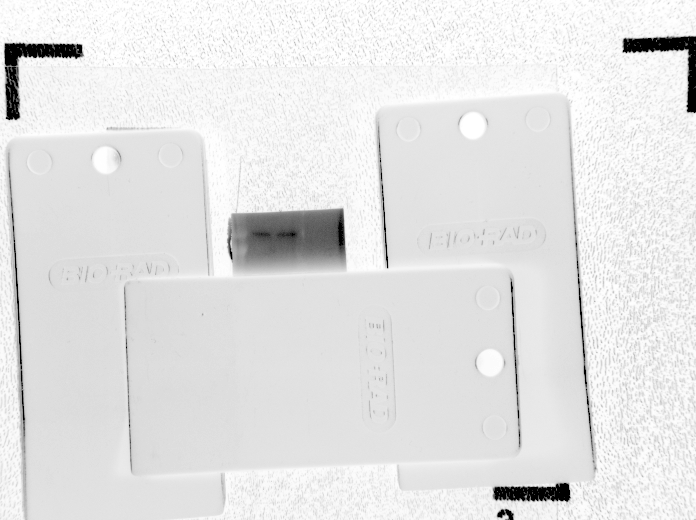

Supplement: Supplementary file 1 [file cells-10-02818-s001.zip › cells-1374880/The full bolt images for the Western Blot/p-ERK/HSP90-3.tif]

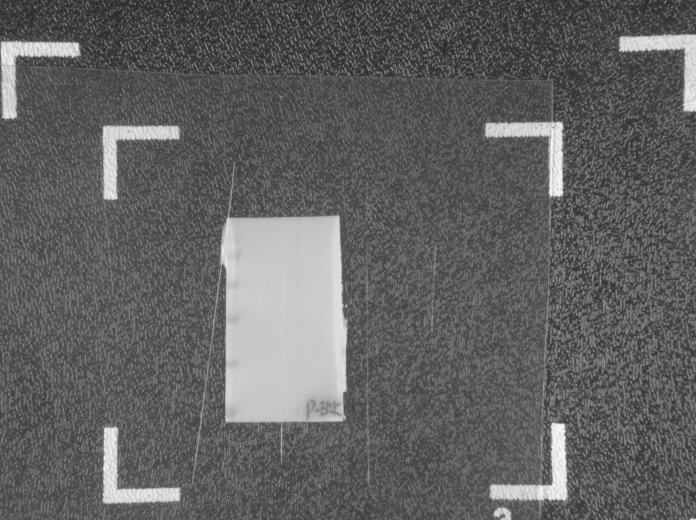

Supplement: Supplementary file 1 [file cells-10-02818-s001.zip › cells-1374880/The full bolt images for the Western Blot/p-ERK/p-ERK(down) and HSP90(up)-1.tif]

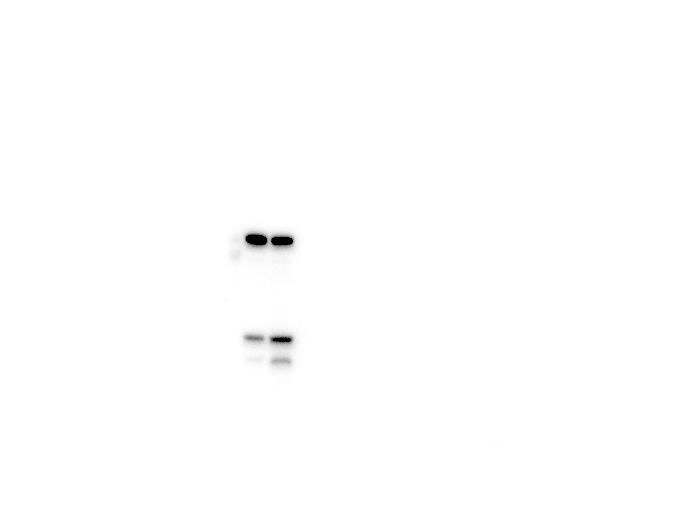

Supplement: Supplementary file 1 [file cells-10-02818-s001.zip › cells-1374880/The full bolt images for the Western Blot/p-ERK/p-ERK(down) and HSP90(up)-2.tif]

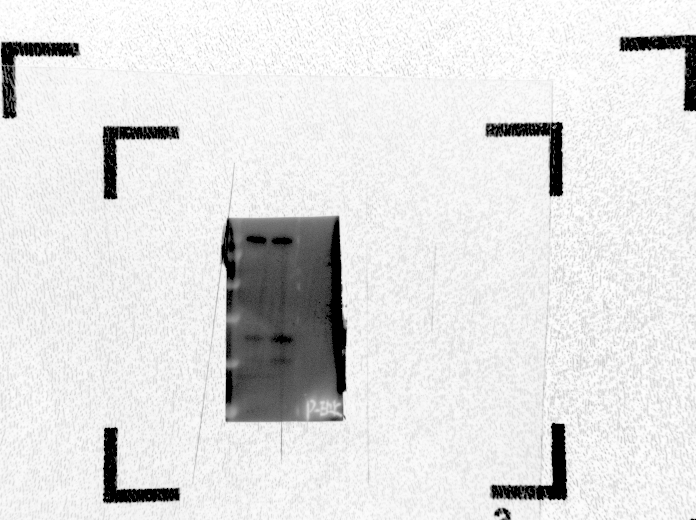

Supplement: Supplementary file 1 [file cells-10-02818-s001.zip › cells-1374880/The full bolt images for the Western Blot/p-ERK/p-ERK(down) and HSP90(up)-3.tif]

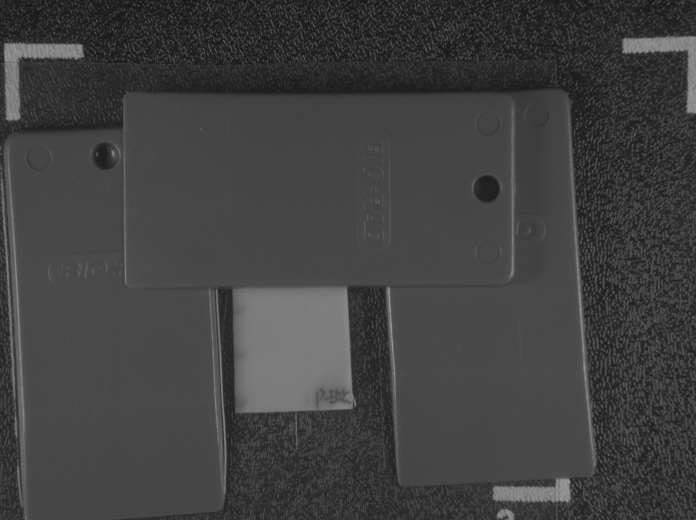

Supplement: Supplementary file 1 [file cells-10-02818-s001.zip › cells-1374880/The full bolt images for the Western Blot/p-ERK/p-ERK-1.tif]

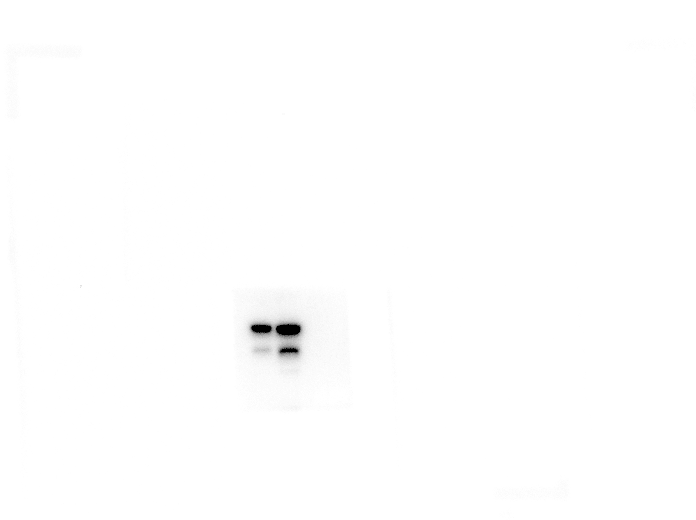

Supplement: Supplementary file 1 [file cells-10-02818-s001.zip › cells-1374880/The full bolt images for the Western Blot/p-ERK/p-ERK-2.tif]

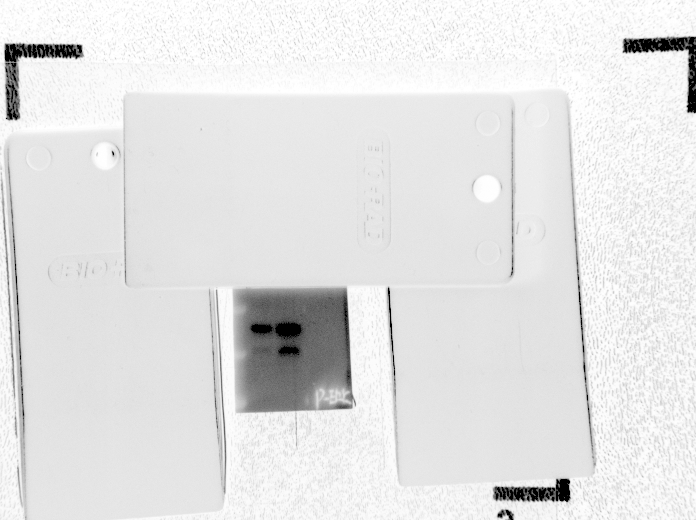

Supplement: Supplementary file 1 [file cells-10-02818-s001.zip › cells-1374880/The full bolt images for the Western Blot/p-ERK/p-ERK-3.tif]

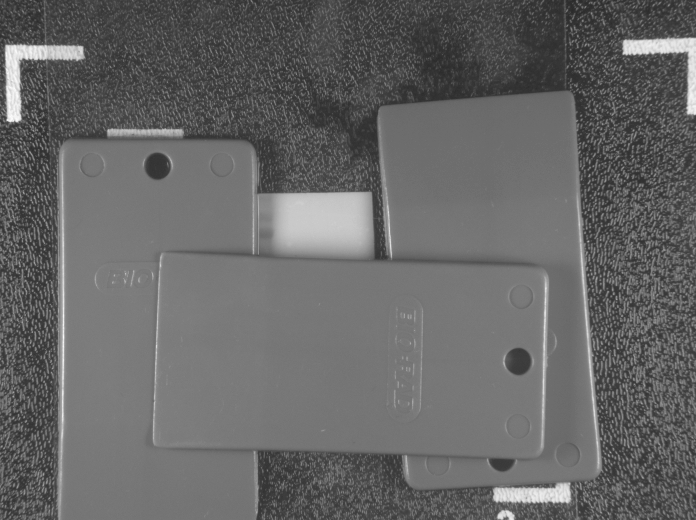

Supplement: Supplementary file 1 [file cells-10-02818-s001.zip › cells-1374880/The full bolt images for the Western Blot/p-IKB/HSP90-1.tif]

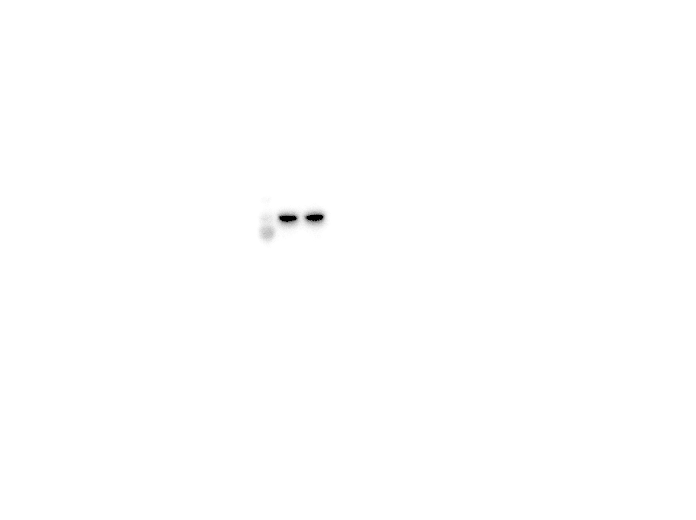

Supplement: Supplementary file 1 [file cells-10-02818-s001.zip › cells-1374880/The full bolt images for the Western Blot/p-IKB/HSP90-2.tif]

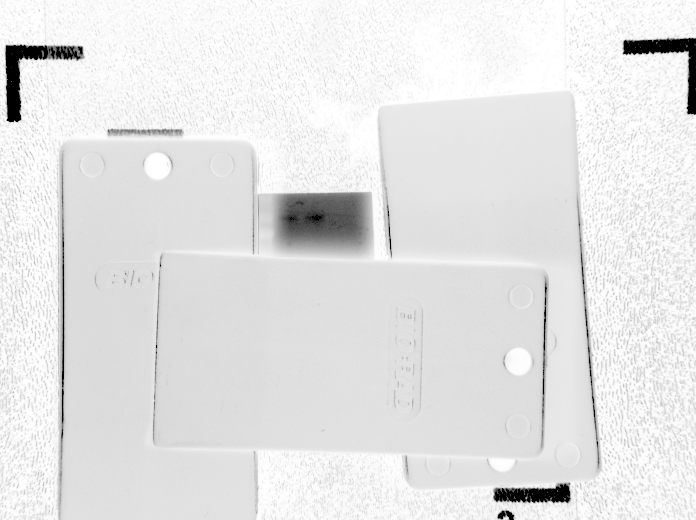

Supplement: Supplementary file 1 [file cells-10-02818-s001.zip › cells-1374880/The full bolt images for the Western Blot/p-IKB/HSP90-3.tif]

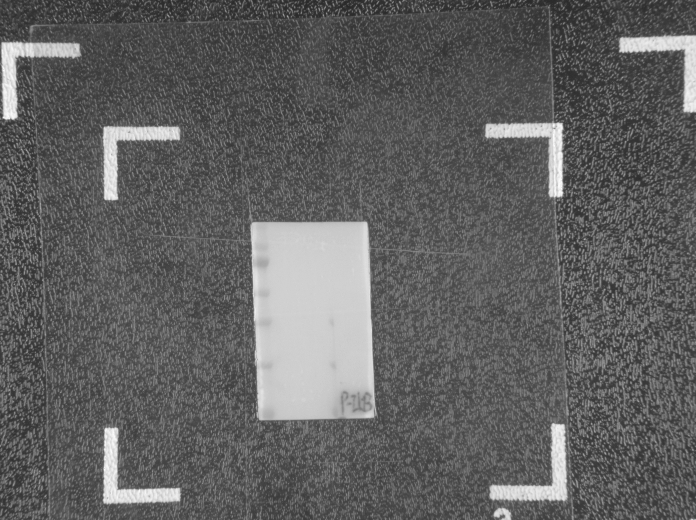

Supplement: Supplementary file 1 [file cells-10-02818-s001.zip › cells-1374880/The full bolt images for the Western Blot/p-IKB/p-IKB(down) and HSP90(up)-1.tif]

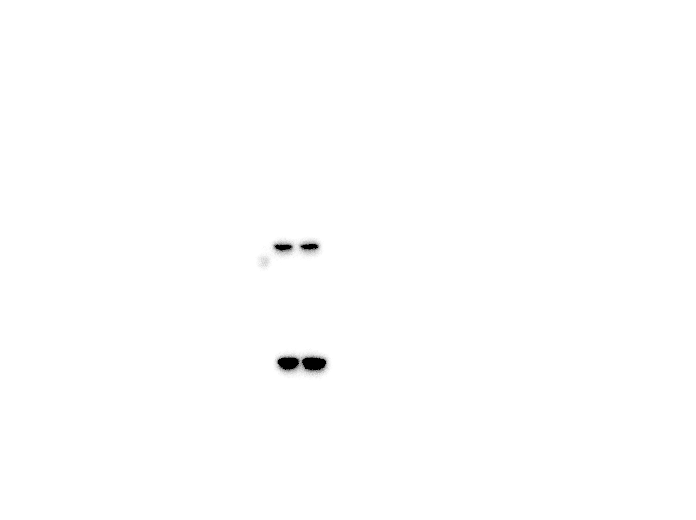

Supplement: Supplementary file 1 [file cells-10-02818-s001.zip › cells-1374880/The full bolt images for the Western Blot/p-IKB/p-IKB(down) and HSP90(up)-2.tif]

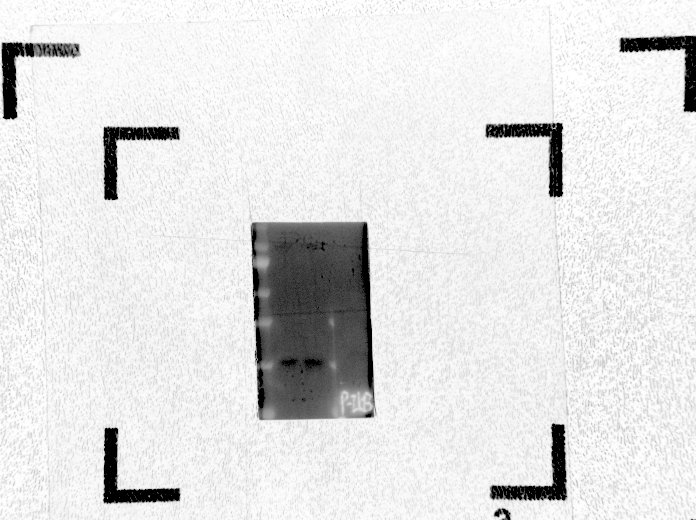

Supplement: Supplementary file 1 [file cells-10-02818-s001.zip › cells-1374880/The full bolt images for the Western Blot/p-IKB/p-IKB(down) and HSP90(up)-3.tif]

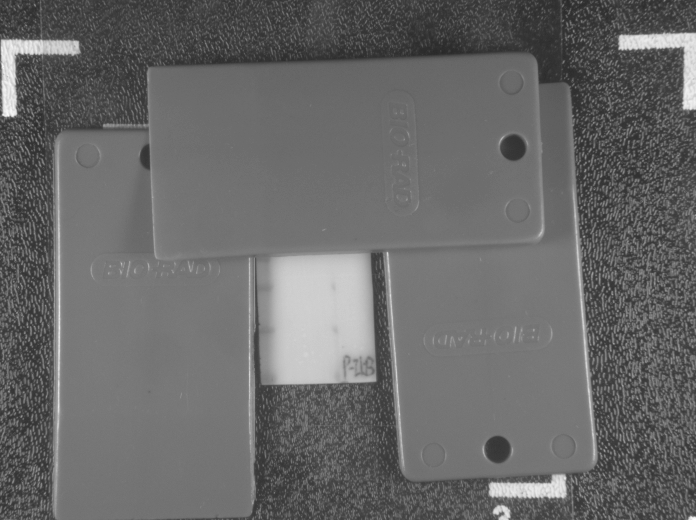

Supplement: Supplementary file 1 [file cells-10-02818-s001.zip › cells-1374880/The full bolt images for the Western Blot/p-IKB/p-IKB-1.tif]

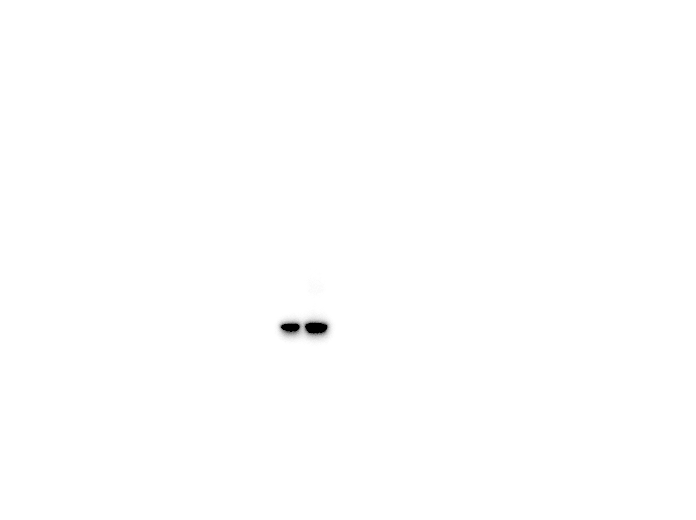

Supplement: Supplementary file 1 [file cells-10-02818-s001.zip › cells-1374880/The full bolt images for the Western Blot/p-IKB/p-IKB-2.tif]

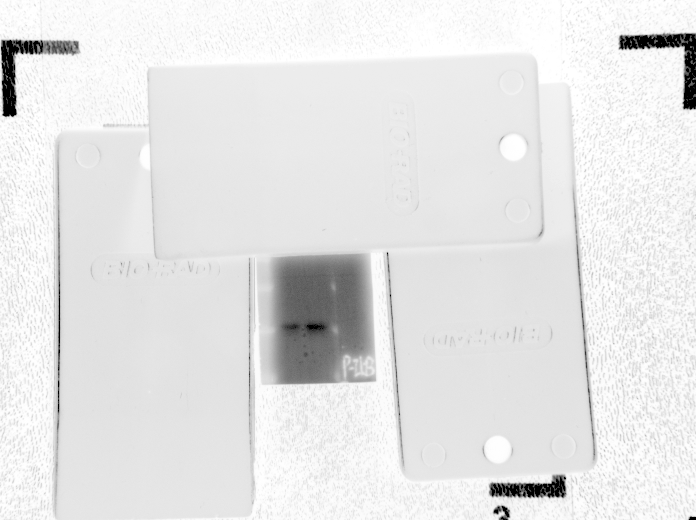

Supplement: Supplementary file 1 [file cells-10-02818-s001.zip › cells-1374880/The full bolt images for the Western Blot/p-IKB/p-IKB-3.tif]

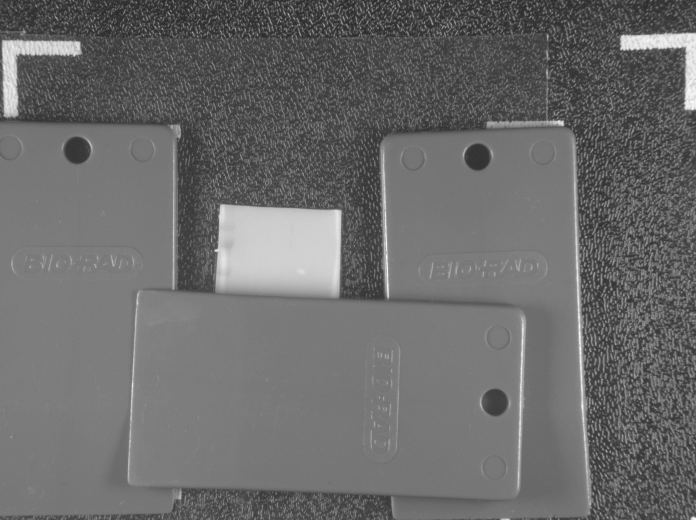

Supplement: Supplementary file 1 [file cells-10-02818-s001.zip › cells-1374880/The full bolt images for the Western Blot/P-JNK/HSP90-1.tif]

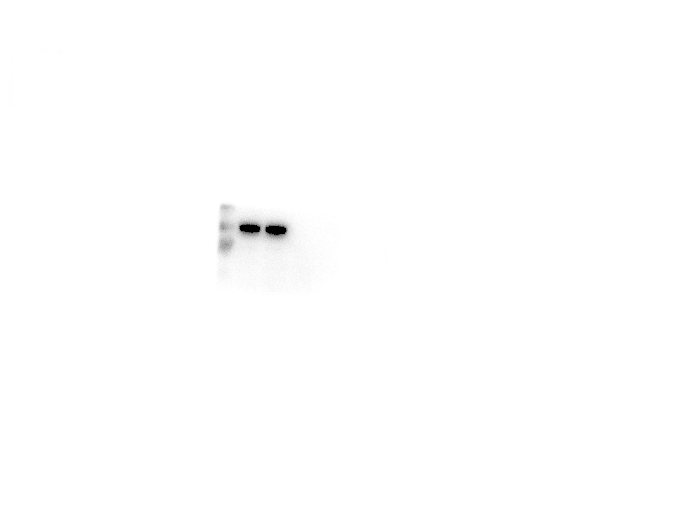

Supplement: Supplementary file 1 [file cells-10-02818-s001.zip › cells-1374880/The full bolt images for the Western Blot/P-JNK/HSP90-2.tif]

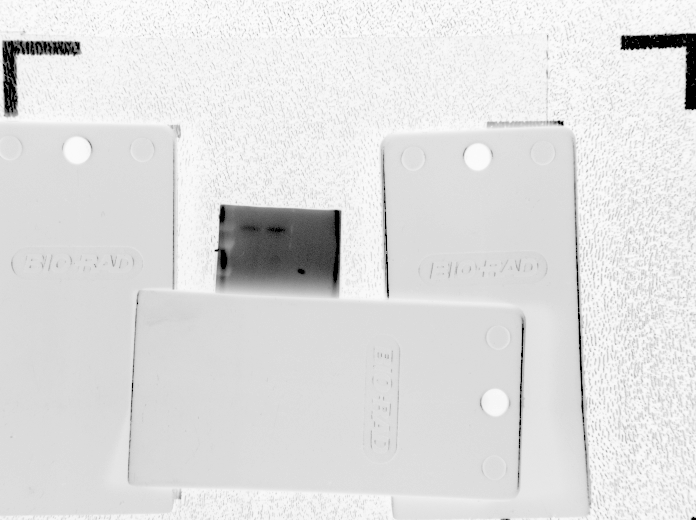

Supplement: Supplementary file 1 [file cells-10-02818-s001.zip › cells-1374880/The full bolt images for the Western Blot/P-JNK/HSP90-3.tif]

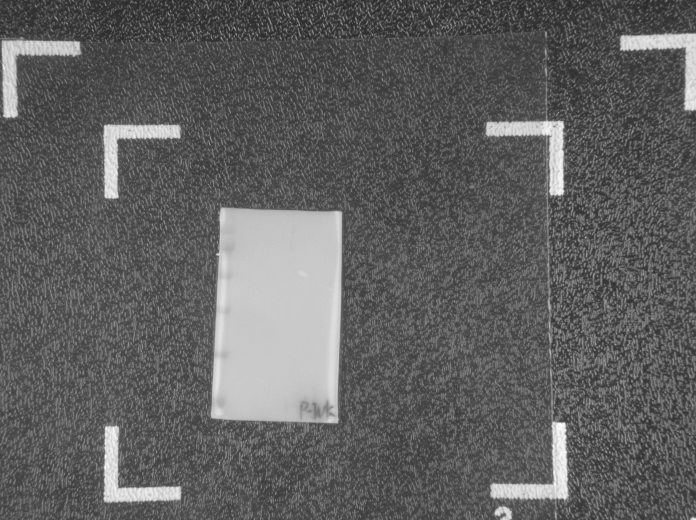

Supplement: Supplementary file 1 [file cells-10-02818-s001.zip › cells-1374880/The full bolt images for the Western Blot/P-JNK/P-JNK(down) and HSP90(up) -1.tif]

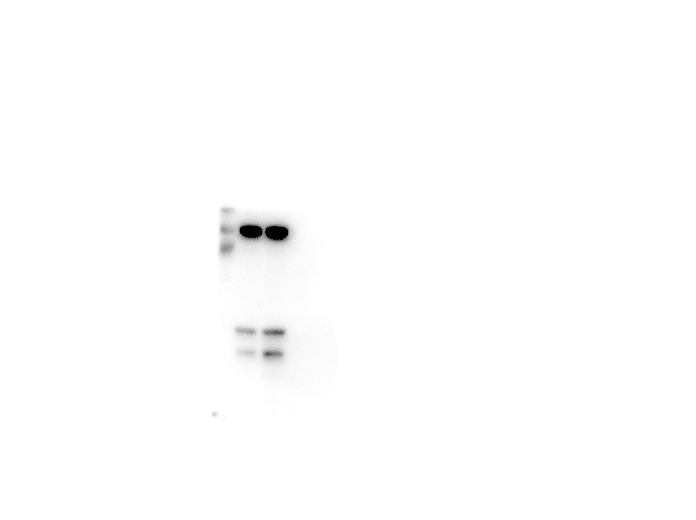

Supplement: Supplementary file 1 [file cells-10-02818-s001.zip › cells-1374880/The full bolt images for the Western Blot/P-JNK/P-JNK(down) and HSP90(up) -2.tif]

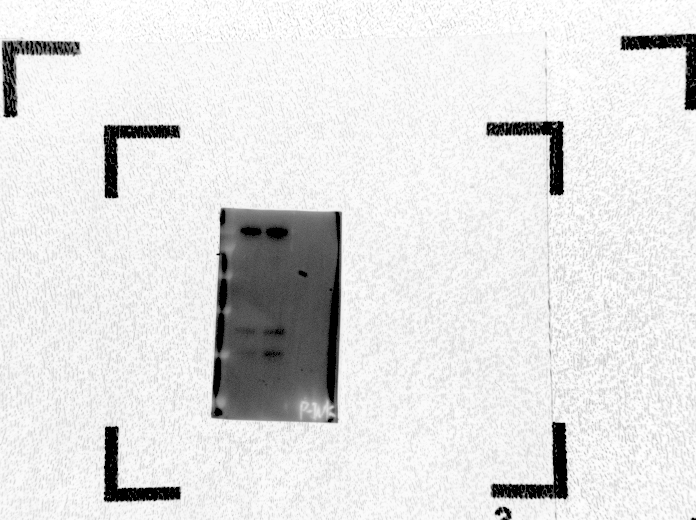

Supplement: Supplementary file 1 [file cells-10-02818-s001.zip › cells-1374880/The full bolt images for the Western Blot/P-JNK/P-JNK(down) and HSP90(up) -3.tif]

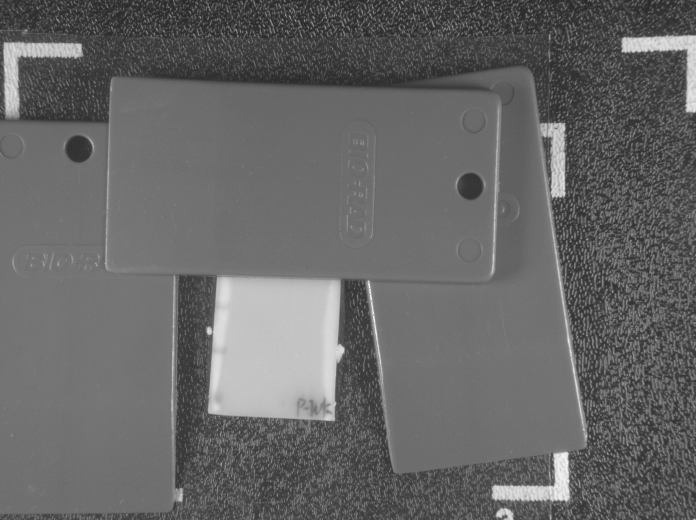

Supplement: Supplementary file 1 [file cells-10-02818-s001.zip › cells-1374880/The full bolt images for the Western Blot/P-JNK/P-JNK-1.tif]

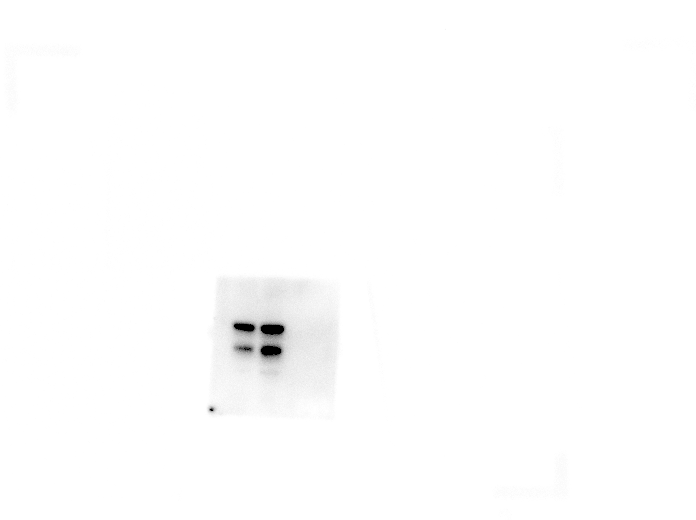

Supplement: Supplementary file 1 [file cells-10-02818-s001.zip › cells-1374880/The full bolt images for the Western Blot/P-JNK/P-JNK-2.tif]

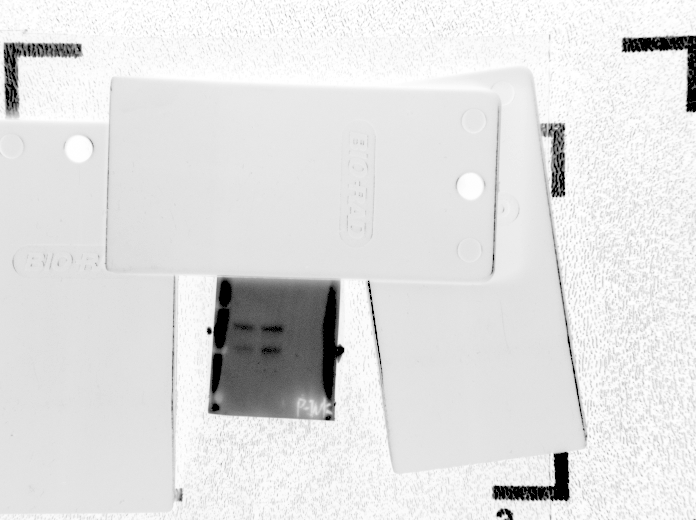

Supplement: Supplementary file 1 [file cells-10-02818-s001.zip › cells-1374880/The full bolt images for the Western Blot/P-JNK/P-JNK-3.tif]

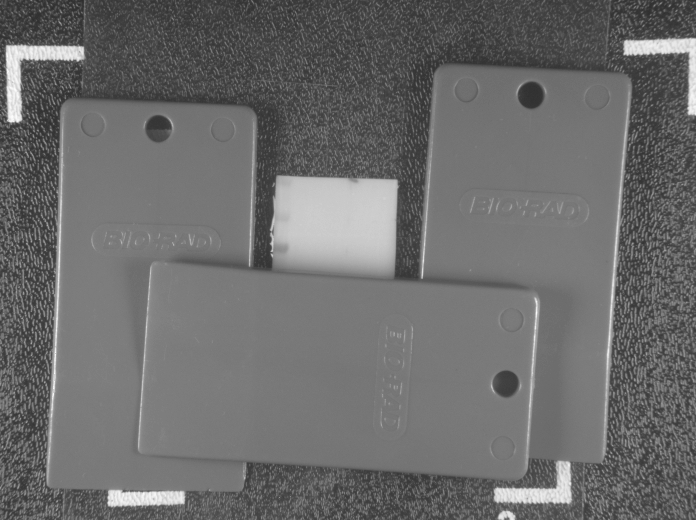

Supplement: Supplementary file 1 [file cells-10-02818-s001.zip › cells-1374880/The full bolt images for the Western Blot/p-P38/HSP90-1.tif]

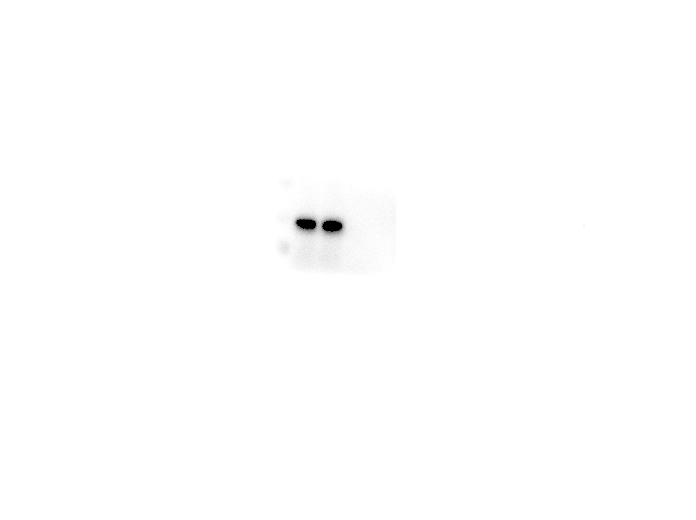

Supplement: Supplementary file 1 [file cells-10-02818-s001.zip › cells-1374880/The full bolt images for the Western Blot/p-P38/HSP90-2.tif]

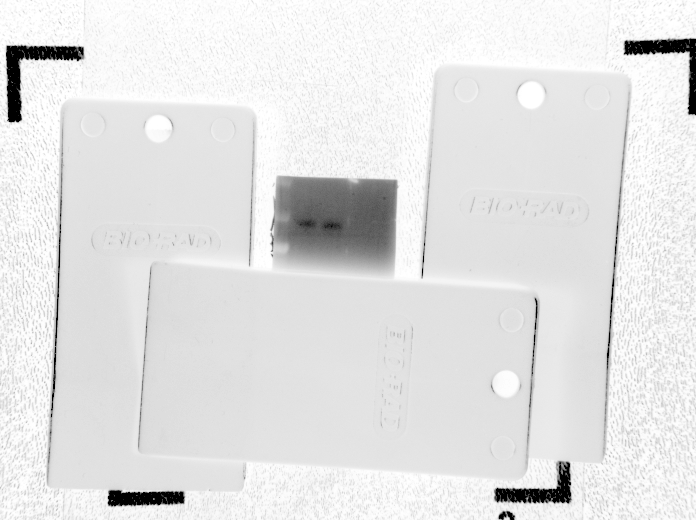

Supplement: Supplementary file 1 [file cells-10-02818-s001.zip › cells-1374880/The full bolt images for the Western Blot/p-P38/HSP90-3.tif]

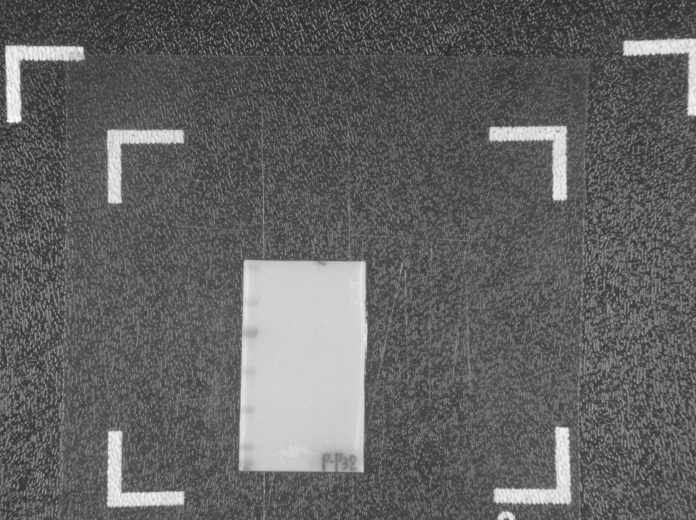

Supplement: Supplementary file 1 [file cells-10-02818-s001.zip › cells-1374880/The full bolt images for the Western Blot/p-P38/p-P38(down) and HSP90(up)-1.tif]
